# Molecular and *in silico* typing of the lipooligosaccharide biosynthesis gene cluster in *Campylobacter jejuni* and *Campylobacter coli*

**DOI:** 10.1371/journal.pone.0265585

**Published:** 2022-03-31

**Authors:** Amber Hameed, Julian M. Ketley, Alexandra Woodacre, Lee R. Machado, Gemma L. Marsden

**Affiliations:** 1 Centre for Physical Activity and Life Sciences, University of Northampton, Northampton, United Kingdom; 2 Department of Genetics and Genome Biology, University of Leicester, Leicester, United Kingdom; 3 Healthcare Infection Society, London, United Kingdom; Cornell University, UNITED STATES

## Abstract

The extensive genetic variation in the lipooligosaccharide (LOS) core biosynthesis gene cluster has led to the development of a classification system; with 8 classes (I-VIII) for *Campylobacter coli* (*C*. *coli*) LOS region and with 23 classes (A-W) or four groups (1–4) for *Campylobacter jejuni* (*C*. *jejuni*) LOS region. PCR based LOS locus type identification for *C*. *jejuni* clinical isolates from a UK hospital as well as *in silico* LOS locus analysis for *C*. *jejuni* and *C*. *coli* genome sequences from GenBank was carried out to determine the frequencies of various LOS genotypes in *C*. *jejuni* and *C*. *coli*. Analysis of LOS gene content in 60 clinical *C*. *jejuni* isolates and 703 *C*. *jejuni* genome sequences revealed that class B (Group 1) was the most abundant LOS class in *C*. *jejuni*. The hierarchy of *C*. *jejuni* LOS group prevalence (group 1 > group 2 > group 3 > group 4) as well as the hierarchy of the frequency of *C*. *jejuni* LOS classes present within the group 1 (B > C > A > R > M > V), group 2 (H/P > O > E > W), group 3 (F > K > S) and group 4 (G > L) was identified. *In silico* analysis of LOS gene content in 564 *C*. *coli* genome sequences showed class III as the most abundant LOS locus type in *C*. *coli*. *In silico* analysis of LOS gene content also identified three novel LOS types of *C*. *jejuni* and previously unknown LOS biosynthesis genes in *C*. *coli* LOS locus types I, II, III, V and VIII. This study provides *C*. *jejuni* and *C*. *coli* LOS loci class frequencies in a smaller collection of *C*. *jejuni* clinical isolates as well as within the larger, worldwide database of *C*. *jejuni* and *C*. *coli*.

## Introduction

*Campylobacter* is a foodborne enteropathogen which transmits to humans mainly by consumption of *Campylobacter* contaminated dairy products and raw or partially cooked meat [1, 2]. Most *Campylobacter* species, including *C*. *jejuni*, *C*. *coli*, *C*. *lari*, *C*. *fetus*, *C*. *upsaliensis*, *C*. *hypointestinalis*, *C*. *helveticus*, *C*. *lanienae*, and *C*. *mucosalis* are found in warm-blooded animals [2–7]. However, some *Campylobacter* species, such as *C*. *fetus* and *C*. *geochelonis* can also occur in cold-blooded reptiles (lizard, tortoise, and snake) [8, 9]. Chicken is the main reservoir of *Campylobacter* through intestinal colonisation after hatching, usually at the age of 2–5 weeks [10–12]. Chickens contaminated with *Campylobacter* (with approximately 10^9^ CFU/g caecal contents) are considered as a major source of *Campylobacter* transmission to humans [13, 14]. *Campylobacter* isolates can also be present as environmental contamination in non-livestock niches, which may be agricultural or non-agricultural [15–18].

The estimated number of cases of *Campylobacter* infection is approximately 96 million per year worldwide [19]. *Campylobacter* infection is characterised by an acute, self-limiting gastroenteritis in humans which causes various clinical symptoms including watery or bloody diarrhoea, abdominal pain, headache, fever, chills, and dysentery [20–22]. In some cases, neuronal disorders Guillain–Barré syndrome (GBS) and Miller Fisher syndrome (MFS), Reiter’s arthritis, and irritable bowel syndrome can also occur postinfection [23, 24].

Lipooligosaccharide (LOS) is an integral component of the outer cell membrane of *Campylobacter* and is synthesised by a cluster of lipooligosaccharide biosynthesis genes [25–27]. Each LOS biosynthesis gene present in this cluster produces an individual enzyme either for monosaccharide biosynthesis or addition of a particular monosaccharide to the LOS structure [28–31]. The LOS inner core biosynthesis genes are less variable and flank a highly variable central region of LOS outer core (OC) biosynthesis genes [27, 28, 32]. At one flank are *waaC*, *waaM*, and *lgtF* (also known as *cj1135*), and at the other, *waaV*, *waaF*, *gmhA*, *waaE*, *waaD*, *gmhB*, and *cyf* (also known as *cj1153*); these IC biosynthesis genes occur in the same order in almost all *C*. *jejuni* and *C*. *coli* strains. However, the OC biosynthesis gene cluster that extends from *lgtF* and *waaV* varies extensively among *C*. *jejuni* and *C*. *coli* strains and this region can acquire new genes by horizontal gene transfer during infection inside an animal host [33, 34]. Insertion or deletion events in this gene region give rise to a new LOS locus organisation or type in *Campylobacter* strains, which can be variable both in gene content and gene organisation [29, 35]. Disruption in resident LOS biosynthesis genes and allelic variation can also contribute to new LOS types [29]. A *C*. *jejuni* LOS class comprises a specific organisation of LOS genes named alphabetically; altogether 23 *C*. *jejuni* LOS classes (A through W) were previously described [27, 30, 32]. An updated and simplified classification system with categorisation of the previously described 23 *C*. *jejuni* LOS classes into four LOS groups (1–4) was presented in order to better understand the prevalence of *C*. *jejuni* LOS groups and group-related LOS classes [36]. Group 1 includes all those LOS locus types, A, B, C, R, M and V, which contain the sialic acid biosynthesis genes (*neuA1*, *neuB1*, *neuC1* and *cst*-II/*cst*-*III*) whereas the other three groups had LOS loci with no sialic acid biosynthesis genes. Five classes (E, H, O, P and W) in LOS group 2, eight classes (D, F, K, Q, N, I, J, and S) in LOS group 3 and four classes (L, G, T, and U) in LOS group 4 were assimilated [36]. Variations in the LOS OC biosynthesis gene content cause modifications in the LOS OC structure [37, 38]. The LOS structures with variable OC epitopes help *Campylobacter* evade the host immune system as they mimic the human gangliosides [36, 38–42]. For this reason, antibodies produced against the LOS structural epitopes not only bind to LOS structures, but also to human gangliosides [33, 38, 39, 43]. The cross-reactivity of anti-LOS antibodies with human gangliosides is a critical contributory factor that leads to the development of GBS or MFS in humans [23, 24, 44]. Thus, the expression of variable cell surface LOS structures due to variable gene content in the LOS locus in *C*. *jejuni* is considered an important virulence factor and may have direct connection with the progression of different neural disorders [37, 45]. For example, *C*. *jejuni* strains with LOS locus class A and variable human ganglioside mimics (GM1a, GM1b, GD1a, and GD1b) may help *C*. *jejuni* to trigger GBS post-infection in *Campylobacter* infected patients [38, 40, 41, 46]. In contrast, *C*. *jejuni* strains with LOS class B and corresponding GQ1b-like LOS structures are suggested to be associated with MFS in *Campylobacter* infected patients [38, 47]. Based on the strong relationship between the variable LOS synthesis region and *C*. *jejuni* virulence, the current study aimed to determine the frequency of *C*. *jejuni* LOS genotypes from whole genome sequences present within GenBank (n = 703), as well as in *C*. *jejuni* clinical isolates (n = 60) from a UK hospital in order to further estimate the extent of gene variation in the *C*. *jejuni* LOS biosynthesis gene region and identify any novel *C*. *jejuni* LOS locus types. We previously analysed data from the literature relevant to the abundance of *C*. *jejuni* LOS classes in various geographical areas of the world to provide an overview of *C*. *jejuni* LOS genotype predominance [36]. The present study found that the prevalence of *C*. *jejuni* LOS group 1 classes (M, R, & V), group 2 class W, group 3 classes (K, Q, N, I, S & J) and all classes of group 4 (L, G, T & U) has never been identified before, which may be because of the relatively small size of collections involved in the previous studies. This study has for the first time presented the distribution of all, previously known LOS classes of *C*. *jejuni* using publicly accessible GenBank database.

Eight previously established *C*. *coli* LOS classes have been described I-VIII [32]. The relationship between different *C*. *coli* LOS locus structures and virulence is not fully established [48]. This is due to the presence of a wide variety in *C*. *coli* LOS biosynthesis locus types and limited knowledge of how this results in *C*. *coli* LOS structures at molecular level [32, 49]. As a result, we were interested in identifying the LOS locus types which dominate and are frequently present in *C*. *coli* isolates. To achieve this, we determined the frequency of *C*. *coli* LOS types present in GenBank which represents a global database of *C*. *coli* genome sequence deposits (n = 564). A few studies have been carried out previously to estimate the distribution of *C*. *coli* LOS classes present within the collections of livestock associated *C*. *coli* strains (n = 33) and agriculture related *C*. *coli* strains (n = 261) [32, 50]. This study presents an up-to-date picture of LOS genotype predominance in *C*. *coli* and identifies previously unreported *C*. *coli* LOS biosynthesis genes. The overall aim of this work was to provide a hierarchy of the frequency of LOS classes and identify prevalence of any novel LOS genes in *C*. *jejuni* and *C*. *coli*. This will aid investigation of increasingly complex levels of LOS variation and the role such variation plays in *Campylobacter* infection.

## Results

### Identification of frequency of *C*. *jejuni* LOS locus classes in GenBank database

**Fig 1:**
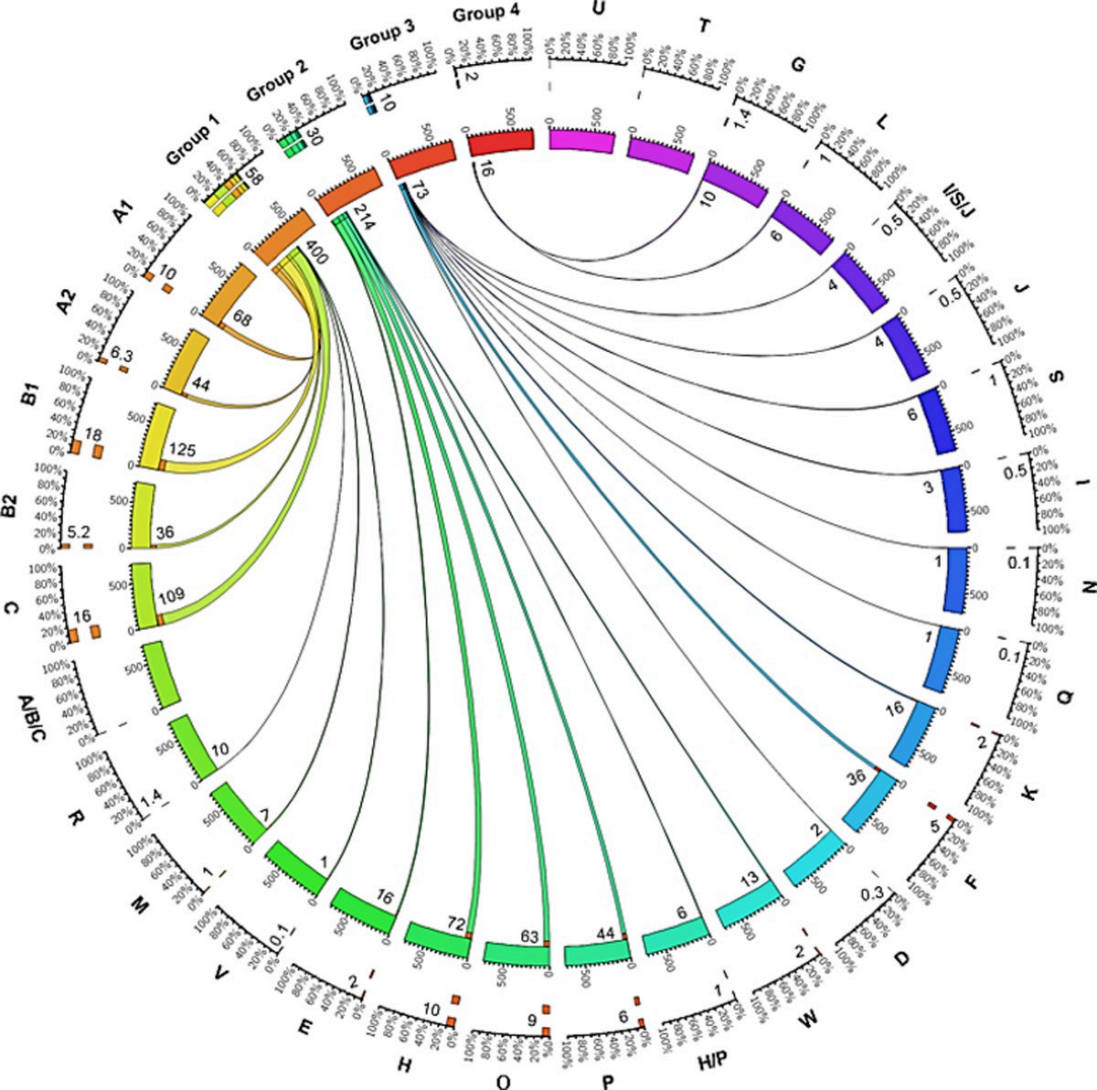
A Circos plot showing the distribution of *C*. *jejuni* LOS locus classes (A-W), subclasses (A1, A2, B1, B2) and LOS groups (1–4) in the online *C*. *jejuni* sequence database. Each segment of the inner circle specifies the total number of *C*. *jejuni* strains sequences (703) extracted for the LOS classification. The frequency of *C*. *jejuni* isolates classified for each particular LOS class/group is mentioned in numbers (n out of 703) on the top of each inner circle segment and represented with ribbon width. The frequency of a *C*. *jejuni* LOS class/group in percentage (% of overall frequency-100) is shown with each outer circle segment and represented by the orange or coloured bars. Ribbons link each *C*. *jejuni* LOS class to its related LOS group.

With the routine upload of whole genome sequences of *Campylobacter* spp. in Genbank we aimed to determine the frequencies of *C*. *jejuni* LOS locus classes and LOS groups in a collection of 703 *C*. *jejuni* GenBank sequences by *in silico* analysis (Fig 1 and S1 Table online).

58% (n = 400 of 703) of *C*. *jejuni* sequences belonged to the LOS group 1. The LOS classes A, B, and C adopted a hierarchy with class B1 (n = 125; 18%) > C (n = 109; 16%) > A1 (n = 68; 10%) > A2 (n = 44; 6%) > B2 (n = 36; 5%), were the most common LOS group 1 related classes. Other group 1 related classes including R (n = 10; 1.4%), M (n = 7; 1%), and V (n = 1; <1%) were rare. 30% (n = 214) of sequences were positive for either class E (n = 16; 2%), class H (n = 72; 10%), class O (n = 63; 9%), class P (n = 44; 6%) or class W (n = 13; 2%) and therefore, belonged to LOS group 2. 10% (n = 73) of *C*. *jejuni* sequences were positive for LOS group 3 classes including D (n = 2; <1%), F (n = 36; 5%), K (n = 16; 2%), Q (n = 1; <1%), N (n = 1; <1%), I (n = 3; <1%), S (n = 6; 1%) and J (n = 4; <1%). Only 2% of strains (n = 10 and n = 4 positive for class G and L, respectively) belonged to group 4.

### Identification of frequency of *C*. *jejuni* LOS locus classes among *C*. *jejuni* clinical isolates

**Fig 2:**
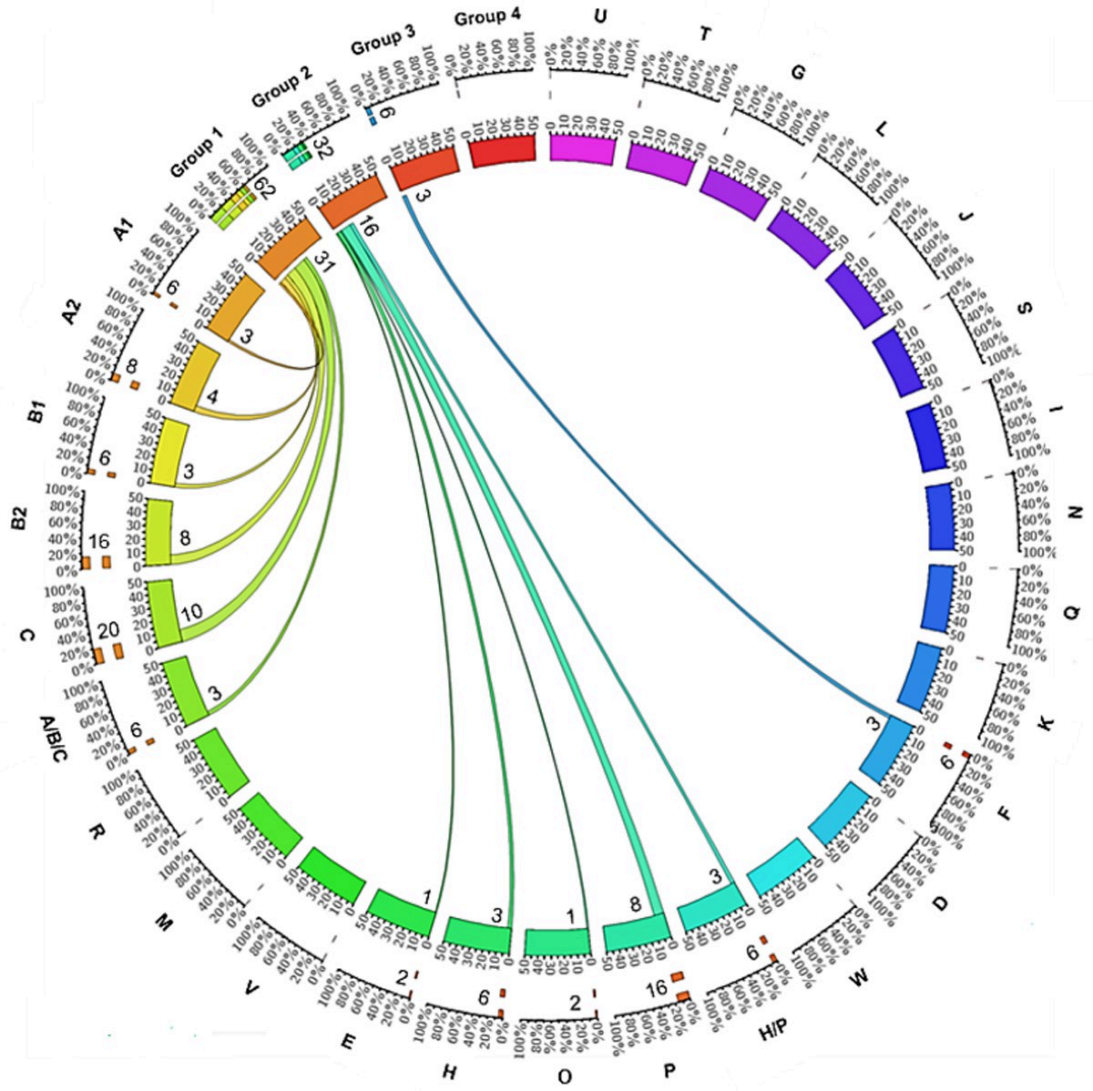
A Circos plot showing the distribution of *C*. *jejuni* LOS locus classes (A-W), subclasses (A1, A2, B1, B2) and LOS groups (1–4) from clinical isolates. Each segment of the inner circle specifies the total number of classified *C*. *jejuni* isolates (50) used for the PCR based typing assay. The frequency of *C*. *jejuni* isolates classified for each particular LOS class/group is mentioned in numbers (n out of 50) on the top of each inner circle segment and represented with ribbon width. The frequency of a *C*. *jejuni* LOS class/group in percentage (% of overall frequency-100) is mentioned with each outer circle segment and represented by the orange or coloured bars. Ribbon ends link each *C*. *jejuni* LOS class to its related LOS group.

We were interested in examining whether Genbank LOS sequences were representative of LOS sequences present more locally. To address this, gDNA from 60 clinical *C*. *jejuni* isolates was extracted and the origin of DNA only from *C*. *jejuni* strains was confirmed by performing PCR reactions with *waaM* and *waaV* LOS gene specific control primers. Each DNA sample, positive for *waaM* and *waaV* LOS genes, was assigned with a LOS class based on PCR and Sanger sequencing results (S5 Table online). 50 of 60 *C*. *jejuni* isolates were typeable while remaining 10 were non-typeable. 6 of 50 (12%) classified *C*. *jejuni* strains (54386, S2, 92691, 118973, 118715 and 93133Y) were PCR positive for more than one LOS class. The frequency of *C*. *jejuni* LOS locus classes (A through W), subclasses (A1, A2, B1, B2) and LOS groups (1–4) prevalent in a *C*. *jejuni* clinical strains collection was determined (Fig 2). 62% (n = 31) of *C*. *jejuni* strains belonged to group 1 LOS classes. This included A1 (n = 3; 6%), A2 (n = 4; 8%), B1 (n = 3; 6%), B2 (n = 8; 16%), C (n = 10; 20%), and a mixed ABC class (n = 3; 6%). No *C*. *jejuni* strain was positive for other group 1 related classes (M, R, V). 32% (n = 16) of *C*. *jejuni* strains were positive for group 2 classes. E (n = 1; 2%), H (n = 3; 6%), O (n = 1; 2%), P (n = 8; 16%) or a mix HP (n = 3; 6%) were identified. Only 6% (n = 3) *C*. *jejuni* strains were assigned to the LOS group 3 related class F. No *C*. *jejuni* strain was associated with other group 3 classes (D, K, Q, N, I, S, J). In addition, *C*. *jejuni* strains with the LOS group 4 classes, L, G, T, & U, were absent from the clinical *C*. *jejuni* isolates.

**Fig 3:**
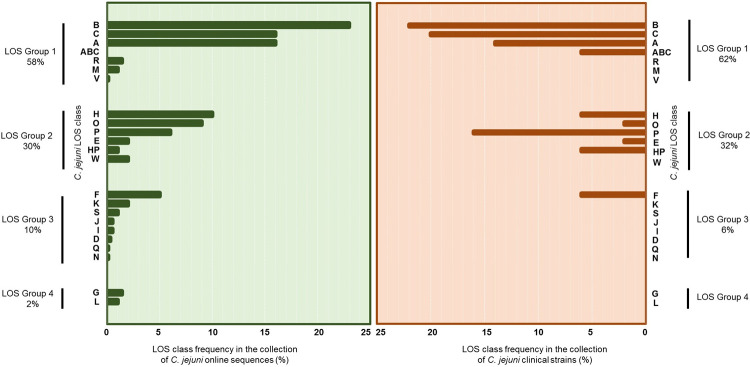
A comparison of *C*. *jejuni* LOS biosynthesis locus class and group frequencies found in the collections of GenBank *C*. *jejuni* sequences (n = 703) and *C*. *jejuni* clinical, typed strains (n = 50).

A comparison of *C*. *jejuni* LOS class and group frequencies, identified in both collections of clinical *C*. *jejuni* isolates and online *C*. *jejuni* sequences reveal that the frequencies of different classes were comparable between the two sources except class P (Fig 3).

### Identification of novel LOS biosynthesis locus types in *C*. *jejuni*

**Fig 4:**
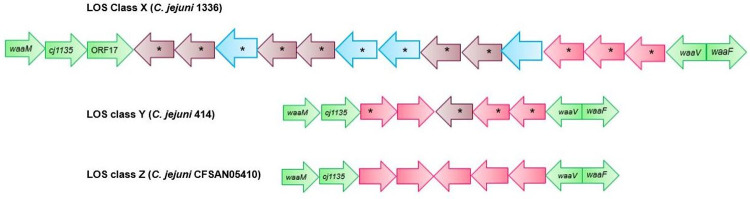
The genetic organisation of *C*. *jejuni* 1336, *C*. *jejuni* 414, and *C*. *jejuni* CFSAN05410 that contain novel LOS gene content. 13 LOS biosynthesis genes in *C*. *jejuni* 1336, 5 LOS biosynthesis genes in *C*. *jejuni* 414 and 5 LOS biosynthesis genes in *C*. *jejuni* CFSAN054107 occurred between previously known LOS genes (*cj1135*, ORF17, and *waaV*). Green arrows: previously reported LOS genes in *C*. *jejuni* strains; Pink arrows: previously known, variable LOS genes that had similarity to the LOS biosynthesis genes of other *C*. *jejuni* strains; Blue arrows: LOS genes that had similarity to the CPS biosynthesis genes of other *C*. *jejuni* strains; Purple arrows: LOS genes that had similarity to the LOS biosynthesis genes of *C*. *coli* strains. The direction of arrow represents the direction of gene transcription. Black star: Gene with an unknown function.

*C*. *jejuni* 1336 (Accession no: CM000854.1), *C*. *jejuni* 414 (Accession no: ADGM01000014.1) and *C*. *jejuni* CFSAN054107 (Accession no: CP028185.1) were found with novel LOS gene organisations or LOS types which were designated as class X, class Y and class Z respectively. The LOS biosynthesis region present between previously known LOS genes (*cj1135*, ORF17 and *waaV*) contained 13 LOS biosynthesis genes in *C*. *jejuni* 1336, 5 in *C*. *jejuni* 414 and 5 in *C*. *jejuni* CFSAN054107 (Fig 4).

Functions of 12 of 13 *C*. *jejuni* 1336 LOS genes are not known while one of them encodes an aminotransferase (WbdK). Functions of 4 of 5 *C*. *jejuni* 414 LOS genes are unknown while one of them encodes FkbM family methyltransferase. In *C*. *jejuni* CFSAN054107, 4 of 5 LOS genes encode glycosyltranferases and the remaining one encodes a methlytransferase (data from GenBank database). Further, the origin of these LOS novel genes was predicted by blast searching each gene against all sequences available in GenBank. 4 LOS genes in *C*. *jejuni* 1336 locus had >99% similarity with the capsular polysaccharide biosynthesis (CPS) genes of other *C*. *jejuni* strains, suggesting a possible gene transfer of CPS loci from other *C*. *jejuni* strains to the *C*. *jejuni* 1336 LOS locus. Six LOS genes of *C*. *jejuni* 1336 and one gene of *C*. *jejuni* 414 had no identity with the previously known *C*. *jejuni* LOS genes. Instead, they had >99% similarity with various LOS biosynthesis genes of *C*. *coli*, suggesting interspecies gene recombination events.

### Identification of frequency of *C*. *coli* LOS locus types in GenBank database

**Fig 5:**
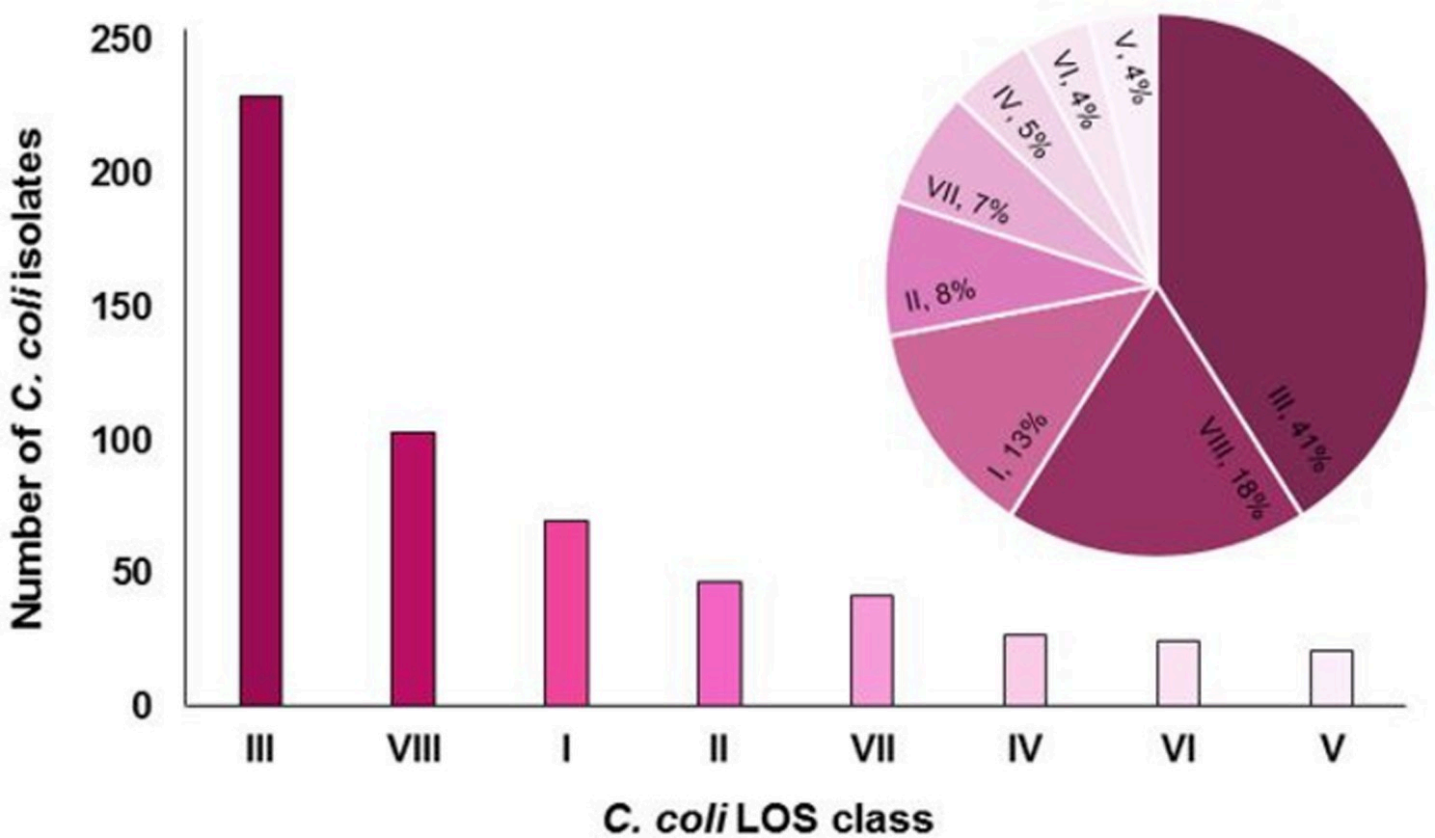
Distribution of *C*. *coli* LOS locus classes within the online *C*. *coli* sequences GenBank database. The number of *C*. *coli* strains associated with each *C*. *coli* LOS class in 2D column chart and corresponding percentages of *C*. *coli* strains in Pie chart, represent the frequency of *C*. *coli* LOS locus classes within the global collection of *C*. *coli* isolates.

The frequency of different *C*. *coli* LOS locus types (Fig 5) present in the online, publicly available GenBank database was identified by *in silico* analysis of 564 *C*. *coli* sequences (S3 Table online). *C*. *coli* LOS class III (41%; n = 229) was the most abundant in the GenBank database followed by class VIII (18%; n = 103), class I (13%; n = 70), class II (8%; n = 47), class VII (7%; n = 42), class IV (5% (n = 27), class VI (4%; n = 25) and class V (4%; n = 21).

### Identification of previously unknown LOS biosynthesis genes in *C*. *coli*

**Fig 6:**
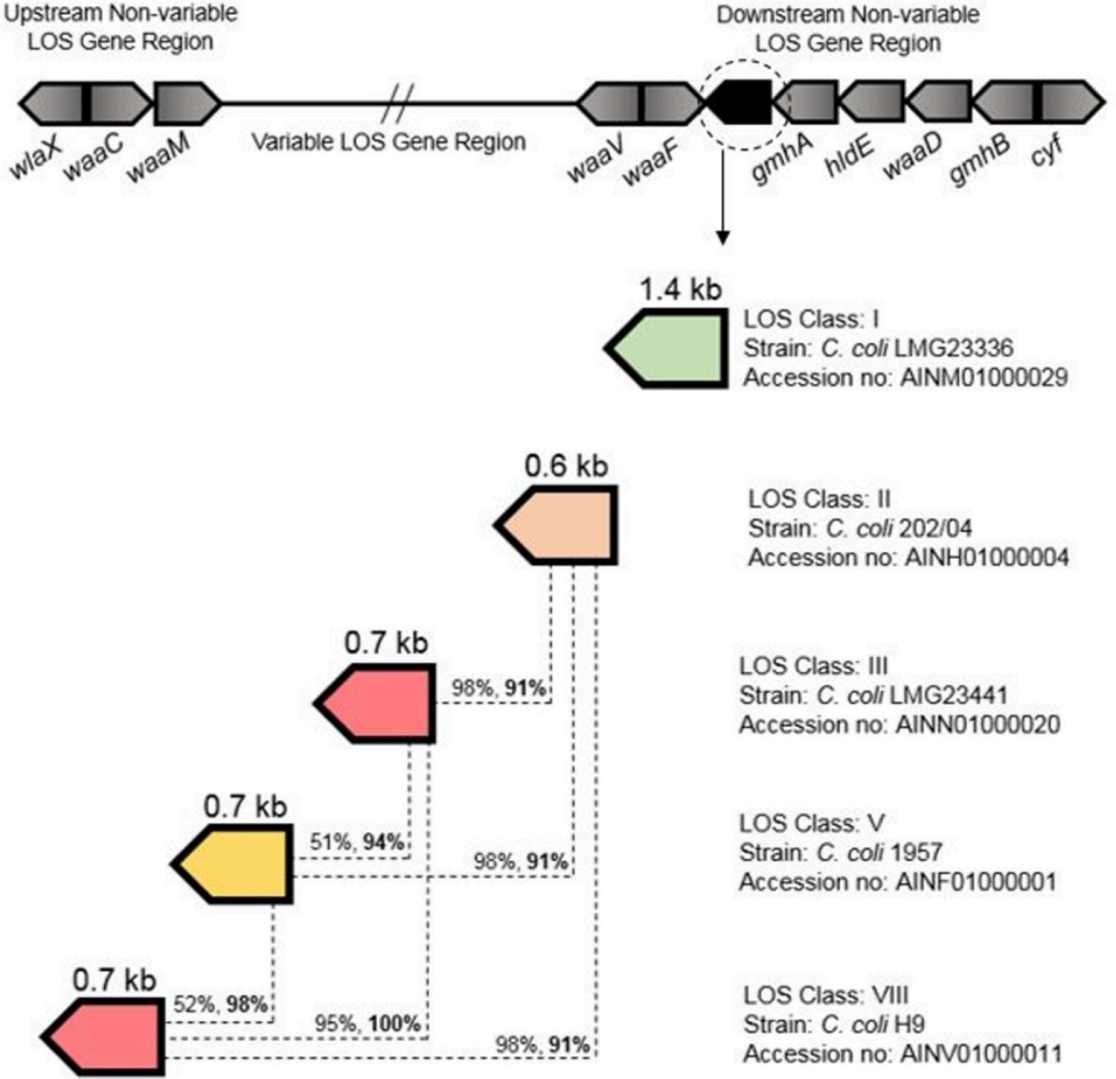
Genomic organisation of previously unreported *C*. *coli* LOS core genes and their relative similarity. The position of novel gene content between *waaF* and *gmhA* in *C*. *coli* LOS types (I, II, III, V and VIII). The sizes of these genes, obtained from the GenBank database, are given in kb. Each dotted line links two genes to represent the similarity between them in terms of query cover score (non-bold) and Megablast identity score (bold).

Genes at the LOS biosynthesis cluster’s one flank (*waaC*, *waaM*, *lgtF*) and at the other (*waaV*, *waaF*, *gmhA*, *waaE*, *waaD*, *gmhB*) are present with the same order or organisation in almost all *Campylobacter* strains and are involved in the biosynthesis of the LOS inner core [30, 49]. LOS class W (*C*. *jejuni* M1) [32] and class E (*C*. *jejuni* 81116) contain two genes between *waaF* and *gmhA* while class B (*C*. *jejuni* 81–176) contains a single gene between *waaF* and *gmhA*. Similarly, previously unreported LOS biosynthesis genes, localised between *waaF* and *gmhA* in the distal end of five *C*. *coli* LOS locus types (I, II, III, V, VIII) were found in this study. Each *C*. *coli* LOS type had insertion of one LOS biosynthesis gene between *waaF* and *gmhA* (Fig 6, also given with *C*. *coli* reference strains in S4 Table online). These LOS genes are present at the same position in all five types of *C*. *coli* LOS locus but vary in size and nucleotide composition. The class I gene has a maximum size of ~1.4 kb and is distantly related to those genes which occur at the same position in other *C*. *coli* LOS locus types (II, III, V & VIII). The class II gene has similarity (~91%) to genes present in other classes (III, V and VIII). Type III and VIII genes are found to be identical (100%) and the class V gene is partially (~51%) similar to these identical genes. The presence of these previously unreported LOS biosynthesis genes was confirmed in 436 *C*. *coli* GenBank sequences (grey coloured columns in S3 Table online) within the LOS type I (n = 63), II (n = 43), III (n = 229), V (n = 3), and VIII (n = 98).

The Class I gene encodes a β-Kdo transferase, class II gene encodes a phosphoheptose isomerase and genes in other classes produce glycosyltranferases for LOS synthesis (Data extracted from GenBank; S4 Table online).

### Association of *C*. *jejuni* and *C*. *coli* LOS loci distribution to *Campylobacter* sources

**Fig 7:**
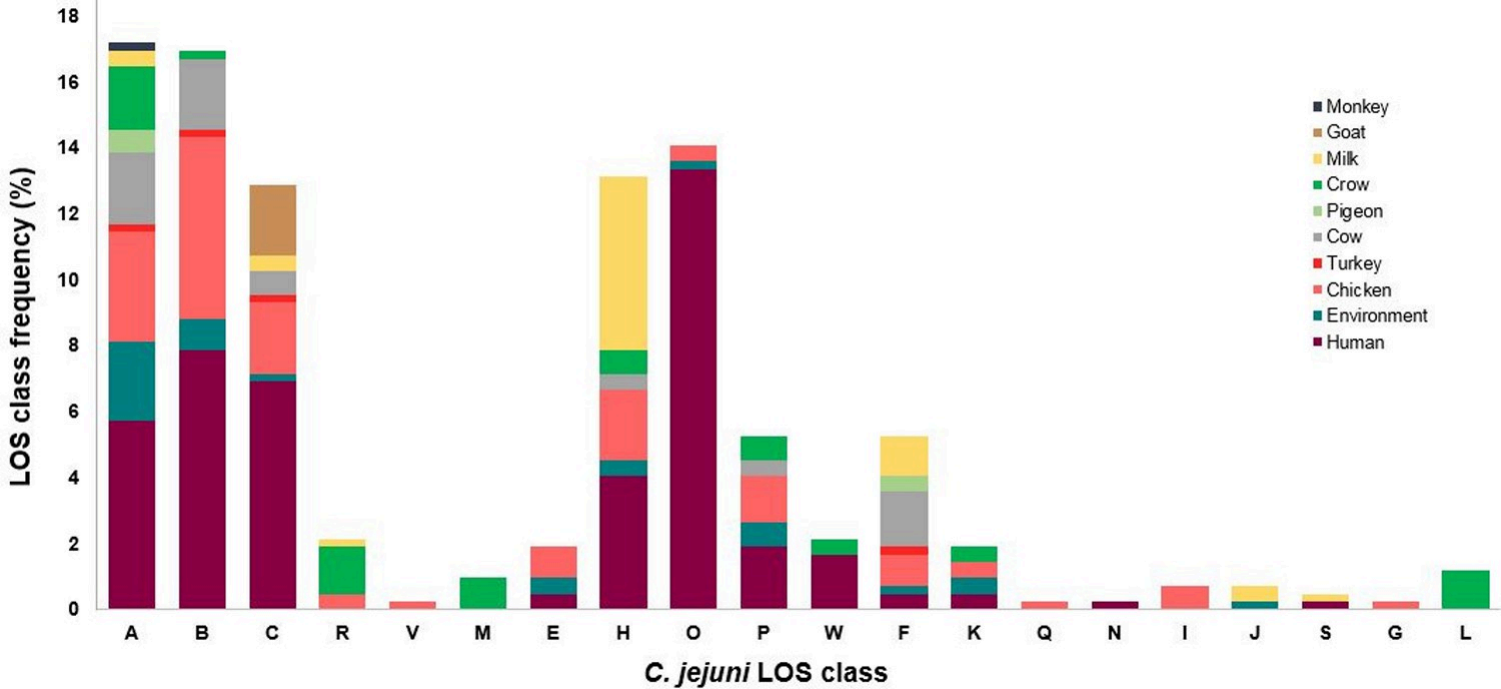
Frequency of *C*. *jejuni* LOS locus classes in different *Campylobacter* sources. Environment involves animal farm soil and animal farm water as *Campylobacter* sources.

**Fig 8:**
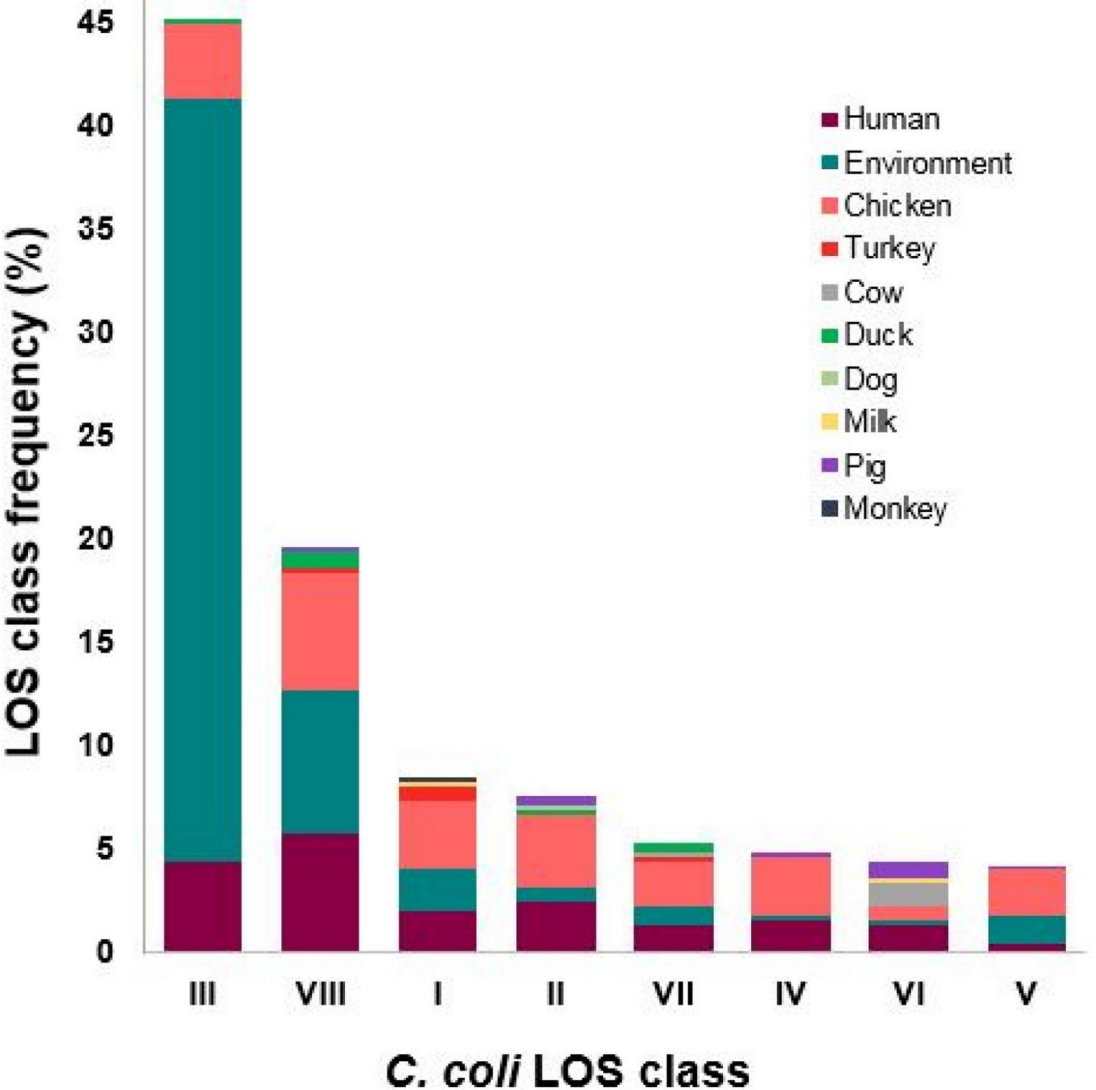
Frequency of *C*. *coli* LOS locus classes in different *Campylobacter* sources. Environment involves animal farm soil and animal farm water as *Campylobacter* sources.

The source of microbe isolation is usually specified with each sequence recorded in GenBank. The prevalence of *C*. *jejuni* (Fig 7) and *C*. *coli* (Fig 8) LOS genotypes in different *Campylobacter* niches was estimated by examining the online published sources (also given in S1 and S3 Tables online) of these *C*. *jejuni* and *C*. *coli* strains. *C*. *jejuni* and *C*. *coli* strains, isolated from faecal samples, were not included in this analysis as the actual source was unknown. The frequency of LOS genotypes within the pool of human *C*. *jejuni* isolates with online sequence data was comparable to the frequency of LOS genotypes identified within the collection of *C*. *jejuni* clinical isolates. For example, *C*. *jejuni* strains with LOS class F were common in our clinical isolates in comparison to other group 3 LOS classes (K, Q, N, I, J, S) and similarly, most abundantly isolated from humans prior to submit their genome sequences in GenBank. *C*. *jejuni* LOS group 1 associated *C*. *jejuni* strains were found mostly in humans [B (n = 33; 8%) > C (n = 29; 7%) > A (n = 24; 6%)] and chickens [B (n = 23; 5%) > A (n = 14; 3%) > C (n = 9; 2%)]. Humans, chickens, and the animal farm environment were the common isolation sources for *C*. *jejuni* and almost every *C*. *jejuni* LOS class was associated with at least one of these sources. Moreover, the most predominant LOS class III related *C*. *coli* strains were largely isolated from animal farm environments (n = 165; 37%), as well as from humans (n = 20, 5%) and chickens (n = 16, 4%), indicating that these are the common niches for type III LOS locus containing *C*. *coli* strains. The second most prevalent LOS class VIII was also frequent in the environment (n = 31, 7%) and at similar levels to isolates from humans (n = 26, 6%) and chickens (n = 25, 6%). These results indicated that chicken and humans are the most common hosts for *C*. *jejuni* while animal farm environments (particularly farm water and soil) is the most common niche for *C*. *coli*.

## Discussion

By analysing the distribution of *C*. *jejuni* LOS locus types, we determined that the frequency of LOS classes within the LOS group 1 was similar in both collections of *C*. *jejuni* online sequences [class B (23%) > class C (16%) ≥ class A (16%)] and *C*. *jejuni* clinical isolates [class B (22%) > class C (20%) > class A (14%)]. The LOS class B was the most common class in *C*. *jejuni* isolates from a clinical cohort as well as in the *C*. *jejuni* GenBank database. It has been reported previously in other studies [29, 47, 51–53] as the most common LOS class in humans as well as poultry *C*. *jejuni* isolates. The current study highlights that class C is the second most abundant LOS locus class in *C*. *jejuni*. Many other studies have described the LOS class C as the major class of *C*. *jejuni* LOS biosynthesis locus [54, 55]. However, in comparison to the high prevalence of LOS class C (42%) in clinical isolates in Sweden [55], a very small number of clinical strains (2%) in Bangladesh had association with LOS locus C [47]. Most of the GBS-related *C*. *jejuni* strains in Bangladesh and China possessed the LOS locus class A rather than class C [47, 56, 57], suggesting that *C*. *jejuni* LOS class distribution is likely to vary geographically. High frequencies of classes B and C may be present due to their ability to encode heterogenous ganglioside mimics, which is advantageous for pathogenesis [58, 59]. The frequency of sialic acid biosynthesis LOS loci (A, B & C) in the online *C*. *jejuni* GenBank database as well as among the clinical *C*. *jejuni* isolates was high, although the former collection contained other *C*. *jejuni* sources (e.g., animals, birds and farm soil) in addition to human. It demonstrates that LOS group 1 containing LOS locus classes A, B, and C are commonly present in every type of *C*. *jejuni* source population. We have described this earlier in our literature review [36]. The low rate of GBS and MFS in *Campylobacter* infected patients [41, 46] despite high predominance of GBS/MFS associated LOS classes (A, B, and C) in the clinical cohort supports the notion that some other factors in addition to LOS structures significantly contribute to the development of these neural diseases [35, 36, 47]. Other LOS classes from group 1 (R, V and M), also contain genes for the biosynthesis of sialylated LOS structures [30, 32, 42], but they were absent from the clinical isolates, and this may reflect the fact that, even in a large repository, sequences belonging to these classes represented only 2.5% of the online database. The reasons for poor distribution of these classes remain unclear. 16% of LOS typed *C*. *jejuni* clinical strains had the LOS group 2 related class P and 10% of analysed *C*. *jejuni* sequences belonged to the LOS group 2 related class H, marking the class P in our local clinical *C*. *jejuni* collection and class H in the online *C*. *jejuni* sequence database as the most predominant LOS group 2 classes. These contrasting results might be explained because both LOS class P and H share almost similar gene content except for two LOS biosynthesis genes (Orf 26’ and Orf28) [57] and therefore only a few studies [47, 52] have ever considered these classes as two separate classes. LOS group 2 appeared as the second most abundant group among a small population of *C*. *jejuni* from the enteritis cases, although these types of loci lack the sialic acid biosynthesis genes and likely produce non-sialylated LOS structures [30, 60]. Therefore, LOS sialylation appears to be advantageous when present, but may not be absolutely critical for Campylobacter survival in animal hosts. Ganglioside-like structures other than GM1 or GQ1b were infrequent in LOS locus E associated *C*. *jejuni* strains as evidenced using serological assays [40]. However, the prevalence was low and structural assays were not employed. Therefore, it currently remains unclear whether ganglioside mimicry is produced in any non-group 1 strains. Within the LOS group 3, class F was the most prevalent class among *C*. *jejuni* clinical isolates (6%) and in the online sequence database (5%). Class K (2%) was the second most common class of group 3, whereas other LOS classes (I, S, J, D, Q, and N) were less frequent (≤1%) in GenBank database. *C*. *jejuni* sequences in a very small number (<3%) belonged to group 4. In contrast, no group 3 related classes (except for class F) and group 4 related classes were identified from *C*. *jejuni* clinical isolates, which may be because of the relatively small size of the collection. The hierarchy of LOS group prevalence was group 1 > group 2 > group 3 > group 4 in both types of collections of *C*. *jejuni* isolates.

Six *C*. *jejuni* strains were found positive for more than two LOS classes using PCR, which occur due to co-infection in patients with multiple *C*. *jejuni* strains. The co-infection occurrence with multiple *C*. *jejuni* strains has been previously observed in GBS patients [61]. Another reason could be the occurrence of LOS gene recombination during infection as it has been observed previously [33, 34]. The *in-silico* prediction for the presence of six *C*. *coli* LOS genes in *C*. *jejuni* 1336 and one *C*. *coli* LOS gene in *C*. *jejuni* 414 highlight the occurrence of interspecies genes recombination events. *C*. *jejuni* does not only harbour the genes from *C*. *coli*, but *C*. *coli* can also uptake and acquire *C*. *jejuni* DNA, especially when they are present in the same niche [18]. *C*. *jejuni* and *C*. *coli* share 71% of LOS biosynthesis genes and 65% of CPS biosynthesis genes because of recombination events [32].

LOS locus type III was abundant (41%; n = 229 of 564) in the online GenBank database of *C*. *coli* sequences. The high frequency of class III (28%; n = 72 of 261) within the agriculture-associated *C*. *coli* strains has been reported in a previous study [50]. The reasons behind the high prevalence of LOS class III in *C*. *coli* are yet to be investigated. *C*. *coli* strain 76339 contains the sialic acid biosynthesis genes (*cst-V*, *neuA*, *neuB*, & *neuC*) and sialic acids in its LOS structure [49, 62]. In the current study, all *C*. *coli* LOS sequences extracted from GenBank were scrutinised for the presence of these sialic acid biosynthesis genes and only *C*. *coli* RM4661 (Accession no: CP007181.1) was found to contain sialic acid synthesis genes (*cst*, *neuB* & *neuC*) in the LOS locus. However, *C*. *coli* LOS locus classes with *cst* alleles were identified in another study where XV-XXIV had *cst-II*; XIII and XIV had *cst-III* and IX, XXV, XXVI had *cst-V*. All these classes were positive for *neuABC* [49, 50]. Other LOS classes of *C*. *coli* including II, III, XXVII—XXXV had different alleles of *cst* (*cst-IV* and *cst-VI*) and only a small fraction of these classes (6.44% and 4.51% of strains positive with *cst-IV* and *cst-VI* respectively) had *neuABC* positioned outside of LOS locus [50]. Despite having genes (sialyltransferases) associated with the LOS sialylation, *C*. *coli* strains with ganglioside mimicry have not been reported so far [48–50, 63]. The most common LOS class (III) and the second most common class (VIII) linked *C*. *coli* strains were largely isolated from humans, which is concordant with a previous study, where half (57%) of the clinical isolates belonged to class III, VIII and II [64]. All *C*. *coli* classes tended to come from humans, chickens and the farm environment, suggesting that animal farm water and soil, in addition to chickens, are the primary sources of *C*. *coli* transmission to humans. This agrees with a previous study that reported agriculture associated *C*. *coli* as an emerging human pathogen [17, 18]. In this study, previously unreported LOS genes were identified in the *C*. *coli* LOS biosynthesis locus types I, II, III, V and VIII. The identified LOS biosynthesis genes in *C*. *coli* vary at the sequence and functional level among *C*. *coli* LOS locus classes (based on data available in GenBank). It highlights that other region of LOS biosynthesis locus also vary amongst *C*. *coli* strains in addition to the central region of LOS locus that can impact the LOS structure in *C*. *coli*. The sequence level variation in these genes and their possible putative functions have been determined *in silico* but require further functional characterisation.

The genome sequences deposited in GenBank may not be absolutely correct due to occurrence of errors at the experimental or sequence data analysis stages [65], and therefore can produce false-positive results. In this study, variation in LOS biosynthesis locus was analysed at the base sequence level to determine the presence of clustered whole LOS genes rather than finding the modifications between sequence bases. The identification of several LOS genes within a single genomic sequence reduces the possibility of false-positive results but does not eliminate it.

In conclusion, this work compares the frequency of various *C*. *jejuni* LOS locus classes in local versus global collections of *C*. *jejuni* isolates and provides an overview of predominance of various LOS biosynthesis gene clusters in the populations of *C*. *jejuni* and *C*. *coli*.

## Materials and methods

### Collection of bacterial strains and their growth

In a 12-month period from November 2015–2016, *C*. *jejuni* isolates (n = 60; S5 Table online) from anonymised clinical samples from Northampton General Hospital, UK were collected by swabbing cultured Charcoal-Cefoperazone-Deoxycholate Agar (CCDA) plates. Amines and charcoal swabs (Thermo Fisher Scientific) were used for collection and transportation of *Campylobacter* isolates. Bacterial isolates were cultured again within 24 hours of collection and grown on MHA plates at 37°C for 24–48 hours under a microaerobic atmosphere of 5% O_2_, 10% CO_2_ and 85% N_2_. The microaerobic environment was provided by either using CampyGen sachets (Oxoid Limited) in 2.5 L air-tight jars or BOC gas mixture (2% H_2_, 5% O_2_, 10% CO_2_ and 83% N_2_) in a Whitley G2 workstation (Don Whitley Scientific).

### DNA extraction from *C*. *jejuni* clinical strains

For the extraction of genomic DNA (gDNA) from *C*. *jejuni* isolates, the protocol provided by the DNeasy Blood and Tissue kit manufacturer (Qiagen) was followed.

### Designing of LOS class specific primers

25 *C*. *jejuni* LOS class specific PCR primer pairs including *waaM* and *waaV* LOS gene specific control primers (S2 Table online) were designed using Clone Manager Professional Suite (Version 8; Scientific & Educational Software, Morrisville, USA) and purchased from the Eurofins Genomics (Ebersberg, Germany). Each LOS class specific primer pair spanned the junction of two adjacent LOS genes and were specific to two LOS biosynthesis genes rather than a single gene.

### PCR for LOS typing

PCR master mix (20 μL) was prepared after mixing the template DNA (~50 ng) with forward and reverse primers (10μM each), MyTaq™ red DNA polymerase (0.25 μL containing 1.25 units; Bioline Reagents Ltd) and 5X MyTaq™ red Reaction Buffer (5 μL containing 5 mM dNTPs and 15 mM MgCl_2_; Bioline Regents Ltd). Each reaction was carried out in a thermocyler (TECHNE) using appropriate cycling conditions: 1 cycle of initial template denaturation (95°C; 5 min) followed by 35 cycles of template amplification (template denaturation at 95°C for 30 sec, primer annealing at optimised temperature for 35 sec, primer extension at 72°C for 30 sec per 1 kb of expected PCR product size) and one cycle of final extension (72°C for 5 min). PCR products were resolved by electrophoresis on 1% (w/v; dissolved in 1X TAE buffer) agarose gel, which were stained with SYBR^®^ safe stain (10,000X; Invitrogen) and visualised under UV light in G: Box (Syngene). PCRs with gDNA of reference *C*. *jejuni* strains (as indicated in S5 Table online) were used as positive controls and PCR with gDNA of *C*. *jejuni* 11168Δ32–52 (a mutant strain of *C*. *jejuni* NCTC11168 lacking LOS biosynthesis region from gene *cj1132*-*cj1152*) was used as a negative control in all PCR reactions.

### Sanger sequencing of PCR products

The protocol provided with the Eurofins Mix2Seq kit was followed for Sanger sequencing PCR products. According to the protocol, 15 μL purified PCR product (1–15 ng/μL) was mixed with 2 μL of either forward or reverse primer stock solution (10 pmol/μL). 17 μL DNA/primer mix was pipetted into a Mix2Seq tube and sent to Eurofins, Wolverhampton, U.K. for sequencing. Sequence data, obtained online in fasta format, was analysed using Clone Manager Professional Suite.

### Bioinformatic analysis

The genome sequences of 703 *C*. *jejuni* strains (125 complete; 578 draft) and 564 *C*. *coli* strains (22 complete; 542 draft) available on the 1^st^ March 2018 were obtained from GenBank and scrutinised by alignment using Megablast (https://blast.ncbi.nlm.nih.gov). The information related to the reference strains with previously defined LOS types (subject sequences) has been given in S2 and S4 Tables while for query sequences, used in this analysis, has been given in S1 and S3 Tables. A LOS gene was considered present if ≥80% of the query sequence was effectively mapped to the reference LOS gene sequence and had ≥80% nucleotide identity with the reference LOS gene sequence. Subsequently, based on the presence or absence of distinct LOS genes, combination of LOS genes or a LOS class was identified, and assigned to a particular *C*. *jejuni* or *C*. *coli* query sequence. Megablast detects a query by a single accession number for a complete genome sequence but incorporates multiple sub-accession numbers for a draft genome sequence. Therefore, to reduce the burden of query sequences, those draft sequence contigs were identified which contained the LOS biosynthesis gene sequence. For this purpose, all sequenced contigs associated with each draft sequence were aligned against the commonly present two LOS gene (*waaC* and *waaF*) sequences using Megablast. Subsequently, contigs with LOS sequence were used further for LOS classification analysis.

### Ethics statement

*Campylobacter* isolates from anonymised clinical samples were collected by the Swab method under the sterile conditions from already cultured plates. No experimentation was carried out on humans during this research and this research did not involve the use of human tissues, fluids or DNA samples.

## Supporting information

S1 Table LOS types of *C*. *jejuni* complete (n = 125) and draft sequences (n = 578). (PDF)

S2 Table Primers for the identification of *C*. *jejuni* LOS locus classes. (PDF)

S3 Table LOS types of *C*. *coli* complete (n = 22) and draft sequences (n = 542). (PDF)

S4 Table Summary of *C*. *coli* reference strains used to define LOS classes (Richards et al., 2013) in this study. (PDF)

S5 Table Summary of *C*. *jejuni* LOS locus typing and PCR products’ sequencing results. (PDF)
