# Supplementary material for: Molecular and in silico typing of the lipooligosaccharide biosynthesis gene cluster in Campylobacter jejuni and Campylobacter coli
Source: PLoS One. 2022 Mar 31;17(3):e0265585. doi: 10.1371/journal.pone.0265585 (PMC8970381; doi:10.1371/journal.pone.0265585)
Supplement: S1 Table — (PDF) [file pone.0265585.s001.pdf]

S1 Table. LOS types of *C. jejuni* complete (n=125) and draft sequences (n=578)

Complete sequences: LOS Group 1; Classes A1, A2, B1, B2, C & V

No.	*C. jejuni* Strain	Accession no.	51	17	14	15	5	6	7	8	9	5-II	10	11	Class	Host, Country
1	00-6200	NZ_CP010307.1					99/100	99/100	100/100	99/100	97/100		98/100	99/100	A2	Human, Canada
2	RM3196	NZ_CP012690.1					99/100	99/100	97/100	99/100	98/100		98/100	99/100	A1	Human, South Africa
3	RM3197	NZ_CP012689.1													A1	Human, South Africa
4	RM3420	NZ_CP017456.1					99/100	99/100	99/100	98/100	99/100		98/100	98/100	A1	Human, Canada
5	00-1597	NZ_CP010306.1					99/98	99/100	91/100	98/100	98/99		97/100	95/100	A2	Human, Canada
6	HF5-4A-4	NZ_CP007188.1					100/100	99/100	100/100	100/100	100/100		100/100	100/100	A1	Farm, UK
7	FDAARGOS_262	NZ_CP022076.1					99/100	96/100	96/100	98/100	98/99		98/100	97/100	A2	Bovine, US
8	TS1218	NZ_CP017860.1					99/100	99/100	99/100	98/100	99/99		97/100	98/99	A1	Chicken, US
9	32488	NC_021834.1					99/100	99/100	95/100	97/100	97/100		97/100	95/100	A2 WITH DOBLE Orf neuA1	Human, US
10	M129	NZ_CP007749.1					99/100	99/100	95/100	97/100	98/99		97/100	95/100	A2	Human, US
11	FDAARGOS_422	NZ_CP023867.1					99/100	99/100	97/100	99/100	98/100		98/100	99/100	A1	Human, US
12	MTVDSCj13	NZ_CP017032.1					98/100	99/100	99/100	98/100	98/100	99/100	98/100	98/100	B2	Chicken, US
13	RM3194	NZ_CP014344.1					98/100	99/100	99/100	98/100	98/100	99/100	98/100	98/100	B2	Human, South Africa
14	MTVDSCj16	NZ_CP017033.1					96/95				98/100	99/100		97/100	B1	Chicken, US
15	NCTC11351	NZ_LN831025.1					99/100	100/100	99/100	100/100	99/100	99/100	99/100	99/100	B1	Not Known
16	FORC_046	NZ_CP017229.1					98/100	100/100	99/100	98/100	98/99		98/100	99/100	B2	Human, South Korea
17	YH001	CP010058.1					98/100	98/100	93/100	99/100	98/100	99/100	98/100	99/100	B1	Beef, US
18	Cj3	NZ_KK365768.1					98/100	98/100	93/100	98/100	98/100	99/100	98/100	98/100	B1	Human, Thailand
19	FDAARGOS_265	NZ_CP022079.1						94/87	91/100	99/100	99/100	99/100	97/100	98/100	B1	Human, US
20	81-176_G1_B0	NZ_CP022440.1					99/100	99/100	95/100	98/100	99/100	99/100	100/100	99/100	B2	Human, UK
21	14980A	NZ_CP017029.1					96/95				98/100	99/100	98/100	97/100	B1	Turkey, US
22	11168-BN148	NC_018521.1			100/100	100/100		100/100	100/100	100/100	100/100		100/100	100/100	C	Unknown, Finland
23	00-2538	NC_022351.2			100/100	100/100		100/100	100/100	100/100	100/100		100/100	99/100	C	Human, Canada
24	00-2544	NC_022353.2			100/100	100/100		100/100	100/100	100/100	100/100		100/100	99/100	C	Human, Canada
25	00-2426	NC_022352.2			100/100	100/100		100/100	100/100	100/100	100/100		100/100	99/100	C	Human, Canada
26	00-2425	NC_022362.2			100/100	100/100		100/100	100/100	100/100	100/100		100/100	99/100	C	Human, Canada
27	NCTC 11168-K12E5	NZ_CP006685.1			100/100	100/100		99/100	100/100	100/100	100/100		100/100	99/100	C	Human, Canada
28	NCTC 11168-Kf1	NZ_CP006686.1			100/100	100/100		99/100	100/100	100/100	100/100		100/100	99/100	C	Human, Canada
29	D42a	CP007751.1			100/100	100/100		100/100	100/100	100/100	100/100		100/100	99/100	C	Chicken, US
30	NCTC 11168-mcK12E5	NZ_CP006688.1			100/100	100/100		99/100	100/100	100/100	100/100		100/100	99/100	C	Human, Canada
31	NCTC 11168-GSv	NZ_CP006689.1			100/100	100/100		100/100	100/100	100/100	100/100		100/100	99/100	C	Human, Canada
32	00-0949	NZ_CP010301.1			100/100	99/100		100/100	100/100	100/100	100/100		99/100	99/100	C	Human, Canada
33	WP2202	NZ_CP014742.1			100/100	100/100		99/100	100/100	100/100	100/100		99/100	99/100	C	Chicken, US
34	ZP3204	NZ_CP017856.1			100/100	100/100		99/100	100/100	100/100	100/100		99/100	99/100	C	Chicken, US
35	NS4-5-1	NZ_CP007192.1			100/100	99/100		99/100	100/100	99/100	100/100		100/100	99/100	C	Farm, UK
36	NS4-9-1	NZ_CP007193.1			100/100	99/100		100/100	100/100	99/100	100/100		100/100	99/100	C	Farm, UK
37	CFSAN032806	NZ_CP023543.1			99/100	99/100		99/100	100/100	100/100	100/100		99/100	99/100	C	Chicken, US
38	01-1512	NZ_CP010072.1			100/100	99/100		100/100	100/100	100/100	100/100		99/100	99/100	C	Human, Canada
39	RM1285	NZ_CP012696.1			100/100	100/100		99/100	100/100	100/100	100/100		99/100	99/100	C	Chicken, US
40	FDAARGOS_263	NZ_CP022077.1			100/100	100/100		100/100	100/100	100/100	100/100		100/100	100/100	C	Human, US
41	YQ2210	NZ_CP017859.1			100/100	100/100		99/100	100/100	100/100	100/100		99/100	99/100	C	Turkey, US
42	11168H/lacY	NZ_CP022439.1			100/100	100/100		100/100	100/100	100/100	100/100		100/100	100/100	C	Human, UK
43	MTVDSCj07	NZ_CP017031.1			100/100	100/100		99/100	100/100	100/100	100/100		99/100	99/100	C	Chicken, US
44	YH002	NZ_CP020776.1			100/100	100/100		99/100	100/100	100/100	100/100		99/100	99/100	C	Calf, US
45	NS4-1-1	NZ_CP007191.1			100/100	99/100		100/100	100/100	99/100	100/100		100/100	99/100	C	Farm, UK
46	11168H/araE	NZ_CP022559.1			100/100	100/100		100/100	100/100	100/100	100/100		100/100	99/100	C	Human, UK
47	NCTC 12664	NZ_CP028912.1			100/100	100/100		100/100	100/100	100/100	100/100		99/100	99/100	C	Chicken, UK
48	IA3902	NC_017279.1			100/100	99/100		100/100	100/100	100/100	100/100		99/100	99/100	C	Sheep, US
49	12567	NZ_CP028909.1			100/100	99/100		99/100	100/100	100/100	100/100		100/100	99/100	C	Chicken, UK

50	NCTC 12660	NZ_CP028910.1			100/100	99/100		99/100	100/100	100/100	100/100		100/100	99/100	C	Chicken, UK
51	OD267	NZ_CP014744.1			100/100			99/91	100/100	100/100	100/100		99/100	99/100	V	Chicken, US
52	PT14	NC_018709.4			100/100	100/100		99/100	100/100	100/100	100/100		99/100	99/100	C WITH ORF 48L INSERTION	Unknown, UK

Complete sequences: LOS Group 2; Classes P, H, E, O & W

No.	*C. jejuni* Strain	Accession no.	21-25	26	26'	27	28	29-34	Class	Host, Country
1	CG8421	NZ_CP005388.1	98/98		99/100	99/92	99/100	98/99	P	Human, US
2	4031	NC_022529.1	98/98		100/100	99/92	99/100	99/99	P	Unknown, Finland
3	MTVDSCj20	NZ_CP008787.1	98/100	99/100		100/100		100/100	O	Chicken, US
4	IF1100	NZ_CP017863.1	98/98		99/100	98/100		99/100	H	Chicken, US
5	RM1246-ERRC	NZ_CP022470.1	98/98		100/100	99/100		99/100	H	Human, US
6	FDAARGOS_266	NZ_CP022080.1	96/100	99/100		98/92	99/100	98/99	E	Unknown, US
7	CJM1cam	NZ_CP012149.1	99/85	99/100		99/99		98/100	W	Human, UK
8	CJ677CC519	NZ_CP010471.1	98/100	99/100		98/100		98/100	O	Faeces, Finland
9	CJ677CC002	NZ_CP010472.1	98/100	99/100		98/100		98/100	O	Human, Finland
10	CJ677CC534	NZ_CP010473.1	98/100	99/100		98/100		98/100	O	Human, Finland
11	CJ677CC536	NZ_CP010474.1	98/100	99/100		98/100		98/100	O	Human, Finland
12	CJ677CC073	NZ_CP010475.1	98/100	99/100		98/100		98/100	O	Human, Finland
13	CJ677CC521	NZ_CP010476.1	98/100	99/100		98/100		98/100	O	Human, Finland
14	CJ677CC526	NZ_CP010477.1	98/100	99/100		98/100		98/100	O	Human, Finland
15	CJ677CC036	NZ_CP010479.1	98/100	99/100		98/100		98/100	O	Human, Finland
16	CJ677CC524	NZ_CP010480.1	98/100	99/100		98/100		98/100	O	Human, Finland
17	CJ677CC016	NZ_CP010481.1	98/100	99/100		98/100		98/100	O	Human, Finland
18	CJ677CC041	NZ_CP010482.1	98/100	99/100		98/100		98/100	O	Human, Finland
19	CJ677CC535	NZ_CP010483.1	98/100	99/100		98/100		98/100	O	Human, Finland
20	CJ677CC092	NZ_CP010488.1	98/100	99/100		98/100		98/100	O	Human, Finland
21	CJ677CC530	NZ_CP010489.1	98/100	99/100		98/100		98/100	O	Human, Finland
22	CJ677CC532	NZ_CP010490.1	98/100	99/100		98/100		98/100	O	Human, Finland
23	CJ677CC529	NZ_CP010491.1	98/100	99/100		98/100		98/100	O	Human, Finland
24	CJ677CC531	NZ_CP010492.1	98/100	99/100		98/100		98/100	O	Human, Finland
25	CJ677CC062	NZ_CP010493.1	98/100	99/100		98/100		98/100	O	Human, Finland
26	CJ677CC059	NZ_CP010494.1	98/100	99/100		98/100		98/100	O	Human, Finland
27	CJ677CC032	NZ_CP010496.1	98/100	99/100		98/100		98/100	O	Human, Finland
28	CJ677CC033	NZ_CP010497.1	98/100	99/100		98/100		98/100	O	Human, Finland
29	CJ677CC537	NZ_CP010498.1	98/100	99/100		98/100		98/100	O	Human, Finland
30	CJ677CC542	NZ_CP010499.1	98/100	99/100		98/100		98/100	O	Human, Finland
31	CJ677CC528	NZ_CP010500.1	98/100	99/100		98/100		98/100	O	Human, Finland
32	CJ677CC538	NZ_CP010495.1	98/100	99/100		98/100		98/100	O	Human, Finland
33	CJ677CC520	NZ_CP010501.1	98/100	99/100		98/100		98/100	O	Human, Finland
34	CJ677CC014	NZ_CP010502.1	98/100	99/100		98/100		98/100	O	Human, Finland
35	CJ677CC039	NZ_CP010503.1	98/100	99/100		98/100		98/100	O	Human, Finland
36	CJ677CC085	NZ_CP010504.1	98/100	99/100		98/100		98/100	O	Human, Finland
37	CJ677CC052	NZ_CP010505.1	98/100	99/100		98/100		98/100	O	Human, Finland
38	CJ677CC527	NZ_CP010506.1	98/100	99/100		98/100		98/100	O	Human, Finland
39	CJ677CC078	NZ_CP010507.1	98/100	99/100		98/100		98/100	O	Human, Finland
40	CJ677CC523	NZ_CP010508.1	98/100	99/100		98/100		98/100	O	Human, Finland
41	CJ677CC540	NZ_CP010509.1	98/100	99/100		98/100		98/100	O	Human, Finland
42	CJ677CC040	NZ_CP010510.1	98/100	99/100		98/100		98/100	O	Human, Finland
43	CJ677CC061	NZ_CP010511.1	98/100	99/100		98/100		98/100	O	Human, Finland
44	CJ677CC539	NZ_CP010457.1	98/100	99/100		98/100		98/100	O	Human, Finland
45	CJ677CC533	NZ_CP010458.1	98/100	99/100		98/100		98/100	O	Human, Finland
46	CJ677CC047	NZ_CP010459.1	98/100	99/100		98/100		98/100	O	Human, Finland
47	CJ677CC058	NZ_CP010460.1	98/100	99/100		98/100		98/100	O	Human, Finland
48	CJ677CC013	NZ_CP010461.1	98/100	99/100		98/100		98/100	O	Human, Finland
49	CJ677CC100	NZ_CP010462.1	98/100	99/100		98/100		98/100	O	Human, Finland
50	CJ677CC522	NZ_CP010463.1	98/100	99/100		98/100		98/100	O	Human, Finland
51	CJ677CC094	NZ_CP010464.1	98/100	99/100		98/100		98/100	O	Human, Finland
52	CJ677CC008	NZ_CP010465.1	98/100	99/100		98/100		98/100	O	Human, Finland
53	CJ677CC541	NZ_CP010466.1	98/100	99/100		98/100		98/100	O	Human, Finland
54	CJ677CC024	NZ_CP010467.1	98/100	99/100		98/100		98/100	O	Human, Finland
55	CJ677CC064	NZ_CP010468.1	98/100	99/100		98/100		98/100	O	Human, Finland
56	CJ677CC525	NZ_CP010469.1	98/100	99/100		98/100		98/100	O	Human, Finland
57	CJ677CC026	NZ_CP010470.1	98/100	99/100		98/100		98/100	O	Human, Finland

58	CJ677CC034	NZ_CP010484.1	98/100	99/100		98/100		98/100	O	Human, Finland
59	CJ677CC086	NZ_CP010485.1	98/100	99/100		98/100		98/100	O	Human, Finland
60	CJ677CC095	NZ_CP010486.1	98/100	99/100		98/100		98/100	O	Human, Finland
61	CJ677CC012	NZ_CP010487.1	98/100	99/100		98/100		98/100	O	Human, Finland
62	CJ677CC010	CP010478.1	98/98	99/100		98/100		98/99	O	Human, Finland

Complete sequences: LOS Group 3; Classes D, F & K

No.	*C. jejuni* Strain	Accession no.	17	18	19	20		38	40	41	42-45	46	49-50	16	Class	Host, Country
1	R14	CP005081.1	99/100	99/100	100/100	100/100								100/100	D	Unknown, UK
2	S3	NC_017281.1	99/100	98/94	99/100	99/100									F	Unknown, US
3	ATCC 35925	NZ_CP020045.1	99/100	96/94	98/100	97/100									F	Pigeon, Sweden
4	35925	NZ_CP010906.1	99/100	96/94	98/100	97/100									F	Human, Sweden
5	FJ3124	NZ_CP017862.1	99/100		96/91										F	Chicken, US
6	NCTC12662	NZ_CP019965.1	99/100	96/94	98/100	97/100									F	Unknown, UK
7	NCTC 12661	NZ_CP028911.1	99/100	96/94	98/100	97/100									F	Avian, UK
8	FDAARGOS_421	NZ_CP023866.1	99/100	98/94	99/100	99/100									F	Chicken, US
9	F38011	NZ_CP006851.1	99/100	94/100	95/98								99/100	96/100	K	Unknown, US
10	HF5-5-1	NZ_CP007189.1	99/100	93/100	95/98								99/100	96/100	K	Farm, UK
11	HF5-7-1	NZ_CP007190.1	99/100	93/100	95/98								99/100	96/100	K	Farm, UK

Draft sequences: LOS Group 1; Classes A1, A2, B1, B2, C, V, M & R

Class A1

No.	*C. jejuni* Strain	Accession no.	Contig no.	51	17	14	15	5	6	7	8	9	5-II	10	11	Class	Host, Country
1	ICDCCJ07002	APNP01000000	2					99/100	99/100	97/100	99/100	98/100		98/100	99/100	A1	Human, China
2	HN-CJD07035	ARYE01000000	6					99/100	99/100	97/100	99/100	98/100		98/100	99/100	A1	Human, China
3	BJ-CJD101	ARWV01000000	8					99/100	99/100	99/100	98/100	99/100		98/100	98/100	A1	Human, China
4	CVM 41974	JAKS01000000	4,8					99/100	99/100	99/100	98/100	99/100		98/100	98/100	A1	Human, US
5	HB-CJGB-QYT	ATBM01000000	3					99/100	99/100	97/100	99/100	98/100		98/100	99/100	A1	Human, China
6	BJ-CJGB96G25	ASXL01000000	3					99/100	99/100	99/100	98/100	99/100		98/100	98/100	A1	Human, China
7	BJ-CJGB95377	ASXK01000000	2					99/100	99/100	99/100	98/100	99/100		98/100	98/100	A1	Human, China
8	BJ-CJGB96114	ASXM01000000	2					99/100	99/100	99/100	98/100	99/100		98/100	98/100	A1	Human, China
9	BJ-CJGB96299	ASXN01000000	3					99/100	99/100	97/100	99/100	98/100		98/100	99/100	A1	Human, China
10	CF93-6	AANJ01000000	7					99/100	99/100	97/100	99/100	98/100		98/100	99/100	A1	Unknown
11	LMG 23216	AIOA01000000	5					100/100	99/100	99/100	98/100	99/100		98/97		A1	Chicken, Belgium
12	OXC6626	CUVM01000000	1					99/100	99/100	99/100	98/100	99/100		98/100	98/100	A1	Faeces, UK
13	OXC6414	CUNR01000000	1					99/100	99/100	99/100	98/100	99/100		98/100	98/100	A1	Faeces, UK
14	OXC6408	CUNK01000000	1					99/100	95/99	97/100	98/100	99/100		98/100	99/100	A1	Faeces, UK
15	OXC6305	CUJN01000000	1					100/100	100/100	100/100	100/100	100/100		100/100	100/100	A1	Faeces, UK
16	OXC6301	CUJJ01000000	1					100/100	100/100	100/100	100/100	100/100		100/100	100/100	A1	Faeces, UK
17	OXC6406	CUNI01000000	1					100/100	99/100	99/100	98/100	99/100		98/100	98/100	A1	Faeces, UK
18	OXC6535	CUSF01000000	1					100/100	99/100	99/100	98/100	99/100		98/100	98/100	A1	Faeces, UK
19	OXC6397	CUNA01000000	1					100/100	100/100	100/100	100/100	100/100		100/100	100/100	A1	Faeces, UK
20	OXC6534	CUSG01000000	1					100/100	100/100	100/100	100/100	99/100		100/100	100/100	A1	Faeces, UK
21	OXC6641	CUWB01000000	1					99/100	99/100	99/100	98/100	99/100		98/100	98/100	A1	Faeces, UK
22	OXC6494	CUQN01000000	2					100/100	99/100	100/100	100/100	100/100		100/100	100/100	A1	Faeces, UK
23	OXC6264	CUHV01000000	2					99/100	99/100	99/100	98/100	99/100		98/100	98/100	A1	Faeces, UK
24	OXC6482	CUPZ01000000	2					99/100	95/99	97/100	97/100	97/100		98/97		A1	Faeces, UK
25	OXC6273	CUIF01000000	1					100/100	100/100	100/100	98/100	99/100		99/100	98/100	A1	Faeces, UK
26	OXC6479	CUPX01000000	1					99/100	99/100	97/100	99/100	98/100		98/100	99/100	A1	Faeces, UK
27	Faeces	CZJF01000000	12					100/100	100/100	100/100	99/100	99/100		100/100	100/100	A1	Chicken, Spain
28	Neck Skin	CZJM01000000	2						100/100	100/100	99/100	100/100		100/100	100/100	A1	Chicken, Spain
29	Faeces	CZHY01000000	12					99/100	99/100	97/100	99/100	98/100		98/100	99/100	A1	Chicken, Spain
30	BCW_6893	MJYX01000000	16					98/100	96/100	96/100		97/100		97/97		A1	Crow, US
31	BCW_3804	MKAL01000000	41					100/100	100/100	100/100	98/100	99/100		99/100	98/100	A1	Crow, US
32	BCW_4328	MKAN01000000	3					98/100	96/100	96/100	96/100	97/100		97/97		A1	Crow, US
33	CDPHFDLB-F12M00560	MOVU01000000	1					99/100	99/100	99/100	98/100	99/100		98/100	98/100	A1	Cow, US
34	CDPHFDLB-M00214	MOVP01000000	1					99/100	99/100	99/100	98/100	99/100		98/100	98/100	A1	Cow, US
35	CDPHFDLB-M00224	MOVO01000000	9					99/100	99/100	99/100	98/100	99/100		98/100	98/100	A1	Cow, US
36	CDPHFDLB-F12M00566-a2	MOVL01000000	7					99/100	99/100	99/100	98/100	99/100		98/100	98/100	A1	Cow, US
37	BCW_6884	MKEW01000000	59					98/100	96/100	96/100	96/100	97/100		97/97		A1	Crow, US

38	BCW_4324	MKET01000000	30					99/100	99/100	98/100	97/100	97/100		96/97		A1	Crow, US
39	CJ096CC21	MLDN01000000	6					99/100	99/100	97/100	99/100	98/100		98/100	99/100	A1	Human, Finland
40	W20	NFNM01000000	65					99/100	95/100	96/100	97/100	98/100		97/100	99/100	A1	Environment, Canada
41	W16	NFNR01000000	35					99/100	95/100	96/100	97/100	98/100		97/100	99/100	A1	Environment, Canada
42	Po_2	CCDE01000000	46					99/100	99/100	99/100	98/100	99/100		98/100	98/100	A1	Unknown, Finland
43	Ma_B	CCDD01000000	55					99/100	99/100	99/100	98/100	99/100		98/100	98/100	A1	Unknown, Finland
44	Le_204R	CCDB01000000	45					99/100	99/100	99/100	98/100	99/100		98/100	98/100	A1	Unknown, Finland
45	Ma_1	CCCZ01000000	47					99/100	99/100	99/100	98/100	99/100		98/100	98/100	A1	Unknown, Finland
46	Le_755	CCDC01000000	21, 52					99/100	99/100	99/100	98/100	99/100		98/100	98/100	A1	Unknown, Finland
47	Po_1	CCDA01000000	68					99/100	99/98	99/100	98/100	99/100		98/100	98/100	A1	Unknown, Finland
48	P10-2209	JYEC01000000	5					99/100	95/99	97/100	97/100	97/100		98/97		A1	Pigeon, Japan
49	P3-2209	JYEA01000000	1					99/100	95/99	97/100	97/100	97/100		98/97		A1	Pigeon, Japan
50	P5-2209	JYEB01000000	6					99/100	95/99	97/100	97/100	97/100		98/97		A1	Pigeon, Japan
51	OXC6521	CURR01000000	1, 16					100/100	100/100	100/100	99/100	100/100		100/100	100/100	A1	Faeces, UK
52	OXC6525	CURV01000000	1					100/100	100/100	100/100	100/100	99/100		100/100	100/100	A1	Faeces, UK
53	BCW_3797	MJVM01000000	31					100/100	100/100	100/100	98/100	99/100		99/100	98/100	A1	Crow, US
54	BCW_3799	MJVO01000000	9					98/100	96/100	96/100	96/100	97/100		97/97		A1	Crow, US
55	ICDCCJ07001	CP002029.1	-					99/100	99/100	97/100	99/100	98/100		98/100	99/100	A1	Unknown, China
56	Faeces	CZIN01000000	58, 64					99/100	99/100	99/100	99/100	99/100		100/100	100/100	A1	Chicken, Spain
57	BCW_3797	MJVM01000000	31					100/100	100/100	100/100	98/100	99/100		99/100	98/100	A1	Crow, US
58	OXC6525	CURV01000000	1					100/100	100/100	100/100	100/100	99/100		100/100	100/100	A1	Faeces, UK
59	CVM N15870	JOVW01000000	2					99/100	95/100	96/100	97/100	98/100		98/100		A1	Turkey, US
60	Water	CZJO01000000	32					100/100	100/100	100/100	100/100	99/100		100/100	100/100	A1	Environment, Spain
61	Meat	CZJI01000000	43					100/100	100/100	100/100	100/100	100/100		100/100	100/100	A1	Chicken, Spain
62	CDPHFDLB-F12M00560	MOVT01000000	8					99/100	99/100	99/100	98/100	99/100		98/100	98/100	A1	Cow, US

Class A2

No.	*C. jejuni* Strain	Accession no.	Contig no.	51	17	14	15	5	6	7	8	9	5-II	10	11	Class	Host, Country
1	60004	AIOE01000000	20					100/100	100/100	100/100	100/100	100/100		99/100	95/100	A2	Chicken, US
2	86605	AIOJ01000000	4					99/100	98/99	95/100	98/100	97/100		99/100	95/100	A2	Chicken, US
3	2008-872	AIOR01000000	5, 31					99/100	99/100	100/100	99/100	97/100		98/100	99/100	A2	Human, France
4	1997-7	AIOX01000000	1					99/100	99/100	100/100	99/100	97/100		98/100	98/100	A2	Human, US
5	87459	AIPE01000000	35					99/100	98/99	99/100	98/100	99/100		99/100	95/100	A2	Chicken, US
6	1798	AIPI01000000	21, 36					99/100	99/100	95/100	99/100	97/100		98/100	98/100	A2	Cow, US
7	OXC6453	CURA01000000	1					99/100	99/100	100/100	99/100	97/100		98/100	99/100	A2	Faeces, UK
8	OXC6306	CUJO01000000	1					99/100	99/100	100/100	99/100	97/100		98/100	99/100	A2	Faeces, UK
9	OXC6579	CUTT01000000	1					99/100	99/100	100/100	99/100	97/100		98/100	99/100	A2	Faeces, UK
10	OXC6346	CULG01000000	1					99/100	99/100	100/100	99/100	97/100		98/100	99/100	A2	Faeces, UK
11	OXC6490	CUQJ01000000	1					99/100	99/100	100/100	99/100	97/100		98/100	99/100	A2	Faeces, UK
12	OXC6468	CUPL01000000	1					99/100	99/100	96/100	98/100	98/100		97/100	95/100	A2	Faeces, UK
13	OXC6402	CUNF01000000	1					99/100	99/100	100/100	99/100	97/100		98/100	99/100	A2	Faeces, UK
14	OXC6570	CUTJ01000000	1					99/100	99/100	100/100	99/100	97/100		98/100	98/100	A2	Faeces, UK
15	OXC6283	CUIN01000000	1					99/100	99/100	100/100	99/100	97/100		98/100	99/100	A2	Faeces, UK
16	OXC6268	CUHZ01000000	1					99/100	99/100	100/100	99/100	97/100		98/100	99/100	A2	Faeces, UK
17	OXC6505	CUQZ01000000	1					99/100	98/100	95/100	97/100	98/100		97/100	95/100	A2	Faeces, UK
18	OXC6340	CULA01000000	1					99/98	99/100	91/100	98/100	98/100		98/100	96/100	A2	Faeces, UK
19	Water	CZJL01000000	12					99/100	99/100	100/100	99/100	97/100		98/100	98/100	A2	Environment, Spain
20	CVM 41900	JAKC01000000	4					99/98	99/100	91/100	98/100	98/100		98/100	96/100	A2	Human, US
21	CVM N534	JOUW01000000	7					99/98	99/100	91/100	98/100	97/99		97/100	95/100	A2	Chicken, US
22	CCN25	FBJN01000000	28					99/98	99/100	91/100	98/100	98/100		98/100	96/100	A2	Poultry Environment, UK
23	CVM N9016	JOVD01000000	12					99/100	99/100	93/100	98/100	97/99		98/100	97/100	A2	Chicken, US
24	CVM N9095	JOVH01000000	4					99/98	99/100	99/100	98/100	98/100		97/100	95/100	A2	Chicken, US
25	CDPHFDLB-F15M03173	MOTS01000000	8					99/100	99/100	100/100	98/100	98/100		98/100	98/100	A2	Cow, US
26	CDPHFDLB-F15M03174	MPBJ01000000	2					99/100	99/100	100/100	98/100	98/100		98/100	98/100	A2	Milk, US
27	BCW_5913	MKES01000000	74					99/100	98/100	91/100	98/100	97/100		98/100	99/100	A2	Monkey, US
28	W33	NFNA01000000	92					99/100	98/100	98/100	98/100	97/100		98/100	99/100	A2	Environmental water, Canada
29	W11	NFNW01000000	15					99/100	99/100	100/100	99/100	97/100		98/100	98/100	A2	Environmental water, Canada
30	W10	NFNX01000000	25, 45					99/100	99/100	100/100	99/100	97/100		98/100	99/100	A2	Environmental water, Canada
31	S1	NFOH01000000	16					99/100	98/100	98/100	98/100	97/100		98/100	99/100	A2	Environmental water, Canada
32	H34	NFOO01000000	25, 189					99/100	99/100	100/100	99/100	97/100		98/100	99/87	A2	Human, Canada
33	H22	NFPB01000000	41, 67					99/100	99/100	100/100	99/100	97/100		98/100	99/87	A2	Human, Canada
34	H17	NFPG01000000	40, 42					99/98	99/100		98/100	98/100		98/100	96/100	A2	Human, Canada
35	B2	NFQF01000000	41					99/100	99/100	100/100	99/100	97/100		98/100	98/100	A2	Cow, Canada
36	OXC6589	CUUD01000000	1, 14					99/100	99/100	100/100	99/100	97/100		98/100	99/100	A2	Faeces, UK
37	BCW_4727	MKAZ01000000	10					99/100	99/100	100/100	99/100	97/100		98/100	99/100	A2	Unknown, US

38	CDPHFDL-F15M03174-C2	MOTQ01000000	2					99/100	99/100	100/100	98/100	98/100		98/100	98/100	A2	Cow Milk, US
39	CVM N1630	JOUH01000000	20					99/100	99/100	93/100	98/100	98/100		98/100	96/100	A2	Chicken, US

Class B1

No.	*C. jejuni* Strain	Accession no.	Contig no.	51	17	14	15	5	6	7	8	9	5-II	10	11	Class	Host, Country
1	D2600	AGTF01000000	47					97/86	96/83	91/100	99/100	99/100	99/100	98/100	98/100	B1	Human, US
2	Cj5	AUUK01000000	29					98/100	94/87	93/100	98/100	98/100	99/100	98/100	98/100	B1	Human, Thailand
3	Cj2	AUUM01000000	34					98/100	97/83	94/100	99/100	98/100	99/100	99/100	99/100	B1	Human, Thailand
4	P110B	AEIO01000000	2, 3					98/100		93/81			99/36	98/100	99/100	B1	Chicken, New Zealand
5	LMG 23210	AIPN01000000	30. 48					98/100	94/87	93/100	98/100	98/100	99/100	98/100	98/100	B1	Chicken, Belgium
6	30286	AUUH01000000	4					98/100	94/87	93/100	98/100	98/100	99/100	98/100	98/100	B1	Human, Viet Nam
7	OXC6640	CUWA01000000	2					98/100	94/87	93/100	98/100	98/100	99/100	98/100	98/100	B1	Faeces, UK
8	OXC6554	CUSQ01000000	2					98/100	96/83	93/100	98/100	98/100	99/100	98/100	98/100	B1	Faeces, UK
9	OXC6509	CURF01000000	1					97/86	96/83	91/100	98/100	98/100	99/100	98/100	98/100	B1	Faeces, UK
10	OXC6341	CULB01000000	2					98/100	96/83	93/100	98/100	98/100	99/100	98/100	98/100	B1	Faeces, UK
11	OXC6304	CUJM01000000	1					98/100	94/87	93/100	98/100	98/100	99/100	98/100	98/100	B1	Faeces, UK
12	OXC6262	CUHT01000000	2					98/100	96/83	93/100	98/100	98/100	99/100	98/100	98/100	B1	Faeces, UK
13	OXC6484	CUQC01000000	2					99/100	99/100		99/100	99/100	99/100	99/100	99/100	B1	Faeces, UK
14	OXC6572	CUTL01000000	1					98/100	96/83	93/100	98/100	98/100	99/100	98/100	98/100	B1	Faeces, UK
15	OXC6618	CUVD01000000	1					98/100	94/87	91/100	98/100	98/100	99/100	99/100	99/100	B1	Faeces, UK
16	OXC6621	CUVH01000000	1					98/100	96/83	93/100	98/100	98/100	99/100	98/100	98/100	B1	Faeces, UK
17	OXC6359	CULL01000000	2					98/100	94/87	93/100	98/100	98/100	99/100	98/100	98/100	B1	Faeces, UK
18	OXC6475	CUPS01000000	1					98/100	96/83	93/100	98/100	98/100	99/100	98/100	98/100	B1	Faeces, UK
19	OXC6560	CUSY01000000	2					98/100	94/87	93/100	98/100	98/100	99/100	98/100	98/100	B1	Faeces, UK
20	OXC6352	CUNL01000000	2					98/100	96/83	93/100	98/100	98/100	99/100	98/100	98/100	B1	Faeces, UK
21	OXC6528	CURZ01000000	1					97/86	95/83	93/100	98/100	98/100	99/100	98/100	98/100	B1	Faeces, UK
22	OXC6584	CUTZ01000000	1					97/86	96/83	91/100	98/100	98/100	99/100	98/100	98/100	B1	Faeces, UK
23	OXC6288	CUIT01000000	2					98/100	94/87	93/100	98/100	98/100	99/100	98/100	98/100	B1	Faeces, UK
24	OXC6541	CUSN01000000	2					98/100	96/83	93/100	98/100	98/100	99/100	98/100	98/100	B1	Faeces, UK
25	OXC6544	CUSS01000000	1					97/86	96/83	91/100	99/100	98/100	99/100	99/100	99/100	B1	Faeces, UK
26	OXC6392	CUMV01000000	1					98/100	96/83	93/100	98/100	98/100	99/100	98/100	98/100	B1	Faeces, UK
27	OXC6581	CUTV01000000	2					98/100	94/87	93/100	98/100	98/100	99/100	98/100	98/100	B1	Faeces, UK
28	OXC6323	CUKJ01000000	1					98/100	94/87	93/100	98/100	98/100	99/100	98/100	98/100	B1	Faeces, UK
29	OXC6361	CULN01000000	1					98/100	94/87	93/100	98/100	98/100	99/100	98/100	98/100	B1	Faeces, UK
30	OXC6485	CUQD01000000	2					99/100	99/100	99/100	99/100	99/100	99/100	99/100	99/100	B1	Faeces, UK
31	OXC6349	CUME01000000	2					99/100	99/100	99/100	99/100	99/100	100/100	100/100	100/100	B1	Faeces, UK
32	OXC6455	CURX01000000	1					98/100	96/83	91/100	99/100	99/100	99/100	98/100	98/100	B1	Faeces, UK
33	Faeces	CZIA01000000	46					98/100	96/83	93/100	98/100	98/100	99/100	98/100	98/100	B1	Chicken, Spain
34	Neck	CZIG01000000	19					97/86	96/83	91/100	99/100	99/100	99/100	98/100	98/100	B1	Chicken, Spain
35	CVM N9016	JOVD01000000	12					98/100	97/83	94/100	99/100	99/100	99/100	100/100	100/100	B1	Chicken, US
36	CVM N9095	JOVH01000000	4					97/86	94/87	95/100	99/100	98/100	99/100	97/100	98/100	B1	Chicken, US
37	CVM N534	JOUW01000000	7					97/86	94/87	91/100	99/100	99/100	99/100	98/100	97/100	B1	Chicken, US
38	BCW_6910	MJZF01000000	43					99/100	99/100	99/100	99/100	99/100	99/100	100/100	100/100	B1	Faeces, US
39	BCW_6956	MJZQ01000000	25					99/100	99/100	99/100	99/100	99/100	99/100	100/100	100/100	B1	Faeces, US
40	BCW_5122	MKBQ01000000	34						100/22	95/100	98/100	98/100	99/100	98/100	98/100	B1	Human, US
41	BCW_5129	MKBW01000000	47					98/100	94/87	93/100	98/100	98/100	99/100	98/100	98/100	B1	Human, US
42	BCW_5135	MKCA01000000	47					98/100	94/87	93/100	98/100	98/100	99/100	98/100	98/100	B1	Human, US
43	BCW_5136	MKCB01000000	17					98/100	94/87	93/100	98/100	98/100	99/100	98/100	98/100	B1	Human, US
44	BCW_5144	MKCG01000000	48					98/100	96/83	93/100	98/100	98/100	99/100	98/100	98/100	B1	Human, US
45	BCW_5148	MKCK01000000	36					98/100	96/83	93/100	98/100	98/100	99/100	98/100	98/100	B1	Human, US
46	BCW_5151	MKCM01000000	53					98/100	94/87	93/100	98/100	98/100	99/100	98/100	98/100	B1	Human, US
47	CDPHFDLB-F15M00516E1	MOUW01000000	2					99/100	99/100	100/100	99/100	99/100	100/100	100/100	100/36	B1	Environment, US
48	BCW_5154	MKEZ01000000	31					98/100	96/83	93/100	98/100	98/100	99/100	98/100	98/100	B1	Human, US
49	CAM970	BDRZ01000000	7					98/100	94/87	93/100	98/100	98/100	99/100	98/100	98/100	B1	Human, Japan
50	W46	NFMN01000000	13					99/100	99/100	100/100	99/100	99/100	100/64			B1	Environmental water, Canada
51	Isolate_1	NFQL01000000	23, 111					97/86	96/83	92/55			99/37	98/100	97/100	B1	Retail Chicken, canada
52	OXC6391	CUMW01000000	1, 2					98/100	94/87	93/100	98/100	98/100	99/100	98/100	98/100	B1	Faeces, UK
53	OXC6574	CUTO01000000	1					98/100	96/83	93/100	98/100	98/100	99/100	98/100	98/100	B1	Faeces, UK
54	OXC6466	CUPJ01000000	2					98/100	94/87	93/100	98/100	98/100	99/100	98/100	98/100	B1	Faeces, UK
55	OXC6550	CUUZ01000000	1					98/100	96/83	93/100	98/100	98/100	99/100	98/100	98/100	B1	Faeces, UK
56	OXC6413	CUNQ01000000	1					97/86	94/87	91/100	99/100	99/100	99/100	97/100	98/100	B1	Faeces, UK

57	OXC6635	CUVW01000000	2					98/100	94/87	93/100	98/100	98/100	99/100	98100	98/100	B1	Faeces, UK
58	OXC6458	CUPA01000000	1					98/100	96/83	93/100	98/100	98/100	99/100	98/100	98/100	B1	Faeces, UK
59	OXC6555	CUST01000000	1					98/100	96/83	93/100	98/100	98/100	99/100	98/100	98/100	B1	Faeces, UK
60	OXC6501	CUQU01000000	1					98/100	91/81	93/100	99/100	98/100	99/100	98/100	99/100	B1	Faeces, UK
61	BJ-CJD120	LISM01000000	1					98/100	94/87	93/100	98/100	98/100	99/100	98/100	98/100	B1	Human, China
62	BCW_5121	MKBP01000000	1					98/100	94/87	93/100	98/100	99/100	100/100	99/100	99/100	B1	Human, US
63	BCW_5146	MKCI01000000	18					98/100	96/83	93/100	98/100	98/100	99/100	98/100	98/100	B1	Human, US
64	BCW_5159	MKFE01000000	38					98/100	94/87	93/100	98/100	98/100	99/100	98/100	98/100	B1	Human, US
65	FDAARGOS_264	NBTT01000000	2					97/86	96/83	91/100	99/100	99/100	99/100	97/100	98/100	B1	Human, US
66	CVM 41922	JAKL01000000	27					98/100	91/81	93/100	99/100	98/100	99/100	98/100	99/100	B1	Human, US
67	MEAT	CZJD01000000	34					98/100	91/81	93/100	99/100	98/98	99/100	98/100	99/100	B1	Chicken, Spain
68	BCW_4735	MKBD01000000	1					98/100	91/81	93/100	99/100	98/100	99/100	98/100	99/100	B1	Unknown, US
69	BCW_4755	MKBN01000000	24					98/100	89/85	93/100	99/100	98/100	99/100	98/100		B1	Unknown, US
70	Neck Skin	CZIZ01000000	23					98/100	89/85	93/100	99/100	98/100	99/100	98/100	99/100	B1	Chicken, Spain
71	OXC6477	CUPV01000000	4					98/100	89/85	93/100	99/100	98/100	99/100	98/100		B1	Faeces, UK
72	OXC6430	CUOK01000000	1					98/100	89/85	93/100	99/100	98/100	99/100	98/100	99/100	B1	Faeces, UK
73	OXC6454	CURN01000000	5					98/100	91/81	93/100	99/100	98/100	99/100	98/100	99/100	B1	Faeces, UK
74	OXC6295	CUJC01000000	1, 54					98/100	89/85	93/100	99/100	98/100	99/100	98/100	99/100	B1	Faeces, UK
75	H25	NFOY01000000	106					98/100	91/81	93/100	99/100	98/100	99/100	98/100	99/100	B1	Human, Canada
76	C12	NFQA01000000	59, 69					98/100	91/81	93/100	99/100	98/100	99/100	98/100	99/99	B1	Faeces, Canada
77	BCW_4749	MKBK01000000	16					98/100	91/81		99/100	98/100	99/100	98/100		B1	Unknown, US
78	CCN443	FBHK01000000	16					98/100	89/85	93/100	99/100	98/100	99/100	98/100	99/100	B1	Poultry farm, UK
79	Neck Skin	CZIT01000000	12					98/100	89/85	93/100	99/100	98/100	99/100	98/100	99/100	B1	Chicken, Spain
80	Faeces	CZIP01000000	17					98/88	89/85	93/100	99/100	98/100	99/100	98/100	99/100	B1	Chicken, Spain
81	Neck Skin	CZHH01000000	31					98/100	89/85	93/100	99/100	98/100	99/90	98/100	99/100	B1	Chicken, Spain
82	Faeces	CZHM01000000	15					98/100	91/81	92/91	99/100	98/100	99/91	98/100	99/100	B1	Chicken, Spain
83	Meat	CZHQ01000000	44					98/100	89/85	93/100	99/100	98/100	99/92	98/100	99/100	B1	Chicken, Spain
84	OXC6580	CUTU01000000	1					98/100	91/81	93/100	99/100	98/100	99/100	98/100		B1	Faeces, UK
85	OXC6495	CUQO01000000	2					98/100	89/85	93/100	99/100	98/100	99/100	98/100		B1	Faeces, UK
86	OXC6403	CUNE01000000	1					98/100	89/85	93/100	99/100	98/100	99/100	98/100	99/100	B1	Faeces, UK
87	OXC6470	CUPN01000000	1					98/100	89/85	93/100	99/100	98/100	99/100	98/100	99/100	B1	Faeces, UK
88	OXC6488	CUQH01000000	1					98/100	89/85	93/100	99/100	98/100	99/100	98/100	99/100	B1	Faeces, UK
89	OXC6487	CUQG01000000	1					98/100	89/85	93/100	99/100	98/100	99/100	98/100	99/100	B1	Faeces, UK
90	OXC6556	CUSU01000000	1					98/100	91/81	93/100	99/100	98/100	99/100	98/100	99/100	B1	Faeces, UK
91	OXC6465	CUPH01000000	1					98/100	91/81	93/100	99/100	98/100	99/100	98/100	99/100	B1	Faeces, UK
92	OXC6547	CUTX01000000	1					98/100	91/81	93/100	99/100	98/100	99/100	98/100	99/100	B1	Faeces, UK
93	OXC6427	CUOE01000000	1					98/100	91/81	93/100	99/100	98/100	99/100	98/100	99/100	B1	Faeces, UK
94	OXC6272	CUIE01000000	1					98/100	91/81	93/100	99/100	98/100	99/100	98/100	99/100	B1	Faeces, UK
95	OXC6269	CUIC01000000	1					98/100	91/81	93/100	99/100	98/100	99/100	98/100	99/100	B1	Faeces, UK
96	OXC6348	CULQ01000000	1					98/100	91/81	93/100	99/100	98/100	99/100	98/100	99/100	B1	Faeces, UK
97	OXC6382	CUMK01000000	1					98/100	91/81	93/100	99/100	98/100	99/100	98/100	99/100	B1	Faeces, UK
98	OXC6632	CUVS01000000	1					98/100	89/85	93/100	99/100	98/100	99/100	98/100	99/100	B1	Faeces, UK
99	OXC6284	CUIS01000000	1					98/100	91/81	93/100	99/100	98/100	99/100	98/100	99/100	B1	Faeces, UK
100	OXC6549	CUUQ01000000	1					98/100	89/85	93/100	99/100	98/100	99/100	98/100	99/100	B1	Faeces, UK
101	OXC6638	CUVY01000000	1					98/100	89/85	93/100	99/100	98/100	99/100	98/100	99/100	B1	Faeces, UK
102	OXC6422	CUOA01000000	1					98/100	91/81	93/100	99/100	98/100	99/100	98/100	99/100	B1	Faeces, UK
103	OXC6354	CUOG01000000	1					98/100	91/81	93/100	99/100	98/100	99/100	98/100	99/100	B1	Faeces, UK
104	OXC6462	CUPE01000000	1										99/100			B1	Faeces, UK
105	OXC6411	CUNO01000000	1										99/100			B1	Faeces, UK
106	OXC6389	CUMS01000000	1										99/100			B1	Faeces, UK
107	OXC6421	CUNZ01000000	1										99/100			B1	Faeces, UK
108	OXC6356	CULI01000000	1										99/100			B1	Faeces, UK
109	OXC6345	CULF01000000	1										99/100			B1	Faeces, UK
110	OXC6265	CUHW01000000	1										99/100			B1	Faeces, UK
111	OXC6377	CUMF01000000	1										99/100			B1	Faeces, UK
112	OXC6252	CUIL01000000	1										99/100			B1	Faeces, UK
113	OXC6328	CUKM01000000	1										99/100			B1	Faeces, UK
114	LMG 23218	AIOB01000000	3										99/100			B1	Chicken, Belgium
115	ATCC 33560	AIOL01000000	51, 83						99/100				99/100			B1	Bovine, Belgium
116	2008-988	AIOS01000000	46, 48										99/100			B1	Human, France
117	1997-4	AIOW01000000	45, 62										99/100			B1	Human, France
118	140-16	AIPF01000000	31, 53										99/100			B1	Human, US
119	1893	AIPK01000000	12										99/100			B1	Cow, US

Class B2

No.	*C. jejuni* Str`ain	Accession no.	Contig no.	51	17	14	15	5	6	7	8	9	5-II	10	11	Class	Host, Country
1	10227	AUUI01000000	2					97/100	99/100	99/100	99/100	98/100	99/100	98/100	98/100	B2	Human, Viet Nam
2	Cj1	AUUL01000000	1					97/100	99/100	99/100	99/100	98/100	99/100	98/100	98/100	B2	Human, Thailand
3	129-258	AINY01000000	19, 62					99/100	99/100	99/100	100/100	100/100	100/100	100/100	99/100	B2	Bovine, US
4	81-176-UMCW7	AZNS01000000	16					99/100	99/100	99/100	100/100	100/100	100/100	100/100	99/100	B2	Chicken, US
5	OXC6333	CUKS01000000	1					100/100	100/100	100/100	100/100	100/100	99/100	100/100	100/100	B2	Faeces, UK
6	OXC6417	CUNU01000000	1					99/100	100/100	99/100	100/100	100/100	99/100	100/100	99/100	B2	Faeces, UK
7	OXC6415	CUNS01000000	1					100/100	100/100	100/100	100/100	100/100	100/100	100/100	100/100	B2	Faeces, UK
8	OXC6409	CUNM01000000	2					99/100	100/100	99/100	100/100	100/100	99/100	100/100	99/100	B2	Faeces, UK
9	OXC6569	CUTI01000000	1					98/100	100/100	99/100	100/100	99/100	99/100	100/100	99/100	B2	Faeces, UK
10	OXC6350	CUMO01000000	1					100/100	100/100	100/100	100/100	100/100	99/100	100/100	100/100	B2	Faeces, UK
11	OXC6423	CUOC01000000	2					97/100	99/100	99/100	99/100	98/100	99/100	98/100	98/100	B2	Faeces, UK
12	OXC6388	CUMR01000000	1					100/100	100/100	100/100	100/100	100/100	100/100	100/100	100/100	B2	Faeces, UK
13	OXC6271	CUID01000000	1					97/100	99/100	99/100	98/100	98/100		99/100	99/100	B2	Faeces, UK
14	Neck	CZJC01000000	16					99/100	100/100	99/100	100/100	100/100	100/100	100/100	99/100	B2	Chicken, Spain
15	Meat	CZJJ01000000	17					99/100	100/100	99/100	100/100	100/100	100/100	100/100	99/100	B2	Chicken, Spain
16	BCW_6891	MJYW01000000	15					97/100	99/100	99/100	99/100	98/100	99/100	98/100	98/100	B2	Chicken, US
17	BCW_6929	MKAU01000000	1					97/100	99/100	99/100	99/100	98/100	99/100	98/100	98/100	B2	Caprine, US
18	BCW_5166	MKHU01000000	26					97/100	99/100			98/100	99/100	98/100	98/100	B2	Human, US
19	CDPHFDLB-F15M00521-2	MOUT01000000	12					99/100	99/100	99/100	100/100	100/100	100/100	100/100	99/100	B2	Environment, US
20	CDPHFDLB-F15M00592	MOUR01000000	1					99/100	99/100	99/100	100/100	100/100	100/100	100/100	99/100	B2	Cow, US
21	CDPHFDLB-F15M00601	MOUO01000000	1					99/100	99/100	99/100	100/100	100/100	99/100	100/100	99/100	B2	Cow, US
22	CDPHFDLB-F15M00602	MOUN01000000	1					99/100	99/100	99/100	100/100	100/100	99/100	100/100	99/100	B2	Cow, US
23	CDPHFDLB-F15M00565-1	MOVQ01000000	2, 4					99/100	99/100	99/100	100/100	100/100	100/100	100/100	99/100	B2	Cow, US
24	CDPHFDLB-F15M00565-3	MOVN01000000	3					99/100	99/100	99/100	100/100	100/100	100/100	100/100	99/100	B2	Cow, US
25	BCW_5155	MKFA01000000	58					97/100	100/100	99/100	99/100	98/100	99/100	98/100	98/100	B2	Human, US
26	C3	NFPV01000000	20					97/100	99/100	99/100	99/100	98/100	99/100	98/100	98/100	B2	Faeces, Canada
27	BCW_3803	MJVR01000000	19					98/100	100/100	99/100	100/100	100/100	100/100	100/100	99/100	B2	Crow, US
28	BCW_4456	MKAI01000000	29					97/100	-	94/100	98/100	97/100	99/100	98/100	99/100	B2	Faeces, US
29	BCW_4734	MKBC01000000	18, 20					100/100	100/100	100/100	100/100	100/100	100/100	100/100	100/100	B2	Unknown, US
30	BCW_5170	MKHW01000000	36					97/100	99/100	99/100	99/100		99/100	98/100	98/100	B2	Human, US
31	Faeces	CZJE01000000	14					99/100	100/100	99/100	100/100	100/100	100/91	100/100	99/100	B2	Chicken, Spain
32	OXC6512	CURH01000000	3, 4					99/100	100/100	99/100	100/100	100/100	100/100	100/100	99/100	B2	Faeces, UK

Class C

No.	*C. jejuni* Strain	Accession no.	Contig no.	51	17	14	15	5	6	7	8	9	5-II	10	11	Class	Host, Country
1	FDAARGOS_263	CP022077.1				100/100	100/100		100/100	100/100	99/100	100/100		100/100	100/100	C	Human, US
2	6399	CAFT01000000	19, 22			100/100	99/100					100/17		100/100	99/100	C	Unknown, Germany
3	OXC6532	CUSD01000000	1			100/100	99/100		100/100	100/100		100/100		99/100	99/100	C	Faeces, UK
4	OXC6633	CUVT01000000	1			100/100	99/100		99/100	100/100		100/100		100/100	99/100	C	Faeces, UK
5	OXC6508	CURD01000000	1			100/100	99/100		100/100	100/100	99/34	100/100		100/100	99/100	C	Faeces, UK
6	OXC6538	CUSK01000000	1			100/100	100/100		99/100	100/100		100/100		99/100	99/100	C	Faeces, UK
7	OXC6257	CUKO01000000	1			100/100	99/100		99/100	100/100		100/100		99/100	99/100	C	Faeces, UK
8	OXC6573	CUTN01000000	1			100/100	99/100		99/100	100/100		100/100		100/100	99/100	C	Faeces, UK
9	OXC6625	CUVL01000000	1			100/100	99/100		100/100	100/100		100/100		100/100	99/100	C	Faeces, UK
10	OXC6516	CURM01000000	1			100/100	99/100		99/100			100/100		100/100	99/100	C	Faeces, UK
11	G113	AQPK01000000	8			100/100	100/100		99/100	100/100		100/100		100/100	99/100	C	Unknown
12	G1	JRLT01000000	1			100/100	100/100		99/100	100/100		100/100		99/100	99/100	C	Human, UK
13	OXC6564	CUTD01000000	1			100/100	99/100		99/100	100/100		99/100		100/100	99/100	C	Faeces, UK
14	OXC6543	CUSR01000000	1			100/100	100/100		99/100	100/100		100/100		99/100	99/100	C	Faeces, UK
15	OXC6527	CURY01000000	1			100/100	100/100		99/100	100/100		100/100		99/100	99/100	C	Faeces, UK
16	OXC6387	CUMQ01000000	1			100/100	100/100		99/100	100/100		100/100		99/100	99/100	C	Faeces, UK
17	OXC6266	CUHX01000000	1			100/100	100/100		100/100	100/100		100/100		99/100	99/100	C	Faeces, UK
18	OXC6590	CUUC01000000	1			100/100	100/100		99/100	100/100		100/100		99/100	99/100	C	Faeces, UK
19	OXC6522	CURS01000000	1			100/100	99/100		99/100			100/100		99/100	99/100	C	Faeces, UK
20	OXC6636	CTRT01000000	1			100/100	100/100		99/100	100/100		100/100		99/100	99/100	C	Faeces, UK
21	OXC6615	CUVC01000000	1			100/100	100/100		99/100	100/100		100/100		99/100	99/100	C	Faeces, UK
22	OXC6449	CUPU01000000	1			100/100	100/100		99/100	99/100		100/100		99/100	99/100	C	Faeces, UK
23	OXC6429	CUOI01000000	1			100/100	99/100		100/100	100/100		100/100		99/100	99/100	C	Faeces, UK
24	OXC6394	CUMU01000000	1			100/100	100/100		100/100	100/100		100/100		99/100	99/100	C	Faeces, UK
25	OXC6456	CUSI01000000	2			100/100	100/100		99/100	100/100		100/100		99/100	99/100	C	Faeces, UK
26	OXC6303	CUJL01000000	1			100/100	100/100		99/100	100/100		100/100		99/100	99/100	C	Faeces, UK

27	OXC6539	CUSL01000000	1			100/100	99/100		99/100	100/100		100/100		100/100	99/100	C	Faeces, UK
28	OXC6602	CUUP01000000	1			100/100	99/100		100/100	100/100		100/100		100/100	99/100	C	Faeces, UK
29	OXC6370	CULX01000000	1			100/100	100/100		99/100	100/100		100/100		99/100	99/100	C	Faeces, UK
30	OXC6553	CUSP01000000	1			100/100	99/100		99/100	100/100		100/100		99/100	99/100	C	Faeces, UK
31	OXC6464	CUPG01000000	1			100/100	100/100		99/100	100/100		100/100		99/100	99/100	C	Faeces, UK
32	OXC6530	CUSB01000000	1			100/100	100/100		99/100	100/100		100/100		99/100	99/100	C	Faeces, UK
33	OXC6563	CUTC01000000	1			100/100	99/100		99/100	100/100		100/100		100/100	99/100	C	Faeces, UK
34	OXC6629	CUVP01000000	1			100/100	99/100		100/100	100/100		100/100		100/100	99/100	C	Faeces, UK
35	OXC6603	CUUR01000000	1			100/100	100/100		99/100	100/100		100/100		99/100	99/100	C	Faeces, UK
36	OXC6500	CUQV01000000	2			100/100	100/100		99/100	100/100		100/100		99/100	99/100	C	Faeces, UK
37	OXC6571	CUTK01000000	1			100/100	100/100		99/100	100/100		100/100		99/100	99/100	C	Faeces, UK
38	OXC6251	CUIA01000000	1			100/100	99/100		99/100	100/100		100/100		99/100	99/100	C	Faeces, UK
39	OXC6292	CUIZ01000000	1			100/100	100/100		100/100	100/100		100/100		99/100	99/100	C	Faeces, UK
40	OXC6565	CUTE01000000	1			100/100	100/100		99/100	100/100		99/100		99/100	99/100	C	Faeces, UK
41	OXC6524	CURU01000000	1			100/100	100/100		100/100	100/100		100/100		99/100	99/100	C	Faeces, UK
42	OXC6282	CUIP01000000	1			100/100	99/100		99/100	100/100		100/100		99/100	99/100	C	Faeces, UK
43	OXC6600	CUUN01000000	1			100/100	100/100		99/100	100/100		99/100		99/100	99/100	C	Faeces, UK
44	OXC6277	CUIJ01000000	1			100/100	100/100		99/100	100/100		100/100		99/100	99/100	C	Faeces, UK
45	OXC6616	CUVB01000000	1			100/100	99/100		99/100	100/100		99/100		100/100	99/100	C	Faeces, UK
46	OXC6310	CUJT01000000	1			100/100	99/100		99/100	100/100		100/100		99/100	99/100	C	Faeces, UK
47	CVM 41943	JAKQ01000000	3			100/100	100/100		99/100	100/100		100/100		99/100	99/100	C	Human, US
48	CDPHFDLB-F15M00600	MOUP01000000	1			100/100	100/100		100/100	99/100		100/100		99/100	99/100	C	Cow, US
49	CDPHFDLB-F15M01862-D	MOUA01000000	1			100/100	100/100		99/100	100/100		100/100		99/100	99/100	C	Goat Milk, US
50	CDPHFDLB-F15M01873-A	MOTZ01000000	2			100/100	100/100		99/100	100/100		100/100		99/100	99/100	C	Goat Milk, US
51	CJ003CC21	MLDJ01000000	6			100/100	100/100		99/100	100/100		100/100		99/100	99/100	C	Human, Finland
52	CJ070CC21	MLDM01000000	5			100/100	100/100		100/100	100/100	99/39	100/100		99/100	99/100	C	Human, Finland
53	CJ035CC21	MLDL01000000	6			100/100	100/100		100/100	100/100		100/100		99/100	99/100	C	Human, Finland
54	CJ507CC21	MLDP01000000	3			100/100	99/100		99/100	100/100	99/85	100/100		99/100	99/100	C	Human, Finland
55	CJ069CC21	MLDQ01000000	8			100/100	99/100		100/100	100/100		100/100		99/100	99/100	C	Human, Finland
56	CJ502CC21	MLDU01000000	1			100/100	100/100		99/100	100/100		100/100		99/100	100/100	C	Human, Finland
57	CJ503CC21	MLDV01000000	1			100/100	100/100		99/100	100/100		100/100		99/100	100/100	C	Human, Finland
58	CJ506CC21	MLDY01000000	9			100/100	100/100		100/100	100/100		100/100		99/100	99/100	C	Human, Finland
59	CJ505CC21	MLDX01000000	6			100/100	99/100		99/100	100/100		100/100		99/100	99/100	C	Human, Finland
60	CJ097CC21	MLDZ01000000	1			100/100	100/100		99/100	99/100		100/100		99/100	99/100	C	Human, Finland
61	CJ504CC21	MLDW01000000	14			100/100	99/100		99/100	100/100		100/100		99/100	99/100	C	Human, Finland
62	D7331	MORJ01000000	8			100/100	99/100		99/100	100/100		100/100		99/100	99/100	C	Stool, US
63	BCW_5157	MKFC01000000	21, 47			100/100	100/100		99/100	100/100		100/100		99/100	99/100	C	Human, US
64	Water	CZJK01000000	2			100/100	100/100									C	Environment, Spain
65	CDPHFDLB-F15M01909	MOTW01000000	1			100/100	100/94									C	Goat, US
66	OXC6393	CUMX01000000	1				99/100		100/100	100/100		100/100		99/100	99/100	C	Faeces, UK
67	OXC6459	CUPC01000000	1				99/100		99/100	100/100		100/100		99/100	99/100	C	Faeces, UK
68	BCW_6920	MKAC01000000	2				100/99		100/100	100/100		100/100		99/100	99/100	C	Bovine, US
69	BCW_6922	MKAE01000000	4				99/99		99/100	100/100		100/100		99/100	99/100	C	Ovine, US
70	BCW_6925	MKAG01000000	7				99/99		99/100	100/100		100/100		99/100	99/100	C	Ovine, US
71	BCW_6933	MKIB01000000	18				100/99		100/100	100/100		100/100		99/100	99/100	C	Sheep, US
72	BCW_6931	MKIC01000000	5				100/99		99/100	100/100		100/100		99/100	99/100	C	Sheep, US
73	BCW_6932	MKID01000000	33				100/99		99/100	100/100		100/100		99/100	99/100	C	Sheep, US
74	11168H	FPEE01000000	36, 37				99/100		100/100	100/100		100/100		100/100		C	Laboratory, UK
75	OXC6392	CUKN01000000	1, 17				99/100		100/100	100/100		100/100		99/100	100/100	C	Faeces, UK
76	OXC6519	CURQ01000000	1, 19				99/100		99/100	100/100		100/100		100/100	99/100	C	Faeces, UK
77	BCW_6924	MKAF01000000	16, 20				100/99		99/100	100/100		100/100		99/100	97/100	C	Ovine, US
78	BCW_6934	MKIA01000000	1, 19				99/99		99/100	100/100		100/100		99/100		C	Goat, US
79	BCW_5156	MKFB01000000	22, 42				100/99		99/100	100/100		100/100		99/100	99/100	C	Human, US

Class M

No.	*C. jejuni* Strain	Accession no.	Contig no.	51	17	14	15	5	6	7	8	9	5-II	10	11	Class	Host, Country
1	BCW_6896	MJYY01000000	34	99/36	96/100					96/100	98/100	99/100		97/100	98/99	M	Crow, US
2	BCW_4223	MJXU01000000	36, 70	95/95												M	Crow, US
3	BCW_6459	MJXH01000000	37, 84	95/95												M	Crow, US
4	BCW_6453	MJXC01000000	5, 68	99/98	96/99											M	Crow, US
5	OXC6561	CUSZ01000000	1	100/100	100/100					100/100	100/100	100/100		99/100	99/100	M	Faeces, UK
6	OXC6639	CUVZ01000000	1	100/100	100/100					100/100	100/100	100/100		99/100	99/100	M	Faeces, UK
7	OXC6364	CULS01000000	1	100/100	100/100					100/100	100/100	100/100		99/100	99/100	M	Faeces, UK

Class R

No.	*C. jejuni* Strain	Accession no.	Contig no.	51	17	14	15	5	6	7	8	9	5-II	10	11	Class	Host, Country
1	OXC6327	CUKK01000000	1							96/100		96/100		97/100	97/100	R	Faeces, UK
2	BCW_6880	MJYO01000000	17									97/94		98/99	97/100	R	Crow, US
3	BCW_4324	MKET01000000	30					99/100	95/99	96/100	99/100	99/100		99/100	98/100	R	Crow, US
4	BCW_3791	MJVJ01000000	53							95/100	98/100	98/100		98/100	97/100	R	Crow, US
5	BCW_3807	MJVT01000000	28							96/100		98/100		98/100	97/100	R	Crow, US
6	BCW_6458	MJXG01000000	41					97/100	95/99	96/100	98/100	98/100		99/100	98/100	R	Crow, US
7	LMG 23211	AIPO01000000	10					100/100	100/100	100/100	100/100	100/100		100/100	100/100	R	Chicken, Belgium
8	CVM N15262	JOUG01000000	2						100/30	100/100		100/100		99/100	99/100	R	Chicken, US
9	CDPHFDLB-F15M01873-B	MOTY01000000	1						99/83	100/100		100/100		99/100	99/100	R	Goat Milk, US
10	BCW_3799	MJVO01000000	9					97/100	95/99	96/100	98/100	98/100		99/100	98/100	R	Crow, US

Draft sequences: LOS Group 2; Classes P, H, E, O & W

Class P

No.	*C. jejuni* Strain	Accession no.	Contig no.	21-25	26	26'	27	28	29-34	Class	Host, Country
1	HB-CJGB -LL	ATBJ01000000	12	98/98		100/100	99/92	100/100	97/99	P	Human, China
2	51494	AINZ01000000	9	97/98		100/100	99/92	99/100	97/99	P	Chicken, US
3	51037	AIPB01000000	12,37	98/98		100/100	99/92	99/100		P	Chicken, US
4	OXC6609	CUUU01000000	1	98/98		100/100	99/92	99/100	97/99	P	Faeces, UK
5	OXC6278	CUIK01000000	2	98/98		100/100	99/92	99/100	99/99	P	Faeces, UK
6	OXC6302	CUJK01000000	1	98/98		100/100	99/92	99/100	97/99	P	Faeces, UK
7	OXC6366	CULT01000000	1	98/98		100/100	99/92	99/100	97/99	P	Faeces, UK
8	OXC6620	CUVF01000000	1	98/98		100/100	99/92	99/100	97/99	P	Faeces, UK
9	OXC6481	CUPY01000000	1	98/98		100/100	99/92	100/100	97/99	P	Faeces, UK
10	OXC6313	CUJW01000000	2	98/98		100/100	99/92	99/100	99/99	P	Faeces, UK
11	OXC6440	CUOT01000000	1	98/98		100/100	99/92	100/100	97/99	P	Faeces, UK
12	OXC6311	CUJU01000000	1	98/98		100/100	99/92	99/100	97/99	P	Faeces, UK
13	OXC6293	CUJA01000000	1	98/98		100/100	99/92	99/100	99/99	P	Faeces, UK
14	OXC6342	CULC01000000	1	98/98		100/100	99/92	100/100	97/99	P	Faeces, UK
15	OXC6365	CULR01000000	1	98/98		100/100	99/92	99/100	99/99	P	Faeces, UK
16	OXC6503	CUQX01000000	1	98/98		99/100	99/92	100/100	97/99	P	Faeces, UK
17	OXC6339	CUKY01000000	1	98/98		100/100	99/92	99/100	99/99	P	Faeces, UK
18	OXC6357	CULJ01000000	1	98/98		100/100	99/92	99/100	97/99	P	Faeces, UK
19	OXC6351	CUMZ01000000	1	98/98		100/100	99/92	99/100	99/99	P	Faeces, UK
20	OXC6396	CUNB01000000	1	98/98		100/100	99/92	99/100	97/99	P	Faeces, UK
21	OXC6259	CUHO01000000	1	98/98		100/100	99/92	99/100	99/99	P	Faeces, UK
22	Neck Skin	CZIL01000000	9			100/100	99/92	100/100		P	Chicken, Spain
23	Neck Skin	CZIB01000000	11,15			100/100	99/92	99/100	97/99	P	Chicken, Spain
24	BCW_3782	MJYK01000000	17	98/98		100/100	99/92	99/100	99/99	P	Crow, US
25	BCW_6475	MJYM01000000	2	96/98		99/100	99/92	99/100	98/99	P	Crow, US
26	BCW_5132	MKBY01000000	29	98/98		100/100	99/92	100/100	97/99	P	Human, US
27	BCW_5131	MKBX01000000	101	98/98		99/100	99/92	99/100	98/99	P	Human, US
28	BCW_5147	MKCJ01000000	117	98/98		99/100	99/92	99/100	98/99	P	Human, US
29	CDPHFDLB-F15M00591	MOUS01000000	1	98/98		100/100	99/92	99/100	99/99	P	Cow, US
30	CDPHFDLB-F15M00554-a2	MOVZ01000000	1	98/98		100/100	99/92	99/100	97/99	P	Water, US
31	CDPHFDLB-F15M00554-a3	MOVY01000000	1	98/98		100/100	99/92	99/100	97/99	P	Water, US
32	CDPHFDLB-F12M00558	MOVX01000000	1	98/98		100/100	99/92	99/100	99/99	P	Cow, US
33	OXC6316	CUJZ01000000	1	98/98		100/100	99/92	99/100	97/99	P	Faeces, UK
34	OXC6274	CUIG01000000	1	98/98		100/100	99/92	99/100	97/99	P	Faeces, UK
35	BCW_4224	MJWH01000000	18	98/98		100/100	99/92	99/100	99/99	P	Crow, US
36	BCW_4757	MKBO01000000	47	98/98		100/100	99/92	99/100	99/99	P	Unknown
37	BCW_5150	MKCL01000000	69	98/98		99/100	99/92	99/100	98/99	P	Human, US
38	W22	NFNK01000000	4	98/98		100/100	99/92	99/100	99/99	P	Water, Canada
39	C16	NFPX01000000	20	98/98		100/100	99/85	99/100	99/99	P	Chicken, Canada

40	A1	NFQG01000000	4			100/100	99/89	99/77		P	Chicken, Canada
41	H23	NFPA01000000	15	97/97		99/100	99/92	99/100	97/99	P	Human, Canada
42	K5	AUUP01000000	15, 450, 451	-		99/100	99/92	99/100	97/99	P	Human, Pakistan

Class H

No.	*C. jejuni* Strain	Accession no.	Contig no.	21-25	26	26'	27	28	29-34	Class	Host, Country
1	30318	AUUJ01000000	26	98/98		99/100	99/100		99/100	H	Human, Viet Nam
2	HB-CJGB-LC	ASXO01000000	1	98/98		100/100	99/100		98/100	H	Human, China
3	2008-894	AIOQ01000000	2	98/98		100/100	99/100		99/100	H	Human, France
4	1997-10	AIOY01000000	42, 101	98/98		99/100	99/100		99/99	H	Human, US
5	1854	AIPJ01000000	14	99/98		99/100	99/100		98/100	H	Cow, US
6	OXC6622	CUVG01000000	2	98/98		99/100	99/100		99/100	H	Faeces, UK
7	OXC6497	CUQR01000000	1	98/98		99/100	99/100		99/100	H	Faeces, UK
8	OXC6628	CUVO01000000	1	99/99		99/100	99/100		98/100	H	Faeces, UK
9	OXC6260	CUHR01000000	1	98/98		99/100	99/100		99/100	H	Faeces, UK
10	OXC6566	CUTF01000000	1	98/98		99/100	99/100		99/100	H	Faeces, UK
11	OXC6520	CURP01000000	1	98/98		99/100	99/100		99/100	H	Faeces, UK
12	OXC6478	CUPW01000000	1	98/98		99/100	99/100		99/100	H	Faeces, UK
13	OXC6611	CUUW01000000	2	98/98		99/100	99/100		99/100	H	Faeces, UK
14	OXC6473	CUPP01000000	1	98/98		99/100	99/100		99/100	H	Faeces, UK
15	OXC6585	CUUA01000000	1	98/98		99/100	99/100		99/100	H	Faeces, UK
16	OXC6438	CUOS01000000	1	98/98		99/100	99/100		99/100	H	Faeces, UK
17	OXC6575	CUTP01000000	1	98/98		99/100	99/100		99/100	H	Faeces, UK
18	OXC6591	CUUE01000000	1	98/98		99/100	99/100		99/100	H	Faeces, UK
19	Faeces	CZIS01000000	78			100/97	99/92			H	Chicken, Spain
20	Meat	CZHK01000000	31	99/98		100/99	99/100		98/100	H	Chicken, Spain
21	Meat	CZHZ01000000	4			100/99			98/99	H	Chicken, Spain
22	Faeces	CZIV01000000	34	99/96		100/99	99/100		98/100	H	Chicken, Spain
23	Meat	CZHN01000000	1			99/100	99/100		99/99	H	Chicken, Spain
24	CVM 41921	JAKK01000000	34	99/96		99/100	99/100			H	Human, US
25	BCW_4230	MJWJ01000000	32	98/98		99/100	99/100		99/100	H	Crow, US
26	BCW_7692	MKAB01000000	26	98/98		99/100	99/100		99/100	H	Human, US
27	BCW_5126	MKBU01000000	52	98/98		99/100	99/100		99/100	H	Human, US
28	CDPHFDLB-F12M00584-a2	MOVC01000000	3	99/98		99/100	99/100		98/100	H	Cream, US
29	CDPHFDLB-F12M00584-a1	MOVD01000000	1	99/98		99/100	99/100		98/100	H	Cream, US
30	CDPHFDLB-F12M00585-a1	MOVB01000000	1	99/98		99/100	99/100		98/100	H	Cream, US
31	CDPHFDLB-F12M00585-a2	MOVA01000000	1	99/98		99/100	99/100		98/100	H	Cream, US
32	CDPHFDLB-F12M00589-a2	MOUY01000000	1	99/98		99/100	99/100		98/100	H	Cream, US
33	CDPHFDLB-F12M00589-a1	MOUZ01000000	1	99/98		99/100	99/100		98/100	H	Cream, US
34	CDPHFDLB-F12M00436-A	MOWK01000000	1	99/98		99/100	99/100		98/100	H	Cow, US
35	CDPHFDLB-F12M00436-D	MOWJ01000000	1	99/98		99/100	99/100		98/99	H	Cream, US
36	CDPHFDLB-F12M00436-H	MOWI01000000	2	99/98		99/100	99/100		98/99	H	Cream, US
37	CDPHFDLB-F12M00521-3g3a	MOWH01000000	1	99/98		99/100	99/100		98/100	H	Milk, US
38	CDPHFDLB-F12M00521-3g3b	MOWG01000000	2	99/98		99/100	99/94			H	Milk, US
39	CDPHFDLB-F12M00521-3k2a	MOWF01000000	1	99/98		99/100	99/100		98/100	H	Milk, US
40	CDPHFDLB-F12M00521-3k2b	MOWE01000000	1	99/98		99/100	99/100		98/100	H	Milk, US
41	CDPHFDLB-F12M00521-8g1a	MOWD01000000	4	99/98		99/100	99/100		98/100	H	Milk, US
42	CDPHFDLB-F12M00521-8g1b	MOWC01000000	1	99/98		99/100	99/100		98/100	H	Milk, US
43	CDPHFDLB-F12M00521-8k1a	MOWB01000000	1	99/98		99/100	99/100		98/100	H	Milk, US
44	CDPHFDLB-F12M00521-8k1a	MOWA0100000	17	99/98		99/99	99/100		98/100	H	Milk, US
45	CDPHFDLB-F12M00562-b7a1	MOVS01000000	1	99/98		99/100	99/100		98/100	H	Milk, US
46	CDPHFDLB-F12M00562-b7b1	MPBL01000000	31	99/98		99/99	99/100			H	Milk, US
47	CDPHFDLB-F12M00580-a2	MOVJ01000000	1	99/98		99/100				H	Milk, US
48	CDPHFDLB-F12M00582-a1	MOVG01000000	1	99/98		99/100	99/100		98/100	H	Milk, US
49	CDPHFDLB-F12M00582-a2	MOVF01000000	1	99/98		99/100				H	Milk, US
50	CDPHFDLB-F12M00583-b1	MPBK01000000	4	99/98		99/99	99/78			H	Milk, US
51	BCW_6902	MKEU01000000	12	98/98		100/100	99/100		99/100	H	Crow, US
52	S3	NFOF01000000	35	98/98		99/100	99/100		99/100	H	Environment water, Canada
53	H27	NFOW01000000	23, 31	98/98		99/100	99/100		98/99	H	Human, Canada
54	H10	NFPM01000000	87, 98	99/98		99/100	99/100			H	Human, Canada
55	C1	NFQC01000000	47	98/97		100/100	99/100		99/100	H	Chicken, Canada
56	W4	NFMU01000000	30, 80	99/98		99/100	99/95			H	Environment water, Canada
57	H32	NFOQ01000000	8, 72	99/98		99/100	99/100			H	Human, Canada
58	OXC6586	CTRS01000000	1	97/98		99/100	99/100		99/100	H	Faeces, UK

59	OXC6353	CUNV01000000	1	99/98		100/99	99/100		98/100	H	Faeces, UK
60	OXC6314	CUJX01000000	1	99/98		99/100	97/100		98/100	H	Faeces, UK
61	OXC6507	CURC01000000	2	98/98		99/100	99/100		99/100	H	Faeces, UK
62	RC429	CYRQ01000000	33	98/98		99/100	99/98			H	Chicken, UK
63	12502	CYRV01000000	11	98/98		99/100	99/100		99/100	H	Chicken, UK
64	BCW_4221	MJWI01000000	5	98/98		99/100	99/100		99/100	H	Crow, US
65	BCW_5125	MKBT01000000	14	98/98		99/100	99/100		99/100	H	Human, US
66	BCW_5145	MKCH01000000	42	98/98		99/100	99/100		99/100	H	Human, US
67	BCW_5161	MKHS01000000	33	98/98		99/100	99/100		99/100	H	Human, US
68	BCW_5162	MKHT01000000	31	98/98		99/100	99/100		99/100	H	Human, US
69	OXC6588	CUUG01000000	1	98/98		99/100	99/100		99/100	H	Human, US
70	BH-01-0142	ABKD01000000	1	98/98		99/100	99/100		99/100	H	Human, Thailand

Class E

No.	*C. jejuni* Strain	Accession no.	Contig no.	21-25	26	26'	27	28	29-34	Class	Host, Country
1	1997-14	AIPA01000000	2, 30	98/100	99/100		98/92	99/100		E	Human, US
2	OXC6373	CUMA01000000	1	96/100	99/100		98/92	99/100	98/99	E	Faeces, UK
3	OXC6330	CUKP01000000	1	96/100	99/100		98/92	99/100	98/99	E	Faeces, UK
4	OXC6294	CUJB01000000	1	96/100	99/100		98/92	99/100	98/99	E	Faeces, UK
5	OXC6510	CURE01000000	1	96/100	99/100		98/92	99/100	98/99	E	Faeces, UK
6	OXC6511	CURG01000000	2	95/100	99/100		98/92	99/100	98/99	E	Faeces, UK
7	OXC6437	CUOQ01000000	1	96/100	99/100		98/92	99/100	98/99	E	Faeces, UK
8	OXC6369	CULV01000000	2	96/100	99/100		98/92	99/100	98/99	E	Faeces, UK
9	Faeces	CZIK01000000	72	96/98	99/100		98/92	99/77		E	Chicken, Spain
10	Neck Skin	CZIW01000000	31	96/98	99/100		98/92	99/100		E	Chicken, Spain
11	BCW_6898	MJZA01000000	29	96/100	99/100		98/92	99/100	98/99	E	Chicken, Spain
12	BCW_6899	MJZB01000000	17	96/100	99/100		98/92	99/100	98/99	E	Chicken, Spain
13	W38	NFMV01000000	12	96/97	99/100		98/92	99/100	98/99	E	Environment water, Canada
14	W32	NFNB01000000	24	96/100	99/100		98/92	99/100	98/99	E	Environment water, Canada
15	BCW_4743	MKBH01000000	18	96/100	99/100		98/92	99/100	98/99	E	Human, US

Class O

No.	*C. jejuni* Strain	Accession no.	Contig no.	21-25	26	26'	27	28	29-34	Class	Host, Country
1	LMG 9872	AIPM01000000	11, 18	98/100	99/100		98/100		98/99	O	Human, Sweden
2	OXC6536	CUSH01000000	3	99/100	100/100		100/100		100/100	O	Faeces, UK
3	OXC6332	CUKR01000000	1	98/100	99/100		98/100		98/100	O	Faeces, UK
4	5070	CCXG01000000	6	98/100	99/100		98/100		98/100	O	Chicken, Finland
5	BCW_4753	MKBM01000000	8	99/100	100/100		100/100		99/100	O	Unknown, US
6	W27	NFNH01000000	5	96/97	99/100		98/75			O	Environment water, Canada
7	OXC6321	CUKG01000000	2	99/100	100/100		100/100		100/100	O	Faeces, UK

Class W

No.	*C. jejuni* Strain	Accession no.	Contig no.	21-25	26	26'	27	28	29-34	Class	Host, Country
1	7092_1	CUPI01000000	1	99/85	100/100	99/99		99/100		W	Faeces, UK
2	CVM 41910	JAKG01000000	18	99/85	99/100	99/99		98/100		W	Human, US
3	CVM 41914	JAKI01000000	10	99/85	99/100	99/99		98/100		W	Human, US
4	CVM 41933	JAKN01000000	2	98/85	99/100	99/99		99/100		W	Human, US
5	CVM 41973	JAJF01000000	9	99/85	99/100	99/97				W	Human, US
6	CVM 41964	JAKR01000000	2	99/85	99/100	99/99		98/100		W	Human, US
7	CVM 41985	JAKU01000000	14		99/100	99/99		98/100		W	Human, US
8	BCW_4452	MJWG01000000	16	99/85	100/100	99/99		99/100		W	Faeces, US
9	BCW_6882	MJYR01000000	6	95/85	99/100	99/100		99/100		W	Crow, US
10	BCW_6886	MJYU01000000	64	95/85	99/100	99/100		99/100		W	Crow, US
11	BCW_6953	MJZN01000000	37	98/85	99/100	99/99		99/100		W	Faeces, US
12	BCW_6959	MJZT01000000	2	98/85	99/100	99/99		99/100		W	Faeces, US

LOS Group 2; Mix LOS Classes

No.	*C. jejuni* Strain	Accession no.	Contig no.	21-25	26	26'	27	28	29-34	Class	Host, Country
1	CVM 41975	JAKT01000000	2			99/99		98/100		EHOP	Human, US
2	BCW_6883	MJYS01000000	5	95/85		99/99		99/100		EHOP	Crow, US
3	BCW_6885	MJYT01000000	58	96/85		99/99		98/100		EHOP	Crow, US
4	BCW_3781	MEIB01000000	59	95/85		99/99		99/100		EHOP	Crow, US
5	BCW_3784	MEIC01000000	25	96/85		98/99		98/100		EHOP	Crow, US
6	W9	NFMI01000000	1, 6	99/97						EHOP	Environment water, Canada

Draft sequences: LOS Group 3; Classes D, F, K, N, Q, I, J & S

Class D/F

No.	*C. jejuni* Strain	Accession no.	Contig no.	17	18	19	20	38	40	41	42-45	46	49-50	16	Class	Host, Country
1	OXC6583	CUTY01000000			96/94	98/100	97/100							99/100	F	Unknown, UK
2	OXC6319	CUKC01000000			96/94	98/100	97/100							99/100	F	Pigeon, Sweden
3	OXC6254	CUJH01000000	2		98/94	99/100	98/100							99/100	F	Faeces, UK
4	OXC6582	CUTW01000000	1	100/100	100/100	100/100	100/100							99/100	D	Faeces, UK
5	CDPHFDLB-F15M00665P1	MOUL01000000	1		98/94	99/100	98/100							99/100	F	Faeces, UK
6	CDPHFDLB-F15M00667P1	MOUJ01000000	3		98/94	99/100	98/100							99/100	F	Faeces, UK
7	CDPHFDLB-F15M00668P1	MOUH01000000	1		98/94	99/100	98/100							99/100	F	Cow Milk, US
8	CDPHFDLB-F15M00669P1	MOUF01000000	1		98/94	99/100	98/100							99/100	F	Cow Milk, US
9	CDPHFDLB-F15M00562-b9b1a	MOVR01000000	1		98/94	99/100	98/100							99/100	F	Cow Milk, US
10	CDPHFDLB-F12M00566-a1	MOVM01000000	1		98/94	99/100	98/100							99/100	F	Cow Milk, US
11	CDPHFDLB-F15M00668P2	MOUG01000000	2		98/94	99/100	98/100							99/100	F	Cow, US
12	RC507	CYRT01000000	1		98/86	99/100	98/100							99/100	F	Cow, US
13	W30	NFND01000000	2		98/94	99/100	98/100							99/100	F	Cow Milk, US
14	OXC6608	CUUS01000000	46, 20		98/77	99/100	99/100							99/100	F	Chicken, UK
15	OXC6432	CUOJ01000000	8		98/94	99/100	98/100							99/100	F	Environment water, Canada
16	OXC6412	CUNP01000000	1		98/94	99/100	99/100							99/100	F	Faeces, UK
17	OXC6491	CUQK01000000	3		98/94	99/100	99/100							99/100	F	Faeces, UK
18	OXC6250	CUHN01000000	1		98/94	99/100	98/100							99/100	F	Faeces, UK
19	OXC6450	CUQF01000000	2		98/94	99/100	98/100							99/100	F	Faeces, UK
20	OXC6401	CUND01000000	1		98/94	99/100	98/100							99/100	F	Faeces, UK
21	OXC6362	CULO01000000	2		98/94	99/100	98/100							99/100	F	Faeces, UK
22	OXC6469	CUPM01000000	1		98/94	99/100	98/100							99/100	F	Faeces, UK
23	OXC6404	CUNG01000000	1		98/94	99/100	98/100							99/100	F	Faeces, UK
24	OXC6375	CUMD01000000	2		98/94	99/100	98/100							99/100	F	Faeces, UK
25	OXC6441	CUOU01000000	1		98/94	99/100	98/100							99/100	F	Faeces, UK
26	OXC6368	CULW01000000	2		98/94	99/100	98/100							99/100	F	Faeces, UK
27	11601MD	LKCR01000000	1		98/94	99/100	99/100							99/100	F	Faeces, UK
28	XWM	EF176584.1			99/100	100/100	100/100							99/100	F	Unknown, China
29	Faeces	CZHX01000000	1		98/94	99/100	99/100							99/100	F	Faeces, UK
30	NC05-27	BCNK01000000	19		98/94	99/100								99/100	F	Turkey, US

Class K

No.	*C. jejuni* Strain	Accession no.	Contig no.	17	18	19	20	38	40	41	42-45	46	49-50	16	Class	Host, Country
1	7065_7	CUOO01000000	1	96/100	93/100	95/98							99/100		K	Faeces, UK
2	7213_3	CUTB01000000	1	96/100	93/100	95/98							99/100		K	Faeces, UK
3	7092_1	CURB01000000	1	96/100	93/100	95/98							99/100		K	Faeces, UK
4	7038_3	CUIM01000000	1	96/100	94/100	95/98							99/100		K	Faeces, UK
5	7065_7	CUMJ01000000	1	96/100	94/100	95/98							99/100		K	Faeces, UK
6	7065_7	CULP01000000	1	96/100	93/100	95/98							99/100		K	Faeces, UK
7	7213_3	CUVJ01000000	1	96/100	93/100	95/98							99/100		K	Faeces, UK
8	Meat	CZHP01000000	32			95/98							99/100		K	Chicken, Spain

9	CVM 41927	JAJG01000000	10, 15										99/94		K	Human, US
10	BCW_6476	MJYN01000000	39	94/100	92/100	95/98							93/100		K	Crow, US
11	BCW_6887	MKEV01000000	26		93/100	95/98							99/100		K	Crow, US
12	RC51	CYRU01000000	80	96/100		95/98							99/100		K	Chicken, UK
13	BJ-CJD39	LISI01000000	1	96/100	93/100	95/98							99/100		K	Human, China

Class N

No.	*C. jejuni* Strain	Accession no.	Contig no.	17	18	19	20	38	40	41	42-45	46	49-50	16	Class	Host, Country
1	BCW_5172	MKHY01000000	8					88/83						95/99	N	Human, US

Class Q

No.	*C. jejuni* Strain	Accession no.	Contig no.	17	18	19	20	38	40	41	42-45	46	49-50	16	Class	Host, Country
1	2871	LLWN01000000	1		91/94	91/100	93/100					99/100		79/80	Q	Poultry, Malaysia

Class I

No.	*C. jejuni* Strain	Accession no.	Contig no.	17	18	19	20	38	40	41	42-45	46	49-50	16	Class	Host, Country
1	LMG 23263	AIOD01000000	26, 69	99/100	99/85						99/100				I	Chicken, US
2	OXC6374	CUMB01000000	1	99/100	99/100	98/100	96/100		100/100	100/100	99/100				I	Faeces, UK
3	OXC6480	CUQA01000000	1	96/100	98/100	98/100	96/100		98/100	99/100	97/99				I	Faeces, UK

Class J

No.	*C. jejuni* Strain	Accession no.	Contig no.	17	18	19	20	38	40	41	42-45	46	49-50	16	Class	Host, Country
1	OXC6518	CURO01000000	1		99/92	99/100	96/90		98/98		96/100				J	Faeces, UK
2	BCW_6457	MJXF01000000	14, 62						97/100		95/99				J	Crow, US
3	BCW_4744	MKBI01000000	29		98/94	99/100	96/100		98/98		97/100				J	Crow, US
4	W13	NFNU01000000	94, 99						97/86		99/100				J	Environment water, Canada

Class S

No.	*C. jejuni* Strain	Accession no.	Contig no.	17	18	19	20	38	40	41	42-45	46	49-50	16	Class	Host, Country
1	OXC6287	CUIU01000000	1		98/94	99/100	96/100		98/100	99/100	96/100				S	Faeces, UK
2	NW	AGTE01000000	2, 4						98/100	99/100	96/100				S	Human, US
3	OXC6418	CUNW01000000	1		98/94	99/100	96/100		98/100	99/100	96/100				S	Faeces, UK
4	OXC6517	CURL01000000	2		98/94	99/100	96/100		98/100	99/100	96/100				S	Faeces, UK
5	OXC6451	CUQQ01000000	2		98/94	99/100	96/100		98/100	99/100	96/100				S	Faeces, UK
6	OXC6529	CUSA01000000	1, 21		98/94	99/100	96/100		98/100	99/100	96/100				S	Faeces, UK

LOS Group 3; Mix LOS Classes

No.	*C. jejuni* Strain	Accession no.	Contig no.	17	18	19	20	38	40	41	42-45	46	49-50	16	Class	Host, Country
1	VA48	NACK01000000	2, 5						99/79	99/100	95/99				I/S	Water, Sweden
2	BCW_6451	MJXA01000000	7	99/100	99/100	98/100	96/100		99/100		97/100				I/J	Faeces, UK
3	BCW_6452	MJXB01000000	6	99/100	99/100	98/100	96/100		99/100		97/100				I/J	Faeces, UK
4	BCW_6954	MJZO01000000	26, 27	99/100	99/100	98/100	98/100		98/98		95/100				I/J	Faeces, UK

Draft sequences: LOS Group 4; Classes G & L

Class G

No.	*C. jejuni* Strain	Accession no.	Contig no.	35	36	37	38	47	48	16	Class	Host, Country
1	OXC6358	CULK01000000	1	99/100	99/100	99/100	100/100			99/100	G	Faeces, UK
2	OXC6498	CUQT01000000	1	98/100	99/100	99/100	99/100			99/100	G	Faeces, UK
3	OXC6614	CUVA01000000	1	99/100	99/100	99/100	100/100			99/100	G	Faeces, UK
4	OXC6360	CULM01000000	1	98/100	99/100	99/100	99/100			99/100	G	Faeces, UK
5	OXC6322	CUKF01000000	1	98/100	99/100	99/100	99/100			99/100	G	Faeces, UK
6	OXC6410	CUNN01000000	1	99/100	99/100	99/100	99/100			99/100	G	Faeces, UK
7	OXC6486	CUQE01000000	2	98/100	99/100	99/100	99/100			99/100	G	Faeces, UK
8	OXC6256	CUKD01000000	5, 20	98/100	99/100	99/90	99/100			99/100	G	Faeces, UK
9	JL-CJHLIU1-1	LISQ01000000	2	98/100	99/100	99/100	99/100			99/100	G	Chicken, China
10	BCW_3794	MJVL01000000	3	97/100	99/100	98/100	98/100			99/100	G	Crow, US

Class L

No.	*C. jejuni* Strain	Accession no.	Contig no.	35	36	37	38	47	48	16	Class	Host, Country
1	BCW_3800	MJVP01000000	64	98/100	98/100	89/100		99/100	98/100	92/93	L	Crow, US
2	BCW_3802	MJVQ01000000	56	98/100	98/100	89/100		99/100	98/100	92/93	L	Crow, US
3	BCW_3810	MJVU01000000	57	98/100	98/100	89/100		99/100	99/100	92/93	L	Crow, US
4	BCW_4231	MJXZ01000000	65	98/100	98/100	89/100		99/100	99/100	92/93	L	Crow, US
5	BCW_6881	MJYQ01000000	89	99/100	97/100	89/100		99/100	98/100	92/93	L	Crow, US
6	OXC6631	CUVR01000000	1	97/100	96/100	89/100		99/100	99/99	91/93	L	Faeces, UK
