# Supplementary material for: Molecular and in silico typing of the lipooligosaccharide biosynthesis gene cluster in Campylobacter jejuni and Campylobacter coli
Source: PLoS One. 2022 Mar 31;17(3):e0265585. doi: 10.1371/journal.pone.0265585 (PMC8970381; doi:10.1371/journal.pone.0265585)
Supplement: S2 Table — (PDF) [file pone.0265585.s002.pdf]

S2 Table. Primers for the identification of *C. jejuni* LOS locus classes

Primer Name	Primer Sequence	Target Genes	Amplicon Size (bps)	Class Specificity	%GC	GC clamp	Dimers	3' Dimers	Stability	Tm (ºC)	Runs	Annealing temp. (ºC)
1	Forward Primer: TGGGAGCAAGCCTTATCG<br>Reverse Primer: TCCCAAGGTCTTTTTTTAATC	*waaM*	184	All	55<br>33	2<br>1	3<br>4	2<br>1	1.8<br>2.7	56<br>52	3<br>7	50
2	Forward Primer: TCATATCTTCCATTTGGATTAAATT<br>Reverse Primer: AAAGGCATTTTTGCTGGTC	waaV	205-220	All	24<br>42	0<br>1	4<br>3	4<br>1	1.7<br>0.7	53.1<br>52.4	3<br>5	50
A1	Forward Primer: AGCTTCTCTTGAAAGCATATTG<br>Reverse Primer: ACAGGATGAAGTTGATTTAGTG	*cgtA* & *cgtB*	1301	A1	36<br>36	1<br>1	4<br>2	2<br>2	3.3<br>1.3	54.7<br>54.7	3<br>3	49.7
A2	Forward Primer: ATTGCCTGCTATTCAAAGAG<br>Reverse Primer: AGGCTGTTGGTTTAATATCG	*cgtA* & *cgtB*	1090	A2	40<br>40	1<br>2	3<br>4	2<br>2	1.9<br>1.9	53.2<br>53.2	3<br>3	48.2
B1	Forward Primer: CCAGCTGCCTTAACTCCTTC<br>Reverse Primer: TCCTAGGGCTATGGCTACTG	*cgtB* & *cst-II*	764	B1	55<br>31	1<br>1	6<br>4	1<br>2	1.6<br>2.2	62<br>56	2<br>2	54.4
B2	Forward Primer: TTAACAAGCACTTCATTCTTAG<br>Reverse Primer: TATAGCAAGGGCAATAGAAAG	*cgtA* & *cgtB*	640	B2	52<br>35	2<br>2	2<br>4	1<br>1	3.3<br>1.9	61<br>55	3<br>3	47.9
CV	Forward Primer: GCTGCTGCTATAGTAGGAAG<br>Reverse Primer: AAATCAAAAAAACCTTTATGCTTTTC	*neuC1* & *neuA1*	931	C and V	50<br>23	1<br>1	6<br>4	2<br>1	1.6<br>1.2	57.3<br>53.8	2<br>3	55
C	Forward Primer: CAGCAAGTAGGATGATAACAG<br>Reverse Primer: CATGATAATTTTCTACAAATCGCACT	Orf4 & Orf15	2287	C	42<br>30	1<br>0	2<br>4	1<br>1	1.2<br>0.7	57<br>60	2<br>4	55
M	Forward Primer: AGACGCTTTGCAAGTTATAATG<br>Reverse Primer: TGATGGAAGTAGCGATAATAG	*waaM* & Orf51	1149	M	36<br>38	1<br>1	6<br>2	2<br>1	3.0<br>3.1	54.7<br>54	3<br>2	49
R	Forward Primer: ATAAGCAATCTTCCGAATTCAC<br>Reverse Primer: TGTTTAAGTTATGGCGAAGATG	*cj1145c* & *waaV*	729	R	36<br>36	1<br>1	6<br>4	1<br>1	1.4<br>2.6	54.7<br>54.7	2<br>3	49.7
EHOP	Forward Primer: TTCAAGAGCGTCCAGAAG<br>Reverse Primer: CGTGAGTTCCTGTGTCAATC	Orf22	453	E, H, O, P	50<br>50	1<br>1	3<br>3	1<br>2	2.1<br>2.5	53.7<br>57.3	2<br>2	48.7
26EO	Forward Primer: TTGCCGTTAATTCATTACAG<br>Reverse Primer: ATGTCGCATTTATACCTTTG	Orf26 & Orf27	1017	E, O	35<br>35	1<br>1	4<br>4	1<br>2	2.8<br>2.2	51.1<br>51.1	2<br>3	60.4
28EP	Forward Primer: TCAGGTAGAGATGCATTTAG<br>Reverse Primer: CGCCTATGCAATGTTTAACC	Orf28 & Orf29	713	E, P	40<br>45	1<br>2	6<br>4	1<br>1	2.2<br>2.5	53.2<br>55.3	3<br>3	57.4
26'HP	Forward Primer: AAAGCAAAGAGAATGGATTAG<br>Reverse Primer: ATGTCGCATTTATACCTTTG	Orf26 & Orf27	904	H, P	33<br>35	1<br>1	3<br>4	1<br>2	2.2<br>2.2	52<br>51.1	3<br>3	59.9
W	Forward Primer: GCTTTGGGCTTATGAGAGTG<br>Reverse Primer: GGCGAACAACTACACCCTATAC	Orf53 & Orf54	1052	W	50<br>50	1<br>1	2<br>4	1<br>1	1.9<br>3.8	57.3<br>60.3	3<br>3	55
D	Forward Primer: TGGTTGGTGGCCTGATTATG<br>Reverse Primer: CAATGCTTGGAATGGTATAG	Orf3 & Orf17	1221	D, (I)	50<br>40	1<br>1	4<br>4	1<br>2	3.4<br>2.6	57.3<br>53.2	2<br>2	48.2
F	Forward Primer: ATATCAAGATCCACCCATAC	Orf 16 & *waaV*	886	F, (D)	40	1	4	1	2.2	53.2	3	51.5

	Reverse Primer: CTCGATGCTTGTGAAATAAC				40	1	4	2	2.0	53.2	3	
IS	Forward Primer: GCAAATGGGAAATCTTGATAGG<br>Reverse Primer: GGCCCTCAGATAAACTACCC	Orf41 & Orf42	973	I, S	40<br>55	2<br>3	3<br>4	1<br>1	1.8<br>2.0	56.5<br>59.4	3<br>3	51.5
J	Forward Primer: TCATCAATACGCTTTAAATTCC<br>Reverse Primer: GGCCCTCAGATAAACTACCC	Orf40 & Orf42	1206	J	31<br>55	2<br>3	6<br>4	1<br>1	1.9<br>2.0	52.8<br>59.4	3<br>3	47.8
K	Forward Primer: ACATAATACTCCTTGCAATC<br>Reverse Primer: TCCCAAGAATCTATAATATCAG	Orf49 & waaV	3118	K	35<br>31	1<br>1	6<br>4	2<br>2	1.2<br>1.5	51.1<br>52.8	2<br>3	46.1
Q	Forward Primer: TGTTGCTAATTTGGCTAATTC<br>Reverse Primer: AAACGGGATTTATGGTAGTTTG	Orf46 & Orf16	1130	Q	33<br>36	1<br>1	4<br>4	1<br>1	2.6<br>2.4	52<br>54.7	3<br>3	47
N	Forward Primer: GCACCAACTCCCAAATGTC<br>Reverse Primer: GAAAGCAGCGATGATACCC	Orf38 & *waaV*	1253	N	52<br>52	1<br>3	2<br>2	1<br>1	1.9<br>1.3	56.7<br>56.7	3<br>3	51.7
G	Forward Primer: TCTGATTGATACAACTTTCTATT<br>Reverse Primer: AAGATGCAAATGAAATCATACC	Orf37 & Orf16	776	G	26<br>31	0<br>2	3<br>4	2<br>1	1.6<br>1.3	55<br>57	3<br>3	50.5
T	Forward Primer: TTGGCAAGATGATTGAAATTTTAGG<br>Reverse Primer: TTGTGAAATAGCGTTTAAAGAG	Orf37 & *waaV*	1773	T, (G, U)	32<br>31	2<br>1	6<br>6	1<br>1	2.2<br>2.1	56.4<br>52.8	4<br>3	47
U	Forward Primer: TATTCTTTGCTGCCAACC<br>Reverse Primer: TTGTGAAATAGCGTTTAAAGAG	Orf38 & waaV	1499	U, (G)	44<br>31	2<br>1	3<br>6	1<br>1	1.3<br>2.1	51.4<br>52.8	3<br>3	46.5
L	Forward Primer: TTTGCTTCTTCTATAAGTAATTTTC<br>Reverse Primer: TTGTGAAATAGCGTTTAAAGAG	Orf16 & *waaV*	1027	L, (F)	24<br>31	1<br>1	4<br>6	1<br>1	1.6<br>2.1	55<br>57	4<br>3	47.8

- *C. jejuni* reference strains, used to design primers: RM1048/ATCC43432 (Accession number: AF215659) for class A1; RM1556/ATCC43438 (Accession number: AF400048) for class A2; RM1050/ATCC43449 (Accession number: AF401529) for class B1; RM1052/ATCC43456 (Accession number: AF401528) for class B2; NCTC11168 (Accession number: AL111168) for class C, RM3418 (Accession number: EU404109) for class D; 81116 (Accession number: CP000814) for class E; RM1221 (Accession number: CP000025) for class F; RM1555/ATCC43437 (Accession number: AY436358) for class G; RM1553/ATCC43435 (Accession number: EU404106) for class H; RM1850 (Accession number: EU404107) for class I; RM1508 (Accession number: EU404104) for class J; RM1861 (Accession number: EU410350) for class K; RM3435 (Accession number: EU404111)) for class L; RM1503 (Accession number: EF140720)) for class M; RM2095 (Accession number: AY816330)) for class N; RM3423 (Accession number: EF143352)) for class O; GB4 (Accession number: AY943308)) for class P, RM3437 (Accession number: EU404112) for class Q; GC149 (Accession number: AY962325) for class R; RM3419 (Accession number: EU404110) for class S; LMG23223 (Accession number: AIOC00000000-contig 1) for class T; 2008-979 (Accession number: AIOU00000000-contigs 12, 88) for class U; 2008-1025 (Accession number: AIOP00000000-contigs 26, 57, 134) for class V; M1 (Accession number: CP001900) for class W.
- Classes given in brackets in the last column were also detectable with respective primers.
