# Supplementary material for: Molecular and in silico typing of the lipooligosaccharide biosynthesis gene cluster in Campylobacter jejuni and Campylobacter coli
Source: PLoS One. 2022 Mar 31;17(3):e0265585. doi: 10.1371/journal.pone.0265585 (PMC8970381; doi:10.1371/journal.pone.0265585)
Supplement: S3 Table — (PDF) [file pone.0265585.s003.pdf]

S3 Table. LOS types of *C. coli* complete (n=22) and draft sequences (n=542)

Complete sequences: LOS Class I

No.	Strain	Accession #	4	5	6	7	8	Gene*	Class	Host
1	YH502	CP018900.1	99/100	99/100	96/100	99/100	99/100	96/100	I	Retail chicken US

LOS Class II

No.	Strain	Accession #	4	5	6	7	8	9	10	11	12	Gene*	Class	Host
1	JL-CDD-LMH	NZ_KZ253957.1	100/100	99/100	99/100	99/100	100/100	99/99	99/100	99/100	99/100	100/100	II	Unknown, China
2	BP3183	CP017871.1	100/100	100/100	99/100	99/100	100/100	99/100	100/100	100/100	100/100	95/100	II	Chicken, US
3	WA333	CP017873.1	100/100	100/100	99/100	99/100	100/100	99/100	100/100	99/100	100/100	100/100	II	Chicken, US
4	JV20	GL405235.1	100/100	100/100	100/100	99/100	99/100	100/100	100/100	99/100	100/100	100/100	II	Unknown, US

LOS Class III

No.	Strain	Accession #	4	5	6	7	8	9	10	11	Gene*	Class	host
1	BG2108	CP017878.1	99/100	98/100	100/100	100/100	100/100	100/100	100/100	100/100	100/100	III	Chicken, US
2	RM2228	AAFL01000002	99/100	99/100	99/100	99/100	100/100	99/100	99/100	100/100	100/100	III	Unknown, US
3	YF2105	CP017865.1	99/100	98/100	100/100	100/100	100/100	100/100	100/100	100/100	100/100	III	Chicken, US

LOS Class V

No.	Strain	Accession#	4	5	6	7	8	9	10	11/11'	12	13	14	15	Gene*	Class	Host
1	RM1875	CP007183.1	99/100	99/100	100/100	99/100	99/100	99/100	96/100	99/100	100/100	93/99	95/100	98/99	99/100	V	Unknown, US
2	ZV1224	CP017875.1	99/100	99/100			99/100	100/100	100/100	99/100	99/100	98/100	96/100	99/99		V with a pseudo gene	Pork, US

LOS Class VI

No.	Strain	Accession#	4	5	6	7	8	9	10	11	12	31	32	14	15	Class	Host
1	RM5611	CP007179.1	100/100	100/100	100/100	100/100	100/100	100/100	100/100	100/100	100/100	100/100	100/100	100/100	100/100	VI	Unknown, US
2	HC2-48	CP013034.1	100/100	100/100	100/100	100/100	100/100	100/100	100/100	100/100	100/100	100/100	100/100	100/100	100/100	VI	Beef, US
3	CF2-75	CP013036.1	100/100	100/100	100/100	100/100	100/100	100/100	100/100	100/100	100/100	100/100	100/100	100/100	100/100	VI	Beef, US

LOS Class VII

No.	Strain	Accession#	4	5	6	7	8	9	10	11	33	34	35	36	13	14	15	Class	Host
1	OR12	NZ_CP019977.1	99/100	99/100	100/100	99/100	100/100	100/100	100/100	100/100	100/100	99/100	99/100	100/100	100/100	98/100	99/100	VII	Chicken, UK
2	CFSAN032805	CP023545.1	99/100	99/100	100/100	99/100	100/100	100/100	100/100	100/100	100/100	99/100	99/100	100/100	100/100	98/100	99/100	VII	Chicken, US
3	CO2-160	CP013032.1	99/100	99/100	100/100	99/100	89/99	94/99	87/94							94/98	99/100	VII	Beef, US
4	K7	NZ_KI639691.1	99/100	99/100	100/100	99/100	100/100	100/100	100/100	100/100	100/100	99/100	99/100	100/100	100/100	98/100	99/100	VII	Unknown, UK

LOS Class VIII

No.	Strain	Accession #	4	5	6	7	8	9	10	11	12	13	14	15	16	17	Gene*	Class	Host
1	CVM N29710	NC_022347.1	100/100	100/100	100/100	100/100	100/100	100/100	100/100	99/100	100/100	100/100	100/100	100/100	100/100	100/100	100/100	VIII	Chicken, US
2	15-537360	NC_022660.1	99/100	100/100	100/100	100/100	100/100	100/100	100/100	100/100	100/100	100/100	100/100	100/100	100/100	99/100	94/100	VIII	Human, UK
3	FB1	CP011015.1	100/100	100/100	100/100	100/100	100/100	100/100	100/100	100/100	100/100	100/100	100/100	100/100	100/100	100/100	100/100	VIII	Human, UK
4	YH501	CP015528.1	100/100	100/100	100/100	100/100	100/100	100/100	100/100	100/100	100/100	100/100	99/100	100/100	100/100	100/100	100/100	VIII	Chicken, US
5	CVM 41957	NZ_JAJZ01000000	100/100	100/100	100/100	100/100	100/100	100/100	100/100	100/100	100/100	100/100	100/100	100/100	100/100	100/100	100/100	VIII	Human, US

Draft sequences:

LOS Class I

No.	Strain	Accession #	Contig #	4	5	6	7	Gene*	8	Class	Host
1	86119	AIMU01000000	31	99/100	99/100	99/100	99/100		100/100	I	Chicken US
2	OXC6372	CULY01000000	2	99/100	99/100	99/100	99/100	99/100	99/100	I	Faeces UK
3	OXC6276	CUII01000000	1	99/100	99/100	99/100	100/100	99/100	99/100	I	Faeces UK
4	OXC6378	CUMG01000000	1	99/100	99/100	99/100	99/100	99/100	99/100	I	Faeces UK
5	OXC6253	CUIX01000000	1	99/100	99/100	95/100	96/100		99/100	I	Faeces UK
6	CVM N44721	LBEL01000000	6	99/100	99/100	95/100	96/100	96/100	98/100	I	Breast chicken, US
7	CVM N46876	LBEJ01000000	4	99/100	99/100	96/100	96/100	96/100	98/100	I	Breast chicken, US
8	CVM N45714F	LBDV01000000	2	100/100	99/100	96/100	99/100		97/100	I	Turkey, US
9	CVM 41945	JAJP01000000	8	99/100	99/100	99/100	100/100		99/100	I	Humans, US
10	CVM 41970	JAJH01000000	11			99/100	99/100	96/100	97/100	I	Humans, US
11	CVM N26697	JOUO01000000	2	99/100	99/100	99/100	100/100	96/100	98/100	I	Chicken, US
12	OXC6258	CUKZ01000000	3	99/100	99/100	95/100	99/100	100/100	98/100	I	FAECES, UK
13	CVM N18323	JOUJ01000000	1	99/100	99/100	97/100	99/100	96/100	99/100	I	Chicken, US
14	CVM N26070	JOUN01000000	3	99/100	99/100	97/100	99/100	96/100	99/100	I	Turkey, US
15	H103060185	FBAG01000000	27	99/100	99/100	99/100	100/100	96/100	97/100	I	Environmental WATER, UK
16	P604D	FBLZ01000000	16	99/100	99/100	99/100	99/100	96/100	97/100	I	Soil, UK
17	SS_2357	FBEC01000000	13	99/100	99/100	99/100	100/100	97/100	100/100	I	Chicken, UK
18	H093100604	FBQF01000000	19	99/100	99/100	99/100	99/100	96/100	97/100	I	Environmental WATER, UK
19	H043880651	FBNR01000000	23	99/100	99/100	99/100	100/100	96/100	95/100	I	Human, UK
20	H063800417	FBQI01000000	11	99/100	98/100	99/100	99/100	98/100	97/100	I	Environmental WATER, UK
21	H102520382	FAZD01000000	38	99/100	99/100	99/100	99/100	97/100	100/100	I	Environmental WATER, UK
22	H140200373	FBLY01000000	15	99/100	99/100	99/100	100/100	98/100	97/100	I	Human, UK
23	UNOR383B	FBJO01000000	19	99/100	99/100	99/100	100/100	98/100	97/100	I	Chicken, UK
24	UNF5421c	FBMG01000000	25	99/100	99/100	99/100	99/100	96/100	97/100	I	Chicken, UK
25	H094280625	FBQL01000000	28	99/100	99/100	99/100	100/100	97/100	100/100	I	Environmental WATER, UK
26	H102380405	FAYB01000000	30	99/100	99/100	99/100	99/100		99/100	I	Environmental WATER, UK
27	H132580232	FBND01000000	77	99/100	99/100	95/100	96/100	100/100	100/100	I	Environmental WATER, UK
28	H124620276b	FBPL01000000	15	100/100	100/100	99/100	99/100	96/100	99/100	I	Human, UK
29	UNOR8693b	FBMI01000000	14	99/100	99/100	99/100	100/100	98/100	97/100	I	Chicken, UK
30	SS_2289	FBFR01000000	16			99/100	99/100		100/100	I	Chicken, UK
31	H142080277	FBPH01000000	21	99/100	99/100	99/100	100/100	96/100	97/100	I	Human, UK
32	11601	LKCS01000000	23	100/100	99/100	96/100	99/100	96/100	97/100	I	Turkey, US
33	CVM N23392	JOUM01000000	7			99/100	99/100	100/100	100/100	I	Chicken, US
34	BCW_6860	MJYP01000000	36	99/100	99/100	99/100	99/100	96/100	97/100	I	Faeces, US
35	BCW_6914	MJZH01000000	34	99/100	99/100	99/100	99/100	96/100	97/100	I	Faeces, US
36	BCW_6948	MJZK01000000	32	99/100	99/100	99/100	99/100	96/100	97/100	I	Faeces, US
37	BCW_6950	MJZL01000000	28	99/100	99/100	99/100	99/100	96/100	97/100	I	Faeces, US
38	BCW_6958	MJZS01000000	29	99/100	99/100	99/100	99/100	96/100	97/100	I	Faeces, US
39	BCW_7437	MJZY01000000	32	99/100	99/100	99/100	99/100	96/100	97/100	I	Faeces, US
40	BCW_5818	MKAO01000000	12	99/100	99/100	99/100	99/100	96/100	97/100	I	Faeces, US
41	BCW_6946	MKEX01000000	18	99/100	99/100	99/100	99/100	96/100	97/100	I	Monkey, US
42	OXC6476	CUPT01000000	2	99/100	99/100	99/100	99/100	96/100	98/100	I	Faeces, UK
43	RC282	CYRA01000000	77			99/100	99/100	100/100	99/100	I	Supermarket, UK
44	SH-CCD11C671	LISD01000000	4	93/100	99/100	99/100	100/100	96/100	99/100	I	Human, china
45	SH-CCHF12C088	LISE01000000	5	93/100	99/100	99/100	100/100	96/100	99/100	I	Chicken, china
46	SH-CCD12C100	LISF01000000	1	93/100	99/100	99/100	99/100	96/100	99/100	I	Human, china
47	BCW_4453	MJWB01000000	34	99/100	99/100	99/100	99/100	96/100	97/100	I	Faeces, US
48	BCW_4457	MJWC01000000	35	99/100	99/100	99/100	99/100	96/100	97/100	I	Faeces, US
49	BCW_5916	MJWD01000000	32	99/100	99/100	99/100	99/100	96/100	97/100	I	Faeces, US
50	BCW_5914	MJYL01000000	36	99/100	99/100	99/100	99/100	96/100	97/100	I	Faeces, US
51	6067	LKCQ01000000	14	100/100	100/100	96/100	99/100	96/100	97/100	I	Turkey house water, US
52	BFR-CA-9557	CP011777.1		100/100	100/100	99/100	100/100	96/100	99/100	I	Chicken, Germany
53	RC182	CYQT01000000	57	99/100	99/100	99/100	99/100	100/100	99/100	I	Supermarket, UK
54	RC096	CYQI01000000	56	99/100	99/100	99/100	99/100	100/100	99/100	I	Supermarket, UK

55	RC264	CYQW01000000	106			99/100	99/100		99/100	I	Supermarket, UK
56	RC285	CYRE01000000	27	99/100	99/100	99/100	99/100	100/100	99/100	I	Supermarket, UK
57	RC126	CYQM01000000	11			99/93	99/100	100//100	99/100	I	Supermarket, UK
58	RC387	CYRK01000000	7	99/100	99/100	99/100	99/100	100/100	99/100	I	Supermarket, UK
59	RC127	CYQN01000000	85	99/100	99/100	98/100	99/100	99/100	99/100	I	Supermarket, UK
60	RC037	CYQD01000000	27			99/94	100/100	96/100	97/100	I	Supermarket, UK
61	RC289	CYRG01000000	88	99/100	99/100	99/100	99/100	100/100	99/100	I	Supermarket, UK
62	RC415	CYRO01000000	3,81			99/86	99/100	100/100		I	Supermarket, UK
63	RC038	CYQE01000000	37	99/100	99/100	99/100	100/100	96/100	97/100	I	Supermarket, UK
64	RC428	CYRP01000000	6	99/100	99/100	99/100	99/100	100/100	99/100	I	Supermarket, UK
65	RC116	CYQL01000000	82, 91	99/100	99/100	99/100	99/100	100/100	98/90	I	Supermarket, UK
66	RC043	CYQF01000000	3	99/100	99/100	99/100	99/100	100/100	99/100	I	Supermarket, UK
67	RC281	CYQZ01000000	38			99/100	99/100	100/100	99/100	I	Supermarket, UK
68	RC284	CYRD01000000	73	99/100	99/100	99/100	99/100	100/100	99/100	I	Supermarket, UK
69	SH-CCD12C136	JXTU01000000	4	93/100	99/100	99/100	100/100	96/100	99/100	I	Human, China

LOS Class II

No.	Strain	Accession #	Contig#	4	5	6	7	8	9	10	11	12	Gene*	Class	Host
1	H8	AINU01000000	4, 27	100/100	100/100	99/100	99/100	100/100	99/100	100/100	99/100	99/100	92/100	II	Human, Switzerland
2	OXC6523	CURT01000000	1	99/100	100/100	100/100	99/100	100/100	100/100	99/100	100/100	100/100	91/100	II	Faeces, UK
3	OXC6442	CUOV01000000	1	100/100	100/100	99/100	99/100	100/100	99/100	99/100	99/100	99/100	99/100	II	Faeces, UK
4	OXC6443	CUOW01000000	1	100/100	100/100	99/100	99/100	100/100	99/100	99/100	99/100	99/100	99/100	II	Faeces, UK
5	OXC6537	CUSJ01000000	1	100/100	100/100	99/100	99/100	100/100	99/100	100/100	100/100	100/100	100/100	II	Faeces, UK
6	COL B1-266	LKIV01000000	274	100/100	100/100	99/100	99/100	100/100	99/100	100/100	100/100	100/100		II	Animal, Colombia
7	CVM N7464	JOVA01000000	1	100/84	100/100	99/100	99/100	100/100	99/100	100/100	100/100	100/100	93/100	II	Chicken, US
8	CVM N6388	JOUY01000000	4,15	100/100	100/100	99/100	99/100	100/100	99/100	100/100	100/100	99/100	100/100	II	Chicken, US
9	CVM N7454	JOUZ01000000	10	100/100	100/100	99/100	99/100	100/100	99/100	100/100	99/100			II	Chicken, US
10	CVM N9036	JOVE01000000	6, 9, 36	100/100	100/100	99/100	99/100	100/100	100/100	100/100	93/100	100/100	100/100	II	Pork, US
11	UNAJL222	FBIG01000000	25, 43	100/100	100/100	100/100	99/100	100/100	100/100	100/100	100/100	100/100	100/100	II	Pig, UK
12	H092660305	FBNL01000000	36	99/100	100/100	99/100	99/100	100/100	99/100	100/100	100/100	100/100	100/100	II	Human, UK
13	CCN349	FBIX01000000	28	99/100	100/100	99/100	99/100	100/100	99/100	100/100	100/100	99/100	91/99	II	Chicken, UK
14	UNNQFC9	FBLR01000000	19	99/100	100/100	99/100	99/100	100/100	99/100	99/100	100/100	100/100	95/99	II	Duck, UK
15	H060260417	FAXS01000000	11	100/100	100/100	99/100	99/100	100/100	99/100	99/100	100/100	100/90		II	Human, UK
16	UNOR581A	FBHW01000000	29	99/100	100/100	99/100	99/100	100/100	99/100	99/100	100/100	100/100	95/99	II	Chicken, UK
17	H044660164	FBKS01000000	10	100/100	100/100	100/100	99/100	100/100	100/100	100/100	100/100	100/100	100/100	II	Human, UK
18	H063900401	FBKM01000000	26	99/100	100/100	99/100	100/100	100/100	100/100	100/100	100/100	99/100	100/100	II	Human, UK
19	H072820535	FBBC01000000	15	100/100	100/100	99/100	99/100	100/100	99/100	100/100	100/100	100/100	100/100	II	Human, UK
20	SS_2356	FBED01000000	11	100/100	100/100	99/100	99/100	100/100	99/100	100/100	100/100	100/100	91/99	II	Chicken, UK
21	H102740168	FBOG01000000	25	99/100	100/100	100/100	99/100	100/100	100/100	99/100	100/100	100/100	91/100	II	Environmental water, UK
22	UNES9	FBHB01000000	26	100/100	100/100	99/100	99/100	100/100	99/100	100/100	100/100	100/100	100/100	II	Chicken, UK
23	H063540531	FBNJ01000000	16	99/100	100/100	100/100	99/100	100/100	100/100	99/100	100/100	100/100	91/100	II	Human, UK
24	H102240159	FBQQ01000000	233	100/100	100/100	99/100	99/100	100/100	99/100	100/100	100/100	100/100	100/100	II	Environmental water, UK
25	1535	FBGC01000000	7	100/100	100/100	100/100	99/100	100/100	100/100	100/100	100/100	100/100	100/100	II	Soil, UK
26	EC0349	FBJR01000000	23,27	99/100	100/100	99/100	99/100	100/100	99/100	100/100	100/100	99/100	91/99	II	Dairy, UK
27	H060280132	FBNZ01000000	24	100/100	100/100	100/100	99/100	100/100	100/100	100/100	100/100	100/100	92/100	II	Human, UK
28	H051160594	FBPK01000000	67	100/100	100/100	99/100	99/100	100/100	99/100	100/100	100/100	100/100	100/100	II	Human, UK
29	H072820536	FBNS01000000	18	100/100	100/100	99/100	99/100	100/100	99/100	100/100	100/100	100/100	100/100	II	Human, UK
30	M1483PM	LQXL01000000	23	100/100	100/100	99/100	99/100	100/100	99/100	100/100	100/100	100/100	100/100	II	Chicken, Colombia
31	M1486PM	LQXK01000000	19	100/100	100/100	99/100	99/100	100/100	99/100	100/100	100/100	100/100	100/100	II	Retail store, Colombia
32	C15	NFPY01000000	99, 133	100/100	100/100	99/99	99/100	100/100	99/100	100/100	100/100	100/100	100/100	II	Faeces, Canada
33	5	NFQH01000000	62, 72	100/100	100/100	99/100	99/100	100/100	99/100	100/100	100/100	100/100	100/100	II	Faeces, Canada
34	3	NFQJ01000000	43, 50	100/100	100/100	99/97	99/100	100/100	99/100	100/100	100/100	100/100	100/100	II	Retail Chicken, Canada
35	OXC6460	CUPB01000000	2	100/100	100/100	99/100	99/100	100/100	99/100	100/100	100/100	100/100	100/100	II	Faeces, UK
36	OXC6386	CUMP01000000	1	100/100	100/100	99/100	99/100	100/100	99/100	100/100	100/100	100/100	99/100	II	Faeces, UK
37	OXC6559	CUSX01000000	1	100/100	100/100	99/100	99/100	100/100	99/100	99/100	100/100	100/100	100/100	II	Faeces, UK
38	SH-CCF11C627	LISC01000000	1	100/100	100/100	100/100	99/100	100/100	100/100	100/100	99/100	100/100	93/100	II	Chicken, China
39	BCW_5137	MKCC01000000	23	100/100	100/100	99/100	99/100	100/100	100/100	100/100	100/100	100/100	92/100	II	Human, US
40	ICDCCC-SHCCH11C314	JXAD01000000	1	100/100	100/100	100/100	99/100	99/100	100/100	100/100	99/100	100/100	93/100	II	Chicken, China
41	SH-CCH11C605	LISB01000000	1	100/100	100/100	100/100	99/100	99/100	100/100	100/100	99/100	100/100	93/100	II	Chicken, China
42	SH-CCH11C390	LISA01000000	1,6	100/100	100/100	100/100	99/100	99/100	100/100	100/100	99/100	100/100	93/100	II	Chicken, China
43	SH-CCH11C334	LIRZ01000000	1	100/100	100/100	100/100	99/100	99/100	100/100	100/100	99/100	100/100	93/100	II	Chicken, China

LOS Class III

No.	Strain	Accession #	Contig #	4	5	6	7	8	9	10	11	Gene*	Class	host
1	CVM41915	JAJM01000000	55	99/100	99/100	99/100	99/100	100/100	100/100	99/100	99/100	100/100	III	Human, US
2	OXC6263	CUHU01000000	1	99/100	99/100	100/100	99/100	100/100	99/100	99/100	100/100	100/100	III	Faeces, UK
3	OXC6447	CUOY01000000	1	99/100	100/100	100/100	99/100	100/100	100/100	100/100	100/100	100/100	III	Faeces, UK
4	OXC6513	CURI01000000	1	99/100	100/100	100/100	99/100	100/100	100/100	100/100	100/100	100/100	III	Faeces, UK
5	OXC6297	CUJD01000000	2	99/100	99/100	100/100	100/100	100/100	100/100	99/100	100/100	100/100	III	Faeces, UK
6	OXC6337	CUKW01000000	1	99/100	100/100	100/100	99/100	100/100	99/100	100/100	100/100	100/100	III	Faeces, UK
7	OXC6380	CUMH01000000	2	99/100	98/100	100/100	100/100	100/100	100/100	100/100	100/100	100/100	III	Faeces, UK
8	OXC6472	CUPQ01000000	1	99/100	100/100	100/100	99/100	100/100	100/100	100/100	100/100	100/100	III	Faeces, UK
9	OXC6471	CUPO01000000	1	99/100	100/100	100/100	99/100	100/100	100/100	100/100	100/100	100/100	III	Faeces, UK
10	OXC6428	CUOH01000000	1	99/100	100/100	100/100	99/100	100/100	97/100	100/100	100/100	100/100	III	Faeces, UK
11	OXC6308	CUJQ01000000	1	99/100	100/100	100/100	99/100	100/100	100/100	100/100	100/100	100/100	III	Faeces, UK
12	OXC6267	CUHY01000000	1	99/100	100/100	100/100	100/100	100/100	97/100	100/100	100/100	100/100	III	Faeces, UK
13	CVM 41898	JAJK01000000	2, 66, 86, 85, 102	99/100	100/100		99/100	100/100	100/100	99/100	100/100	100/100	III	Human, US
14	CVM N14784	JOVU01000000	37	99/100	98/100	100/100	100/100	100/100	100/100	100/100	100/100	100/100	III	Chicken, US
15	CVM N23169	JOUL01000000	15	99/100	100/100	99/100	99/100	100/100	100/100	99/100	99/100	100/100	III	Chicken, US
16	CVM N26699	JOUP01000000	5	99/100	98/100	100/100	100/100	100/100	100/100	100/100	100/100	100/100	III	Chicken, US
17	CVM N462	JOUU01000000	11	99/100	98/100	100/100	100/100	100/100	100/100	100/100	100/100	100/100	III	Chicken, US
18	CVM N9077	JOVF01000000	11	99/100	100/100	99/100	100/100	100/100	100/100	99/100	99/100	100/100	III	Chicken, US
19	CVM N3508	JOUS01000000	16	99/100	99/100	99/100	99/100	100/100	99/100	99/100	100/100	100/100	III	Chicken, US
20	CVM N9093	JOVG01000000	7, 75, 90	99/100	98/100	100/100	100/100	100/100		100/100	100/100	100/100	III	Chicken, US
21	CVM N6401	JOVZ01000000	11	99/100	99/100	100/100	99/100	100/100	100/100	99/100	99/100	100/100	III	Chicken, US
22	CCN153	FBHE01000000	20	99/100	100/100	100/100	99/100	100/100	100/100	100/100	100/100	100/100	III	Chicken, UK
23	P604B	FBLL01000000	21	99/100	100/100	100/100	99/100	100/100	100/100	100/100	100/100	100/100	III	Soil, UK
24	EC3298	FBJM01000000	13	99/100	100/100	100/100	99/100	100/100	100/100	100/100	100/100	100/100	III	Farm environment, UK
25	EC3529	FAZB01000000	8	99/100	100/100	100/100	99/100	100/100	100/100	100/100	100/100	100/100	III	Farm environment, UK
26	EC3357	FBJD01000000	20	99/100	100/100	100/100	99/100	100/100	100/100	100/100	100/100	100/100	III	Farm environment, UK
27	UNOR13691b	FBMF01000000	5	99/100	100/100	100/100	99/100	100/100	100/100	100/100	100/100	100/100	III	Chicken, UK
28	EC3505	FBBL01000000	19	99/100	100/100	100/100	99/100	100/100	100/100	100/100	100/100	100/100	III	Farm environment, UK
29	EC3619	FAZR01000000	2	99/100	100/100	100/100	99/100	100/100	100/100	100/100	100/100	100/100	III	Farm environment, UK
30	EC6049	FBFE01000000	17	99/100	100/100	100/100	99/100	100/100	100/100	100/100	100/100	100/100	III	Farm environment, UK
31	CCN26	FBMP01000000	9	99/100	100/100	100/100	99/100	100/100	100/100	100/100	100/100	100/100	III	Farm environment, UK
32	UNQMCIIS18a	FAYE01000000	5	99/100	100/100	100/100	99/100	100/100	100/100	100/100	100/100	100/100	III	Human, UK
33	CCN181	FBJE01000000	29	99/100	100/100	100/100	99/100	100/100	100/100	100/100	100/100	100/100	III	Farm environment, UK
34	EC3879	FAZZ01000000	19	99/100	100/100	100/100	99/100	100/100	100/100	100/100	100/100	100/100	III	Farm environment, UK
35	EC6304	FBCC01000000	18	99/100	100/100	100/100	99/100	100/100	100/100	100/100	100/100	100/100	III	Farm environment, UK
36	EC4194	FAZJ01000000	21	99/100	100/100	100/100	99/100	100/100	100/100	100/100	100/100	100/100	III	Farm environment, UK
37	EC3501	FAYH01000000	22	99/100	100/100	100/100	99/100	100/100	100/100	100/100	100/100	100/100	III	Farm environment, UK
38	EC3693	FBAH01000000	17	99/100	100/100	100/100	99/100	100/100	100/100	100/100	100/100	100/100	III	Farm environment, UK
39	EC3615	FAYJ01000000	3	99/100	100/100	100/100	99/100	100/100	100/100	100/100	100/100	100/100	III	Farm environment, UK
40	EC5259	FBBO01000000	8	99/100	100/100	100/100	99/100	100/100	100/100	100/100	100/100	100/100	III	Farm environment, UK
41	EC4473	FBCJ01000000	19	99/100	100/100	100/100	99/100	100/100	100/100	100/100	100/100	100/100	III	Farm environment, UK
42	EC3623	FBDC01000000	19	99/100	100/100	100/100	99/100	100/100	100/100	100/100	100/100	100/100	III	Dairy farm, Water, UK
43	EC3365	FBGZ01000000	12	99/100	100/100	100/100	99/100	100/100	100/100	100/100	100/100	100/100	III	Dairy farm, Water, UK
44	EC3533	FBAE01000000	1	99/100	100/100	100/100	99/100	100/100	100/100	100/100	100/100	100/100	III	Dairy farm, Water, UK
45	EC6299	FBDF01000000	23	99/100	99/100	100/100	99/100	100/100	100/100	100/100	100/100	100/100	III	Dairy Farm, Faeces, UK
46	EC5850	FBBX01000000	4	99/100	100/100	100/100	99/100	100/100	100/100	100/100	100/100	100/100	III	Farm environment, UK
47	EC6124	FBCE01000000	3	99/100	100/100	100/100	99/100	100/100	100/100	100/100	100/100	100/100	III	Dairy Farm, Faeces, UK
48	H133040289	FBBI01000000	46	99/100	100/100	100/100	99/100	100/100	100/100	100/100	100/100	100/100	III	Environmental water, UK
49	CCN265	FBJH01000000	10	99/100	100/100	100/100	99/100	100/100	100/100	100/100	100/100	100/100	III	Poultry farm , UK
50	EC3782	FBAD01000000	3	99/100	100/100	100/100	99/100	100/100	100/100	100/100	100/100	100/100	III	Dairy farm, Water, UK
51	EC5923	FBBY01000000	4	99/100	100/100	100/100	99/100	100/100	100/100	100/100	100/100	100/100	III	Dairy farm, Water, UK
52	H081940749	FBNA01000000	12	99/100	100/100	100/100	99/100	100/100	100/100	100/100	100/100	100/100	III	Environmental water, UK
53	UNOR10622c	FBMD01000000	49	99/100	99/100	100/100	99/100	100/100	100/100	99/100	100/100	100/100	III	Chicken, UK
54	EC3478	FBHJ01000000	4	99/100	100/100	100/100	99/100	100/100	100/100	100/100	100/100	100/100	III	Dairy farm, Water, UK
55	EC4593	FBDH01000000	21	99/100	100/100	100/100	99/100	100/100	100/100	100/100	100/100	100/100	III	Farm environment, UK
56	EC4910	FBFH01000000	14	99/100	100/100	100/100	99/100	100/100	100/100	100/100	100/100	100/100	III	Farm environment, UK

57	EC3537	FBBA01000000	2	99/100	100/100	100/100	99/100	100/100	100/100	100/100	100/100	100/100	III	Dairy farm, Water, UK
58	EC3849	FAXR01000000	18	99/100	100/100	100/100	99/100	100/100	100/100	100/100	100/100	100/100	III	Dairy farm, Water, UK
59	CCN123	FBIO01000000	16	99/100	100/100	100/100	99/100	100/100	100/100	100/100	100/100	100/100	III	Poultry, UK
60	EC3940	FBBK01000000	7	99/100	100/100	100/100	99/100	100/100	100/100	100/100	99/100	100/100	III	Farm environment, UK
61	P474A	FBKG01000000	18	99/100	100/100	100/100	99/100	100/100	100/100	100/100	99/100	100/100	III	Soil, UK
62	EC3146	FBGF01000000	18	99/100	100/100	100/100	99/100	100/100	100/100	100/100	99/100	100/100	III	Farm environment, UK
63	EC5721	FBFG01000000	16	99/100	100/100	100/100	99/100	100/100	100/100	100/100	99/100	100/100	III	Dairy Farm, Faeces, UK
64	BRIS1041X	FBIH01000000	15	99/100	99/100	100/100	100/100	100/100	99/100	99/100	99/100	100/100	III	Soil, UK
65	EC3627	FBAA01000000	4	99/100	100/100	100/100	99/100	100/100	100/100	100/100	99/100	100/100	III	Dairy farm, Water, UK
66	H103480422	FBQB01000000	13	99/100	99/100	100/100	100/100	100/100	100/100	100/100	99/100	100/100	III	Environmental Water, UK
67	UNF383D	FBGP01000000	49	99/100	100/100	100/100	99/100	100/100	100/100	100/100	99/100	100/100	III	Chicken, UK
68	EC3511	FBHI01000000	15	99/100	100/100	100/100	99/100	100/100	100/100	100/100	99/100	100/100	III	Farm environment, UK
69	EC3575	FBAR01000000	19	99/100	100/100	100/100	99/100	100/100	100/100	100/100	99/100	100/100	III	Farm environment, UK
70	EC3525	FAYA01000000	2	99/100	100/100	100/100	99/100	100/100	100/100	100/100	99/100	100/100	III	Dairy farm, Water, UK
71	EC4297	FBEV01000000	17	99/100	100/100	100/100	99/100	100/100	100/100	100/100	99/100	100/100	III	Dairy Farm, Faeces, UK
72	EC3952	FBCP01000000	2	99/100	100/100	100/100	99/100	100/100	100/100	100/100	99/100	100/100	III	Farm environment, UK
73	EC3521	FBAN01000000	15	99/100	100/100	100/100	99/100	100/100	100/100	100/100	99/100	100/100	III	Dairy farm, Water, UK
74	H110420358	FAYG01000000	40	99/100	100/100	100/100	99/100	100/100	100/100	100/100	99/100	93/100	III	Environmental Water, UK
75	EC3389	FAZG01000000	5	99/100	100/100	100/100	99/100	100/100	100/100	100/100	99/100	100/100	III	Dairy farm, Water, UK
76	H132760749	FAYP01000000	5	99/100	99/100	100/100	99/100	100/100	100/100	100/100	99/100	100/100	III	Environmental Water, UK
77	EC4238	FBFF01000000	18	99/100	100/100	100/100	99/100	100/100	100/100	100/100	99/100	100/100	III	Environmental Water, UK
78	CCN288	FBGL01000000	22	99/100	100/100	100/100	99/100	100/100	100/100	100/100	99/100	100/100	III	Poultry farm water, UK
79	EC6168	FBBM01000000	19	99/100	100/100	100/100	99/100	100/100	100/100	100/100	99/100	100/100	III	Dairy Farm, Faeces, UK
80	EC3786	FBBH01000000	18	99/100	100/100	100/100	99/100	100/100	100/100	100/100	99/100	100/100	III	Dairy farm, Water, UK
81	EC4768	FBDG01000000	22	99/100	100/100	100/100	99/100	100/100	100/100	100/100	99/100	100/100	III	Dairy Farm, Faeces, UK
82	EC3631	FBAQ01000000	20	99/100	100/100	100/100	99/100	100/100	100/100	100/100	99/100	100/100	III	Dairy farm, Water, UK
83	EC3731	FAZA01000000	3	99/100	100/100	100/100	99/100	100/100	100/100	100/100	99/100	100/100	III	Dairy farm, Water, UK
84	EC4098	FBEZ01000000	16	99/100	100/100	100/100	99/100	100/100	100/100	100/100	99/100	100/100	III	Dairy farm, Water, UK
85	P546D	FBKR01000000	16	99/100	100/100	100/100	99/100	100/100	100/100	100/100	99/100	100/100	III	Soil, UK
86	H091320788	FBPN01000000	7	99/100	100/100	100/100	99/100	100/100	100/100	100/100	99/100	100/100	III	Environmental Water, UK
87	CCN154	FBHM01000000	16	99/100	100/100	100/100	99/100	100/100	100/100	100/100	99/100	100/100	III	Poultry Environment, UK
88	H140660843	FBJG01000000	14	99/100	100/100	100/100	99/100	100/100	100/100	100/100	99/100	100/100	III	Human, UK
89	EC4722	FBDA01000000	3	99/100	100/100	100/100	99/100	100/100	100/100	100/100	99/100	100/100	III	Environmental Water, UK
90	CCN71	FBIV01000000	3	99/100	100/100	100/100	99/100	100/100	100/100	100/100	100/100	100/100	III	Poultry Environment, UK
91	EC6158	FBBN01000000	18	99/100	100/100	100/100	99/100	100/100	100/100	100/100	100/100	100/100	III	Dairy Farm, Faeces, UK
92	CCN292	FBJS01000000	11	99/100	100/100	100/100	99/100	100/100	100/100	100/100	99/100	100/100	III	Poultry Environment, Water, UK
93	EC5240a	FBBG01000000	3	99/100	100/100	100/100	99/100	100/100	100/100	100/100	99/100	100/100	III	Dairy Farm, Faeces, UK
94	EC3607	FAYZ01000000	14	99/100	100/100	100/100	99/100	100/100	100/100	100/100	99/100	100/100	III	Dairy farm, Water, UK
95	H065100499	FBKW01000000	23	99/100	100/100	100/100	99/100	100/100	100/100	100/100	99/100	100/100	III	Environmental Water, UK
96	EC4102	FBDR01000000	15	99/100	100/100	100/100	99/100	100/100	100/100	100/100	99/100	100/100	III	Dairy farm, Water, UK
97	EC3390	FBHC01000000	11	99/100	100/100	100/100	99/100	100/100	100/100	100/100	99/100	100/100	III	Dairy farm, Water, UK
98	H132580239	FBQD01000000	25	99/100	100/100	100/100	99/100	100/100	100/100	100/100	100/100	100/100	III	Human, UK
99	EC5246a	FBBW01000000	5	99/100	100/100	100/100	99/100	100/100	100/100	100/100	99/100	100/100	III	Dairy Farm, Faeces, UK
100	H132580228	FBAS01000000	67	99/100	100/100	100/100	99/100	100/100	100/100	100/100	100/100	100/100	III	Environmental Water, UK
101	EC3756	FAZK01000000	18	99/100	100/100	100/100	99/100	100/100	100/100	100/100	99/100	100/100	III	Farm environment, UK
102	EC3348	FBBD01000000	21	99/100	100/100	100/100	99/100	100/100	100/100	100/100	99/100	100/100	III	Dairy farm, Water, UK
103	EC5183	FBDO01000000	17	99/100	100/100	100/100	99/100	100/100	100/100	100/100	99/100	100/100	III	Dairy Farm, Faeces, UK
104	H073900238	FBOT01000000	44	99/100	100/100	100/100	99/100	100/100	100/100	100/100	99/100	100/100	III	Environmental Water, UK
105	EC6122	FBFM01000000	17	99/100	100/100	100/100	99/100	100/100	100/100	100/100	99/100	100/100	III	Dairy Farm, Faeces, UK
106	EC5240b	FBDI01000000	21	99/100	100/100	100/100	99/100	100/100	100/100	100/100	99/100	100/100	III	Dairy Farm, Faeces, UK
107	CCN293	FBIY01000000	9	99/100	100/100	100/100	99/100	100/100	100/100	100/100	99/100	100/100	III	Poultry Environment, Water, UK
108	EC3361	FBIC01000000	13	99/100	100/100	100/100	99/100	100/100	100/100	100/100	99/100	100/100	III	Dairy farm, Water, UK
109	EC5679	FBBR01000000	3	99/100	100/100	100/100	99/100	100/100	100/100	100/100	99/100	100/100	III	Dairy Farm, Faeces, UK
110	EC4709	FBCD01000000	20	99/100	100/100	100/100	99/100	100/100	100/100	100/100	99/100	100/100	III	Farm environment, UK
111	EC4214	FAXQ01000000	1	99/100	100/100	100/100	99/100	100/100	100/100	100/100	99/100	100/100	III	Dairy Farm, Faeces, UK
112	H084040382b	FBPU01000000	8	99/100	100/100	100/100	99/100	100/100	100/100	100/100	99/100	100/100	III	Human, UK
113	H132940658	FBPY01000000	23	99/100	100/100	99/100	99/100	100/100	100/100	100/100	99/100	100/100	III	Human, UK
114	EC4250	FBCS01000000	18	99/100	100/100	100/100	99/100	100/100	100/100	100/100	100/100	100/100	III	Dairy farm, Water, UK
115	EC5520	FBCO01000000	3	99/100	100/100	100/100	99/100	100/100	100/100	100/100	100/100	100/100	III	Dairy Farm, Faeces, UK
116	EC4393	FBAY01000000	3	99/100	100/100	100/100	99/100	100/100	100/100	100/100	100/100	100/100	III	Dairy Farm, Faeces, UK
117	EC3956	FAZP01000000	19	99/100	100/100	100/100	99/100	100/100	100/100	100/100	100/100	100/100	III	Farm environment, UK
118	EC5726	FAYM01000000	16	99/100	100/100	100/100	99/100	100/100	100/100	100/100	100/100	100/100	III	Dairy Farm, Faeces, UK
119	NCTC12570	FBHN01000000	15	-	99/100	100/100	100/100	100/100	100/100	100/100	100/100	100/100	III	Unknown
120	H120880380	FAYN01000000	44	99/100	100/100	100/100	100/100	100/100	100/100	100/100	100/100	100/100	III	Human, UK
121	P474C	FBKF01000000	9, 12	-	100/100	100/100	99/100	100/100	100/100	100/100	100/100	100/100	III	Soil, UK

122	EC4258	FBCM01000000	19	99/100	100/100	100/100	99/100	100/100	100/100	100/100	100/100	100/100	III	Dairy farm, Water, UK
123	EC5992	FBDL01000000	20	99/100	100/100	100/100	99/100	100/100	100/100	100/100	100/100	100/100	III	Dairy Farm, Faeces, UK
124	EC5693	FBFN01000000	17	99/100	100/100	100/100	99/100	100/100	100/100	99/100	100/100	100/100	III	Dairy Farm, Faeces, UK
125	H130500174	FBQH01000000	39	99/100	99/100	100/100	100/100	100/100	100/100	100/100	100/100	100/100	III	Human, UK
126	CCN56	FBIU01000000	15	99/100	100/100	100/100	99/100	100/100	100/100	100/100	100/100	100/100	III	Poultry Farm, Faeces, UK
127	SWAN392	FBLQ01000000	17	99/100	100/100	100/100	99/100	100/100	100/100	100/100	100/100	100/100	III	Duck, UK
128	EC3721	FBDP01000000	18	99/100	100/100	100/100	99/100	100/100	100/100	100/100	100/100	100/100	III	Dairy farm, Water, UK
129	EC4690	FBDB01000000	3	99/100	100/100	100/100	99/100	100/100	100/100	100/100	100/100	100/100	III	Farm environment, UK
130	CCN60	FBGI01000000	5	99/100	100/100	100/100	99/100	100/100	100/100	100/100	100/100	100/100	III	Poultry Farm, Faeces, UK
131	EC4287	FBCK01000000	6	99/100	100/100	100/100	99/100	100/100	100/100	100/100	100/100	100/100	III	Dairy Farm, Faeces, UK
132	EC4946	FBCR01000000	18	99/100	100/100	100/100	99/100	100/100	100/100	100/100	100/100	100/100	III	Dairy Farm, Faeces, UK
133	EC5479	FAYI01000000	21	99/100	100/100	100/100	99/100	100/100	100/100	100/100	100/100	100/100	III	Dairy Farm, Faeces, UK
134	EC5841	FBBP01000000	4	99/100	100/100	100/100	99/100	100/100	100/100	100/100	100/100	100/100	III	Farm environment, UK
135	EC3689	FBDN01000000	22	99/100	100/100	100/100	99/100	100/100	100/100	100/100	100/100	100/100	III	Farm environment, UK
136	EC6173	FBCV01000000	26	99/100	100/100	100/100	99/100	100/100	100/100	100/100	100/100	100/100	III	Farm environment, UK
137	H140460193	FBMV01000000	9	99/100	100/100	100/100	99/100	100/100	100/100	100/100	100/100	100/100	III	Human, UK
138	BRISLC31-1	FBGO01000000	29	99/100	99/100	100/100	100/100	100/100	99/100	99/100	100/100	100/100	III	Dog, UK
139	EC4060	FAYU01000000	20	99/100	100/100	100/100	99/100	100/100	100/100	100/100	100/100	100/100	III	Farm environment, UK
140	EC3370	FAZT01000000	18	99/100	100/100	100/100	99/100	100/100	100/100	100/100	100/100	100/100	III	Dairy farm, Water, UK
141	EC5905	FBCF01000000	3	99/100	100/100	100/100	99/100	100/100	100/100	100/100	100/100	100/100	III	Farm environment, UK
142	EC4675	FBCY01000000	21	99/100	100/100	100/100	99/100	100/100	100/100	100/100	100/100	100/100	III	Dairy Farm, Faeces, UK
143	EC3385	FBIS01000000	15	99/100	100/100	100/100	99/100	100/100	100/100	100/100	100/100	100/100	III	Dairy farm, Water, UK
144	P568B	FAXX01000000	13	99/100	100/100	100/100	99/100	100/100	100/100	100/100	100/100	100/100	III	Soil, UK
145	H040680225	FAYW01000000	1	99/100	100/100	100/100	99/100	100/100	100/100	100/100	100/100	100/100	III	Human, UK
146	NCTC11438	FBMN01000000	17	99/100	100/100	100/100	99/100	100/100	100/100	100/100	100/100	100/100	III	Human, UK
147	CCN59	FBJF01000000	14	99/100	100/100	100/100	99/100	100/100	100/100	100/100	100/100	100/100	III	Poultry Farm, Faeces, UK
148	EC3727	FAZQ01000000	5	99/100	100/100	100/100	99/100	100/100	100/100	100/100	100/100	100/100	III	Dairy farm, Water, UK
149	EC4242	FBCW01000000	2	99/100	100/100	100/100	99/100	100/100	100/100	100/100	100/100	100/100	III	Dairy farm, Water, UK
150	EC3381	FBIN01000000	24	99/100	100/100	100/100	99/100	100/100	100/100	100/100	100/100	100/100	III	Dairy farm, Water, UK
151	H053280346	FBOY01000000	15	99/100	99/100	100/100	99/100	100/100	99/100	99/100	100/100	100/100	III	Human, UK
152	EC5531	FBBZ01000000	22	99/100	100/100	100/100	99/100	100/100	100/100	100/100	100/100	100/100	III	Dairy Farm, Faeces, UK
153	EC3397	FBCQ01000000	4	99/100	100/100	100/100	99/100	100/100	100/100	100/100	100/100	100/100	III	Dairy farm, Water, UK
154	EC3774	FBAJ01000000	7	99/100	100/100	100/100	99/100	100/100	100/100	100/100	100/100	100/100	III	Dairy farm, Water, UK
155	EC3373	FBGU01000000	7	99/100	100/100	100/100	99/100	100/100	100/100	100/100	100/100	100/100	III	Dairy farm, Water, UK
156	P635D	FBKB01000000	18	99/100	100/100	100/100	99/100	100/100	100/100	100/100	100/100	100/100	III	Soil, UK
157	EC3611	FBCL01000000	21	99/100	100/100	100/100	99/100	100/100	100/100	100/100	100/100	100/100	III	Farm environment, UK
158	H063800423	FBQU01000000	38	99/100	100/100	100/100	100/100	100/100	100/100	100/100	100/100	100/100	III	Environmental Water, UK
159	H092260569a	FBNX01000000	10	99/100	100/100	100/100	99/100	100/100	100/100	100/100	100/100	100/100	III	Environmental Water, UK
160	EC3400	FBAB01000000	18	99/100	100/100	100/100	99/100	100/100	100/100	100/100	100/100	100/100	III	Dairy farm, Water, UK
161	H073180384	FBJW01000000	15	99/100	100/100	100/100	99/100	100/100	100/100	100/100	100/100	100/100	III	Human, UK
162	EC4356	FBCI01000000	3	99/100	100/100	100/100	99/100	100/100	100/100	100/100	100/100	100/100	III	Farm environment, UK
163	EC3444	FBBE01000000	4	99/100	100/100	100/100	99/100	100/100	100/100	100/100	100/100	100/100	III	Farm environment, UK
164	P546A	FBNB01000000	23	99/100	100/100	100/100	99/100	100/100	100/100	100/100	100/100	100/100	III	Soil, UK
165	CCN119	FBIL01000000	21	99/100	100/100	100/100	99/100	100/100	100/100	100/100	100/100	100/100	III	Poultry Environmental, UK
166	EC3490	FBHP01000000	23	99/100	100/100	100/100	99/100	100/100	100/100	100/100	100/100	100/100	III	Dairy farm, Water, UK
167	EC4530	FBDD01000000	14	99/100	100/100	100/100	99/100	100/100	100/100	100/100	100/100	100/100	III	Dairy Farm, Faeces, UK
168	EC3344	FBJK01000000	3	99/100	100/100	100/100	99/100	100/100	100/100	100/100	100/100	100/100	III	Dairy farm, Water, UK
169	EC4462	FBCH01000000	3	99/100	100/100	100/100	99/100	100/100	100/100	100/100	100/100	100/100	III	Farm environment, UK
170	EC5338	FBFJ01000000	17	99/100	100/100	100/100	99/100	100/100	100/100	100/100	100/100	100/100	III	Dairy Farm, Faeces, UK
171	EC5604	FBBV01000000	21	99/100	100/100	100/100	99/100	100/100	100/100	100/100	100/100	100/100	III	Dairy Farm, Faeces, UK
172	H044040580	FAZN01000000	48	99/100	100/100	100/100	99/100	100/100	100/100	100/100	100/100	100/100	III	Human, UK
173	H111620356	FAYY01000000	3	99/100	100/100	100/100	99/100	100/100	100/100	100/100	100/100	100/100	III	Environmental Water, UK
174	EC4868	FBCB01000000	1	99/100	100/100	100/100	99/100	100/100	100/100	100/100	100/100	100/100	III	Dairy Farm, Faeces, UK
175	H062180535	FAZV01000000	1	99/100	100/100	100/100	99/100	100/100	100/100	100/100	100/100	100/100	III	Human, UK
176	P635A	FBKH01000000	18	99/100	100/100	100/100	99/100	100/100	100/100	100/100	100/100	100/100	III	Soil, UK
177	EC3705	FBCZ01000000	3	99/100	100/100	100/100	99/100	100/100	100/100	100/100	100/100	100/100	III	Farm environment, UK
178	EC5072	FBFL01000000	18	99/100	100/100	100/100	99/100	100/100	100/100	100/100	100/100	100/100	III	Dairy Farm, Faeces, UK
179	H114640463a	FBLE01000000	20	99/100	100/100	100/100	99/100	100/100	100/100	100/100	100/100	100/100	III	Human, UK
180	EC3326	FBAC01000000	2	99/100	100/100	100/100	99/100	100/100	100/100	100/100	100/100	100/100	III	Dairy farm, Water, UK
181	H053780444	FBBB01000000	19	99/100	100/100	100/100	99/100	100/100	100/100	100/100	100/100	100/100	III	Human, UK
182	EC4448	FBAZ01000000	6	99/100	100/100	100/100	99/100	100/100	100/100	100/100	100/100	100/100	III	Dairy Farm, Faeces, UK
183	EC4978	FBDJ01000000	20	99/100	100/100	100/100	99/100	100/100	100/100	100/100	100/100	100/100	III	Dairy farm, Water, UK
184	EC3735	FAXT01000000	7	99/100	100/100	100/100	99/100	100/100	100/100	100/100	100/100	100/100	III	Dairy farm, Water, UK
185	H132340486	FAZC01000000	3	99/100	100/100	100/100	99/100	100/100	100/100	100/100	100/100	100/100	III	Environmental Water, UK
186	CCN64	FBJU01000000	24	99/100	100/100	100/100	99/100	100/100	100/100	100/100	100/100	100/100	III	Poultry Environmental, UK
187	CCN72	FBIQ01000000	16	99/100	100/100	100/100	99/100	100/100	100/100	100/100	100/100	100/100	III	Poultry Environmental, UK

188	H072680465	FBOF01000000	10	99/100	100/100	100/100	99/100	100/100	100/100	100/100	100/100	100/100	III	Environmental Water, UK
189	EC3322	FBJQ01000000	3	99/100	100/100	100/100	99/100	100/100	100/100	100/100	100/100	100/100	III	Dairy farm, Water, UK
190	EC3725	FBAV01000000	16	99/100	100/100	100/100	99/100	100/100	100/100	100/100	100/100	100/100	III	Dairy farm, Water, UK
191	EC6047	FBCA01000000	2	99/100	100/100	100/100	99/100	100/100	100/100	100/100	100/100	100/100	III	Dairy Farm, Faeces, UK
192	EC5983	FBFC01000000	18	99/100	100/100	100/100	99/100	100/100	100/100	100/100	100/100	100/100	III	Dairy Farm, Faeces, UK
193	EC6016	FBBF01000000	14	99/100	100/100	100/100	99/100	100/100	100/100	100/100	100/100	100/100	III	Farm environment, UK
194	H132760142	FBOU01000000	73	99/100	99/100	100/100	100/100	100/100	99/100	99/100	100/100	100/100	III	Environmental Water, UK
195	H073700405	FBNF01000000	27	99/100	99/100	100/100	100/100	100/100	99/100	100/100	100/100	100/100	III	Environmental Water, UK
196	EC3356	FBJC01000000	7	99/100	100/100	100/100	99/100	100/100	100/100	100/100	100/100	100/100	III	Environmental Water, UK
197	EC4951	FBAX01000000	19	99/100	100/100	100/100	99/100	100/100	100/100	100/100	100/100	100/100	III	Dairy Farm, Faeces, UK
198	EC4383	FBCU01000000	18	99/100	100/100	100/100	99/100	100/100	100/100	100/100	100/100	100/100	III	Dairy Farm, Faeces, UK
199	EC4277	FBDE01000000	4	99/100	100/100	100/100	99/100	100/100	100/100	100/100	100/100	100/100	III	Dairy Farm, Faeces, UK
200	EC5246	FBDM01000000	3	99/100	100/100	100/100	99/100	100/100	100/100	100/100	100/100	100/100	III	Dairy Farm, Faeces, UK
201	CCN124	FBJT01000000	9	99/100	100/100	100/100	99/100	100/100	100/100	100/100	100/100	100/100	III	Poultry Environmental, UK
202	EC6203	FBBT01000000	10	99/100	100/100	100/100	99/100	100/100	100/100	100/100	100/100	100/100	III	Dairy Farm, Faeces, UK
203	EC3466	FBJJ01000000	20	99/100	100/100	100/100	99/100	100/100	100/100	100/100	100/100	100/100	III	Dairy farm, Water, UK
204	EC4808	FBFD01000000	1	99/100	100/100	100/100	99/100	100/100	100/100	100/100	100/100	100/100	III	Dairy Farm, Faeces, UK
205	EC5806	FBEY01000000	15	99/100	100/100	100/100	99/100	100/100	100/100	100/100	100/100	100/100	III	Dairy Farm, Faeces, UK
206	EC4685	FBDK01000000	1	99/100	100/100	100/100	99/100	100/100	100/100	100/100	100/100	100/100	III	Dairy Farm, Faeces, UK
207	EC3748	FAYT01000000	2	99/100	100/100	100/100	99/100	100/100	100/100	100/100	100/100	100/100	III	Farm environment, UK
208	EC4267	FBFB01000000	14	99/100	100/100	100/100	99/100	100/100	100/100	100/100	100/100	100/100	III	Dairy Farm, Faeces, UK
209	EC3369	FAYQ01000000	2	99/100	100/100	100/100	99/100	100/100	100/100	100/100	100/100	100/100	III	Dairy farm, Water, UK
210	EC3713	FBAU01000000	18	99/100	100/100	100/100	99/100	100/100	100/100	100/100	100/100	100/100	III	Dairy farm, Water, UK
211	EC3928	FBCX01000000	19	99/100	100/100	100/100	99/100	100/100	100/100	100/100	100/100	100/100	III	Farm environment, UK
212	EC3875	FAZU01000000	1	99/100	100/100	100/100	99/100	100/100	100/100	100/100	100/100	100/100	III	Farm environment, UK
213	EC5899	FBEO01000000	20	99/100	100/100	100/100	99/100	100/100	100/100	100/100	100/100	100/100	III	Dairy Farm, Faeces, UK
214	CCN55	FBMM01000000	21	99/100	100/100	100/100	99/100	100/100	100/100	100/100	100/100	100/100	III	Poultry Environmental, UK
215	EC5947	FBCN01000000	3	99/100	99/100	100/100	99/100	100/100	100/100	100/100	100/100	100/100	III	Dairy Farm, Faeces, UK
216	EC4956	FBFK01000000	14	99/100	100/100	100/100	99/100	100/100	100/100	100/100	100/100	100/100	III	Dairy Farm, Faeces, UK
217	EC3498	FBGS01000000	11	99/100	100/100	100/100	99/100	100/100	100/100	100/100	100/100	100/100	III	Dairy farm, Water, UK
218	EC4730	FBAO01000000	18	99/100	100/100	100/100	99/100	100/100	100/100	100/100	100/100	100/100	III	Dairy farm, Water, UK
219	EC3338	FAYS01000000	19	99/100	100/100	100/100	99/100	100/100	100/100	100/100	100/100	100/100	III	Dairy farm, Water, UK
220	H120880379	FAZX01000000	35	99/100	100/100	100/100	99/100	100/100	100/100	99/100	100/100	100/100	III	Human, UK
221	20A_420	MCFT01000000	1	99/100	100/100	100/100	100/100	100/100	100/100	100/100	100/100	100/100	III	Human, UK
222	OXC6504	CUQY01000000	1	99/100	100/100	100/100	99/100	100/100	97/100	100/100	100/100	100/100	III	Faeces, UK
223	OXC6474	CUPR01000000	1	99/100	100/100	100/100	99/100	100/100	100/100	100/100	100/100	100/100	III	Faeces, UK
224	OXC6312	CUJV01000000	1	99/100	100/100	100/100	99/100	100/100	100/100	100/100	100/100	100/100	III	Faeces, UK
225	U2	NFOA01000000	4	99/100	98/100	100/100	99/100	100/100	100/100	100/100	100/100	100/100	III	Retail Chicken, Canada
226	ZJ2013CCHD50	JXTV01000000	1	99/100	100/100	100/100	99/100	100/100	100/100	100/100	100/100	100/100	III	Chicken, China

LOS Class IV

No.	Strain	Accession #	Contig#	4	5	6	7	8	9	10	11	12	13	14	15	Class	Host
1	LMG9860	AINS01000000	6, 47	99/100	99/100	100/100	100/100	100/100	99/100	95/82	99/100	99/100	95/99	96/100	99/100	IV	Human, Canada
2	H56	AINW01000000	6	99/100	99/100	100/100	100/100	100/100	99/100	100/100	99/100	99/100	95/99	97/100	96/100	IV	Human, Switzerland
3	OXC6425	CUOB01000000	1	99/100	100/100	100/100	100/100	100/100	99/100	99/100	99/100	99/100	95/99	95/100	99/100	IV	Faeces, UK
4	CVM 41963	JAJR01000000	16				100/100	100/100	99/100	99/100	99/100	99/100	95/99	99/100	99/100	IV	Human, US
5	CVM N18725	JOUK01000000	7	100/100	100/100	99/100	100/100	100/100	100/100	100/100	99/100	99/100	99/100	96/100	99/100	IV	Chicken, US
6	CVM N287	JOVY01000000	5	99/100	99/100	100/100	100/100	100/100	99/100	100/100	99/100	99/100	95/99	95/100	98/100	IV	Pork, US
7	H044220458	FBAP01000000	39	99/100	99/100	100/100	100/100	100/100	99/100	96/100	99/100	99/100	100/100	96/100	99/100	IV	Human, UK
8	SS_2326	FBER01000000	13	99/100	99/100	100/100	100/100	100/100	99/100	100/100	99/100	99/100	95/99	96/100	99/100	IV	Chicken, UK
9	SS_2238	FBEP01000000	35	99/100	99/100	100/100	100/100	100/100	99/100	100/100	99/100	99/100	95/99	96/100	99/100	IV	Chicken, UK
10	SS_2226	FBEF01000000	44	99/100	99/100	100/100	100/100	100/100	99/100	100/100	99/100	99/100	95/99	96/100	99/100	IV	Chicken, UK
11	H121240507	FAZF01000000	27	99/100	99/100	100/100	100/100	99/100	99/100	99/100	99/100	99/100	95/99	96/100	99/100	IV	Human, UK
12	SS_2296	FBEQ01000000	8	99/100	99/100	100/100	100/100	100/100	99/100	100/100	99/100	99/100	95/99	96/100	99/100	IV	Chicken, UK
13	SS_2318	FBEE01000000	14	99/100	99/100	100/100	100/100	100/100	99/100	100/100	99/100	99/100	95/99	96/100	99/100	IV	Chicken, UK
14	H043940494	FBNH01000000	24	99/100	99/100	100/100	100/100	100/100	99/100	100/100	99/100	99/100	95/99	95/100	98/100	IV	Human, UK
15	SS_2291	FBEN01000000	11	99/100	99/100	100/100	100/100	100/100	99/100	100/100	99/100	99/100	95/99	96/100	99/100	IV	Chicken, UK
16	SS_2230	FBEI01000000	10	99/100	99/100	100/100	100/100	100/100	99/100	100/100	99/100	99/100	95/99	96/100	99/100	IV	Chicken, UK
17	SS_2351	FBDV01000000	19	99/100	99/100	100/100	100/100	100/100	99/100	100/100	99/100	99/100	95/99	96/100	99/100	IV	Chicken, UK
18	SS_2315	FBEG01000000	39	99/100	99/100	100/100	100/100	100/100	99/100	100/100	99/100	99/100	95/99	96/100	99/100	IV	Chicken, UK
19	UNOR5482c	FBME01000000	23	99/100	99/100	100/100	100/100	100/100	99/100	100/100	99/100	99/100	95/99	95/100	99/100	IV	Chicken, UK
20	H121760164	FBQE01000000	23	99/100	99/100	100/100	100/100	100/100	99/100	99/100	99/100	99/100	95/99	96/100	99/100	IV	Human, UK
21	SS_2279	FBEW01000000	13	99/100	99/100	100/100	100/100	100/100	99/100	100/100	99/100	99/100	95/99	96/100	99/100	IV	Chicken, UK
22	SS_2325	FBEK01000000	23	99/100	99/100	100/100	100/100	100/100	99/100	100/100	99/100	99/100	95/99	96/100	99/100	IV	Chicken, UK
23	CCN264	FBMQ01000000	11	99/100	100/100	99/100	100/100	100/100	99/100	100/100	99/100	99/100	95/99	95/100	98/100	IV	Poultry Environmental, UK

24	BCW_7432	MJZU01000000	34	99/100	100/100	100/100	100/100	100/100	99/100	100/100	99/100	99/100	95/99	95/100	98/100	IV	Faeces, US
25	BCW_7435	MJZX01000000	19	99/100	100/100	100/100	100/100	100/100	99/100	100/100	99/100	99/100	95/99	95/100	98/100	IV	Faeces, US
26	BCW_6447	MJWL01000000	14	99/100	100/100	100/100	100/100	100/100	99/100	100/100	99/100	99/100	95/99	95/100	98/100	IV	Faeces, US
27	BCW_6448	MJWM01000000	14	99/100	100/100	100/100	100/100	100/100	99/100	100/100	99/100	99/100	95/99	95/100	98/100	IV	Faeces, US

LOS Class V

No.	Strain	Accession#	Contig#	4	5	6	7	8	9	10	11/11'	12	13	14	15	Gene*	Class	Host
1	SS_2286	FBDZ01000000	21	99/100	99/100	100/100	99/100	99/100	100/100	100/100	99/100	100/100	92/99	95/100	99/99		V	Chicken, UK
2	SS_2278	FBEL01000000	27	99/100	99/100	100/100	99/100	99/100	100/100	100/100	99/100	100/100	92/99	95/100	98/100		V	Chicken, UK
3	H132680116	FBOL01000000	34	99/100	99/100	100/100	99/100	99/100	99/100	96/100	99/100	100/100	93/99	95/100	98/99	99/100	V	Environmental water, UK
4	SS_2285	FBES01000000	35	99/100	99/100	100/100	99/100	99/100	100/100	100/100	99/100	100/100	92/99	95/100	98/100		V	Chicken, UK
5	SS_2266	FBEJ01000000	40	99/100	99/100	100/100	99/100	99/100	100/100	100/100	99/100	100/100	92/99	95/100	99/99		V	Chicken, UK
6	H121240502	FBPC01000000	27	100/100	99/100	100/100	99/100	99/100	99/100	96/100	99/100	100/100	93/99	95/100	99/100	100/100	V	Human, UK
7	SS_2349	FBDY01000000	35	99/100	99/100	100/100	99/100	99/100	100/100	100/100	99/100	100/100	92/99	95/100	98/100		V	Chicken, UK
8	SS_2249	FBFO01000000	5	99/100	99/100	100/100	99/100	99/100	100/100	100/100	99/100	100/100	92/99	95/100	98/100		V	Chicken, UK
9	SS_2284	FBDX01000000	49	99/100	99/100	100/100	99/100	99/100	100/100	100/100	99/100	100/100	92/99	95/100	99/99		V	Chicken, UK
10	SS_2276	FBEX01000000	40	99/100	99/100	100/100	99/100	99/100	100/100	100/100	99/100	100/100	92/99	95/100	99/99		V	Chicken, UK
11	H094560717	FBOM01000000	5	99/100	99/100	100/100	99/100	99/100	100/100	100/100	99/100	100/100	99/100	99/100	96/99		V	Environmental water, UK
12	SS_2352	FBEH01000000	10	99/100	99/100	100/100	99/100	99/100	100/100	100/100	99/100	100/100	92/99	95/100	98/100		V	Chicken, UK
13	p604A	FBPT01000000	18	99/100	99/100	100/100	99/100	99/100	98/100	100/100	99/100	100/100	99/100	99/100	96/99		V	Soil, UK
14	H114640463b	FBPS01000000	13	99/100	99/100	100/100	99/100	99/100	100/100	100/100	99/100	100/100	99/100	99/100	96/100		V	Human, UK
15	NCTC11353	FBNV01000000	29	99/100	99/100	100/100	99/100	99/100	99/100	100/100	99/100	100/100	99/100	99/100	96/100		V	Pig, UK
16	H075200514	FBLS01000000	19	99/100	99/100	100/100	99/100	99/100	100/100	100/100	99/100	100/100	99/100	99/100	96/100		V	Environmental water, UK
17	SS_2271	FBEU01000000	8	99/100	99/100	100/100	99/100	99/100	100/100	100/100	99/100	100/100	92/99	95/100	99/99		V	Chicken, UK
18	H061980521a	FBLK01000000	15	99/100	99/100	99/100	99/100	99/100	100/100	100/100	99/100	100/100	99/100	99/100	96/100		V	Environmental water, UK
19	P588B	FBKK01000000	17	99/100	99/100	100/100	99/100	99/100	99/100	100/100	99/100	100/100	99/100	99/100	96/99		V	Soil, UK

LOS Class VI

No.	Strain	Accession#	Contig#	4	5	6	7	8	9	10	11	12	31	32	14	15	Class	Host
1	84-2	AIMS01000000	17	99/100	100/100	100/100	100/100	100/100	100/100	100/100	99/100	100/100	100/100	99/100	99/100	100/100	VI	Swine, UK
2	1091	AIMV01000000	2, 61	100/100	100/100	100/100	100/100	94/100	100/100	100/100	100/100	100/100	100/100	100/100	100/100	100/100	VI	Bovine, US
3	LMG 9854	AINL01000000	54	99/100	100/100	100/100	100/100	100/100	100/100	100/100	99/100	100/100	100/100	100/100	100/100	100/100	VI	Human, Canada
4	CVM N44505F	LBDX01000000	2	100/100	100/100	100/100	100/100	100/100	100/100	100/100	100/100	100/100	100/100	100/100	100/100	100/100	VI	Cow, US
5	CVM N51226F	LBDL01000000	3, 4	100/100	100/100	100/100		100/100	100/100	100/100	100/100	100/100	100/100	100/100	100/100	100/100	VI	Cow, US
6	CVM N13165	JAJT01000000	12	99/100	99/100	100/100	100/100	99/100	100/100	99/100	99/100	98/100	100/100	99/100	98/100	96/100	VI	Chicken, US
7	CVM N20344	JAJU01000000	27, 38, 45	100/100	100/100	100/100		100/100	100/100	100/100	100/100	100/100		100/100	100/100	100/100	VI	Chicken, US
8	CVM N20402	JAJV01000000	1	100/100	100/100	100/100	100/100	100/100	100/100	100/100	100/100	100/100	100/100	100/100	100/100	100/100	VI	Chicken, US
9	UNAJC222	FBHF01000000	8	99/100	99/100	99/100	100/100	99/100	100/100	100/100	99/100	98/100	100/100	100/100	97/100	99/100	VI	Pig, UK
10	H130600457	FAYX01000000	18	99/100	99/100	98/100	100/100	99/100	100/100	100/100	99/100	99/100	100/100	100/100	97/100	99/100	VI	Human, UK
11	P474D	FBOC01000000	22	99/100	99/100	99/100	99/100	91/100	99/100	95/100	99/100	98/100	99/100	99/100	96/100	98/100	VI	Soil, UK
12	NCTC11437	FBHL01000000	8	99/100	99/100	100/100	100/100	99/100	100/100	99/100	99/100	98/100	100/100	99/100	97/100	96/100	VI	Human, UK
13	H043920292	FBPD01000000	9	99/100	99/100	98/100	100/100	99/100	100/100	100/100	99/100	99/100	100/100	100/100	97/100	99/100	VI	Human, UK
14	UNLL3.1	FBIK01000000	4	99/100	99/100	100/100	100/100	99/100	100/100	100/100	99/100	98/100	100/100	99/100	97/100	98/100	VI	Pig, UK
15	UNQMCIIS16	FBMK01000000	14	99/100	99/100	100/100	100/100	99/100	100/100	99/100	99/100	98/100	100/100	99/100	97/100	96/100	VI	Human, UK
16	BRISPIG3	FBHR01000000	14	99/100	99/100	100/100	100/100	99/100	100/100	100/100	99/100	98/100	100/100	99/100	97/100	96/100	VI	Pig, UK
17	H125280575	FBKU01000000	19	99/100	99/100	100/100	99/100	99/100	98/90	94/94	99/100	98/100	100/100	100/100	97/100	96/100	VI	Human, UK
18	20G12	LWIH01000000	17, 130, 132,133, 160, 169	99/100	99/76	73/100		99/100	100/100	96/96	99/100	98/100	100/100	100/100	96/100	99/100	VI	Milk, US
19	FNWR7B4_44	MCFS01000000	9	99/100	99/100	100/100	100/100	99/100	100/100	100/100	99/100	98/100	100/100	100/100	97/100	99/100	VI	Unknown
20	BCW_6913	MJZG01000000	22	99/100	99/100	100/100	100/100	99/100	100/100	100/100	99/100	98/100	100/100	99/100	98/100	96/100	VI	Faeces, US
21	BCW_7433	MJZV01000000	36	99/100	99/100	100/100	100/100	99/100	100/100	100/100	99/100	98/100	100/100	99/100	98/100	96/100	VI	Faeces, US
22	Tx40	MDCN01000000	8	100/100	100/100	100/100	100/100	100/100	100/100	100/100	100/100	100/100	100/100	100/100	100/100	100/100	VI	Cattle faeces, US

LOS Class VII

No.	Strain	Accession#	Contig#	4	5	6	7	8	9	10	11	33	34	35	36	13	14	15	Class	Host
1	OXC6309	CUJR01000000	1	99/100	99/100	100/100	99/100	100/100	100/100	99/100	100/100	100/100	99/100	99/100	100/100	100/100	98/100	99/100	VII	Faeces, UK
22 2	OXC6385	CUMN01000000	1	99/100	99/100	100/100	99/100	100/100	100/100	100/100	100/100	100/100	100/100	99/100	100/100	100/100	98/100	99/100	VII	Faeces, UK
3	OXC6376	CUMC01000000	2	99/100	99/100	100/100	99/100	100/100	100/100	99/100	100/100	100/100	99/100	99/100	100/100	100/100	98/100	99/100	VII	Faeces, UK
4	OXC6601	CUUO01000000	1	99/100	99/100	100/100	99/100	100/100	99/100	99/100	100/100	100/100	100/100	99/100	100/100	100/100	97/100	98/100	VII	Faeces, UK
5	OXC6551	CUVK01000000	1	100/100	99/100	100/100	99/100	90/99	94/99	98/94	100/100	100/100	99/100	99/100	100/100	100/100	97/100	99/100	VII	Faeces, UK
6	OXC6587	CUUB01000000	1	99/100	99/100	100/100	99/100	99/100	100/100	99/100	100/100	100/100	99/100	99/100	100/100	100/100	98/100	99/100	VII	Faeces, UK
7	OXC6426	CUOF01000000	1	99/100	99/100	100/100	99/100	100/100	100/100	99/100	100/100	100/100	99/100	99/100	100/100	100/100	98/100	99/100	VII	Faeces, UK
8	OXC6371	CULZ01000000	1	99/100	99/100	100/100	99/100	100/100	100/100	99/100	100/100	100/100	99/100	99/100	100/100	100/100	98/100	99/100	VII	Faeces, UK
9	OXC6434	CUON01000000	1	99/100	100/100	100/100	100/100	100/100	100/100	100/100	99/100	100/100	100/100	99/100	100/100	100/100	98/100	99/100	VII	Faeces, UK
10	CVMN41652	LBEQ01000000	4	99/100	99/100	100/100	99/100	100/100	100/100	99/100	100/100	100/100	99/100	99/100	100/100	100/100	98/100	99/100	VII	Chicken, US
11	CVMN49243	LBEH01000000	4	99/100	99/100	100/100	99/100	100/100	100/100	99/100	100/100	100/100	99/100	99/100	100/100	100/100	98/100	99/100	VII	Chicken, US
12	CVMN51619	LBEE01000000	2	99/100	99/100	100/100	99/100	100/100	100/100	99/100	100/100	100/100	99/100	99/100	100/100	100/100	98/100	99/100	VII	Chicken, US
13	CVMN8133	JOVB01000000	8	100/100	100/100	100/100	100/100	99/100	100/100	100/100	99/100	100/100	99/100	100/100	100/100	100/100	100/100	100/100	VII	Turkey, US
14	CVMN20320	JOVX01000000	2	99/100	99/100	100/100	99/100	100/100	100/100	99/100	100/100	100/100	99/100	99/100	100/100	100/100	98/100	99/100	VII	Chicken, US
15	H063900532	FBPQ01000000	18	99/100	99/100	99/100	99/100	100/100	100/100	100/100	100/100	100/100	99/100	99/100	100/100	100/100	99/100	99/100	VII	Environmental water, UK
16	SS_2294	FBDW01000000	7	99/100	100/100	100/100	100/100	100/100	100/100	100/100	99/100	100/100	99/100	99/100	100/100	100/100	98/100	99/100	VII	Human, UK
17	H103600372	FBOB01000000	21	99/100	99/100	100/100	99/100	100/100	100/100	99/100	100/100	100/100	99/100	99/100	100/100	100/100	98/100	99/100	VII	Human, UK
18	H043200357	FAYD01000000	15	99/100	99/100	99/100	99/100	99/100	100/100	99/100	100/100	100/100	100/100	99/100	100/100	100/100	98/100	99/100	VII	Human, UK
19	H121060205	FBOW01000000	9	99/100	99/100	100/100	99/100	99/100	100/100	99/100	100/100	100/100	99/100	99/100	99/100	100/100	98/100	99/100	VII	Human, UK
20	UNOR4451c	FBMH01000000	60	99/100	99/100	100/100	99/100	100/100	100/100	99/100	100/100	100/100	99/100	99/100	100/100	100/100	98/100	99/100	VII	Chicken, UK
21	UNOR532A	FBIJ01000000	32	99/100	99/100	100/100	99/100	100/100	100/100	99/100	100/100	100/100	99/100	99/100	100/100	100/100	98/100	99/100	VII	Chicken, UK
22	H132600169	FBAF01000000	28	99/100	99/100	100/100	99/100	100/100	99/100	99/100	100/100	100/100	99/100	99/100	100/100	100/100	97/100	99/100	VII	Environmental water, UK
23	SWAN331	FBGR01000000	39	93/100	93/100	98/100	99/100	99/100	96/100	99/100	99/100	99/100	99/100	96/100	91/99	98/100	96/100	93/100	VII	Duck, UK
24	SS_2295	FBDS01000000	8	99/100	99/100	100/100	99/100	100/100	99/100	99/100	100/100	100/100	99/100	99/100	100/100	100/100	97/100	99/100	VII	Chicken, UK
25	SWAN195-1	FBGK01000000	46	93/100	93/100	98/100	99/100	99/100	96/100	99/100	99/100	99/100	99/100	96/100	91/99	98/100	96/100	93/100	VII	Duck, UK
26	UNOR7592c	FBMC01000000	19	99/100	99/100	100/100	99/100	100/100	100/100	99/100	100/100	100/100	99/100	99/100	100/100	100/100	98/100	99/100	VII	Chicken, UK
27	H084040382a	FBJY01000000	22	99/100	99/100	99/100	99/100	100/100	100/100	100/100	100/100	100/100	99/100	99/100	100/100	100/100	99/100	99/100	VII	Human, UK
28	H105280404	FAZS01000000	22	99/100	100/100	100/100	100/100	100/100	100/100	100/100	99/100	100/100	99/100	99/100	100/100	100/100	98/100	99/100	VII	Environmental water, UK
29	H081820599a	FAZL01000000	35	99/100	99/100	100/100	99/100	100/100	99/100	99/100	100/100	100/100	99/100	99/100	100/100	100/100	97/100	99/100	VII	Environmental water, UK
30	BCW_5918	MJVY01000000	38	99/100	99/100	100/100	99/100	100/100	99/100	100/100	100/94	100/100	99/100	99/100	100/100	99/100	97/100	99/100	VII	Faeces, US
31	BCW_4454	MJVZ01000000	39	99/100	99/100	100/100	99/100	100/100	99/100	100/100	100/94	100/100	99/100	99/100	100/100	99/100	97/100	99/100	VII	Faeces, US
32	BCW_6450	MJWA01000000	24	99/100	99/100	100/100	99/100	100/100	99/100	100/100	100/94	100/100	99/100	99/100	100/100	99/100	97/100	99/100	VII	Faeces, US
33	BCW_6949	MJZJ01000000	41	99/100	99/100	100/100	99/100	89/99	98/99	99/100	100/100	100/100	99/100	99/100	100/100	100/100	95/100	97/100	VII	Faeces, US
34	BCW_6951	MJZM01000000	9	99/100	99/100	100/100	99/100	100/100	99/100	100/100	100/94	100/100	99/100	99/100	100/100	99/100	97/100	99/100	VII	Faeces, US
35	BCW_6957	MJZR01000000	21	86/97	99/100	100/100	99/100	100/100	100/100	99/100	99/100	99/100	99/100	99/100	100/100	100/100	98/100	99/100	VII	Faeces, US
36	CAM962	BDRY01000000	7	99/100	99/100	100/100	99/100	100/100	100/100	99/100	100/100	100/100	99/100	99/100	100/100	100/100	98/100	99/100	VII	Faeces, Japan
37	BCW_4455	MJWE01000000	10	86/97	99/100	100/100	99/100	100/100	100/100	99/100	99/100	99/100	99/100	99/100	100/100	100/100	98/100	99/100	VII	Faeces, US
38	CVM 41971	JAJS01000000	23, 47, 38	99/100	99/100	100/100	99/100		100/100	99/100	99/100	100/100	99/100	99/100	100/100	100/100	98/100	99/100	VII	Human, US

LOS Class VIII

No.	Strain	Accession #	Contig#	4	5	6	7	8	9	10	11	12	13	14	15	16	17	Gene*	Class	Host
1	CVM N29716	ANMS01000000	1	100/100	100/100	100/100	100/100	100/100	100/100	100/100	100/100	100/100	100/100	100/100	100/100	100/100	100/100	100/100	VIII	Chicken, US
2	OXC6424	CUOD01000000	1	100/100	100/100	99/100	100/100	100/100	99/100	100/100	99/100	100/100	100/100	100/100	100/100	99/100	99/100	100/100	VIII	Faeces, UK
3	OXC6630	CUVQ01000000	1	98/100	100/100	100/100	100/100	100/100	100/100	100/100	100/100	99/100	100/100	100/100	100/100	100/100	100/100	99/100	VIII	Faeces, UK
4	OXC6568	CUTH01000000	1	99/100	100/100	100/100	100/100	100/100	100/100	100/100	100/100	100/100	100/100	100/100	100/100	99/100	100/100		VIII	Faeces, UK
5	OXC6343	CULD01000000	1	100/100	100/100	100/100	100/100	100/100	100/100	100/100	100/100	100/100	100/100	100/100	100/100	100/100	100/100	94/100	VIII	Faeces, UK
6	OXC6577	CUTR01000000	1	99/100	100/100	100/100	100/100	100/100	100/100	100/100	100/100	100/100	100/100	100/100	100/100	99/100	100/100		VIII	Faeces, UK
7	OXC6576	CUTQ01000000	2	99/100	100/100	100/100	100/100	100/100	100/100	100/100	100/100	100/100	99/100	100/100	100/100	100/100	99/100	91/100	VIII	Faeces, UK
8	OXC6400	CUNC01000000	1	100/100	100/100	100/100	100/100	100/100	100/100	100/100	100/100	100/100	100/100	100/100	100/100	100/100	100/100		VIII	Faeces, UK
9	CVM N45963	LBEK01000000	4	100/100	100/100	100/100	100/100	100/100	100/100	100/100	100/100	100/100	100/100	100/100	100/100	100/100	100/100	100/100	VIII	Chicken, US
10	CVM N44406F	LBDY01000000	2	100/100	100/100	100/100	100/100	100/100	100/100	100/100	100/100	100/100	100/100	100/100	100/100	100/100	100/100	100/100	VIII	Turkey, US
11	CVM N44984F	LBDW01000000	7	100/100	100/100	100/100	100/100	100/100	100/100	100/100	100/100	100/100	100/100	100/100	100/100	100/100	100/100	100/100	VIII	Chicken, US
12	CVM N47608F	LBDS01000000	2	100/100	100/100	100/100	100/100	100/100	100/100	100/100	100/100	100/100	100/100	100/100	100/100	100/100	100/100	100/100	VIII	Chicken, US
13	CVM N48647F	LBDR01000000	3	100/100	100/100	100/100	100/100	100/100	100/100	100/100	100/100	100/100	100/100	100/100	100/100	100/100	100/100	100/100	VIII	Chicken, US
14	CVM N49369F	LBDQ01000000	35,59	100/100	99/100	100/100	100/100	100/100	100/100	100/100	100/100	100/100	100/100	100/100	100/100	100/100	100/100	100/100	VIII	Chicken, US
15	CVM N51183F	LBDN01000000	4	100/100	100/100	100/100	100/100	100/100	100/100	100/100	100/100	99/100	100/100	100/100	100/100	100/100	100/100	100/100	VIII	Chicken, US

16	CVM N39665	LBEV01000000	10	100/100	100/100	100/100	100/100	100/100	100/100	100/100	100/100	100/100	100/100	100/100	100/100	100/100	100/100	100/100	VIII	Chicken, US
17	CVM N39671	LBEU01000000	8	100/100	100/100	100/100	100/100	100/100	100/100	100/100	100/100	100/100	100/100	100/100	100/100	100/100	100/100	100/100	VIII	Chicken, US
18	CVM N39677	LBET01000000	2,8	100/100	100/100	100/100	100/100	100/100	100/100	100/100	100/100	100/100	100/100	100/100	100/100	100/100	100/100	100/100	VIII	Chicken, US
19	CVM N40944	LBES01000000	6, 15	100/100	100/100	100/100	100/100	100/100	100/100	100/100	100/100	100/100	99/100	100/100	100/100	100/100	100/100	100/100	VIII	Chicken, US
20	CVM N40946	LBER01000000	2	100/100	100/100	100/100	100/100	100/100	100/100	100/100	100/100	100/100	100/100	100/100	100/100	100/100	100/100	100/100	VIII	Chicken, US
21	CVM N41661	LBEP01000000	6	100/100	100/100	100/100	100/100	100/100	100/100	100/100	100/100	100/100	100/100	100/100	100/100	100/100	100/100	100/100	VIII	Chicken, US
22	CVM N47960	LBEI01000000	1	100/100	100/100	100/100	100/100	100/100	100/100	100/100	100/100	100/100	100/100	100/100	100/100	100/100	100/100	100/100	VIII	Chicken, US
23	CVM N51712	LBEB01000000	6	100/100	100/100	100/100	100/100	100/100	100/100	100/100	100/100	100/100	100/100	100/100	100/100	100/100	100/100	100/100	VIII	Chicken, US
24	CVM N51987	LBEA01000000	2	100/100	100/100	100/100	100/100	100/100	100/100	100/100	100/100	100/100	100/100	100/100	100/100	100/100	100/100	100/100	VIII	Chicken, US
25	CVM N44396F	LBDZ01000000	2	100/100	100/100	100/100	100/100	100/100	100/100	100/100	100/100	100/100	100/100	100/100	100/100	100/100	100/100	100/100	VIII	Chicken, US
26	CVM N46596F	LBDT01000000	4	100/100	100/100	100/100	100/100	100/100	100/100	100/100	100/100	100/100	100/100	100/100	100/100	100/100	100/100	100/100	VIII	Chicken, US
27	CVM N50039F	LBDP01000000	4	100/100	100/100	100/100	100/100	100/100	100/100	100/100	100/100	100/100	100/100	100/100	100/100	100/100	100/100	100/100	VIII	Chicken, US
28	CVM N51201F	LBDM01000000	3, 4	100/100	100/100	100/100	100/100	100/100	100/100	100/100	100/100	100/100	100/100	100/100	100/100	100/100	100/100	100/100	VIII	Chicken, US
29	CVM 41917	JAJN01000000	2	100/100	99/100	100/100	100/100	100/100	100/100	100/100	100/100	100/100	100/100	100/100	100/100	100/100	100/100	94/100	VIII	Human, US
30	CVM 41932	JAJW01000000	1	100/100	100/100	100/100	100/100	100/100	100/100	100/100	100/100	100/100	100/100	100/100	100/100	100/100	100/100	100/100	VIII	Human, US
31	CVM 41939	JAJX01000000	6	100/100	100/100	100/100	100/100	100/100	100/100	100/100	100/100	100/100	100/100	100/100	100/100	100/100	100/100	100/100	VIII	Human, US
32	CVM 41955	JAJY01000000	2	100/100	100/100	100/100	100/100	100/100	100/100	100/100	100/100	100/100	100/100	100/100	100/100	100/100	100/100	100/100	VIII	Human, US
33	CVM 41958	JAKA01000000	2	100/100	100/100	100/100	100/100	100/100	100/100	100/100	100/100	100/100	100/100	100/100	100/100	100/100	100/100	100/100	VIII	Human, US
34	CVM 41965	JAJI01000000	6	100/100	100/100	100/100	100/100	100/100	100/100	100/100	100/100	100/100	100/100	100/100	100/100	100/100	100/100	100/100	VIII	Human, US
35	CVM 41976	JAKB01000000	13	100/100	100/100	100/100	100/100	100/100	100/100	100/100	100/100	100/100	100/100	100/100	100/100	100/100	100/100	100/100	VIII	Human, US
36	CVM 41986	JAJJ01000000	21	100/100	100/100	100/100	100/100	100/100	100/100	100/100	100/100	100/100	100/100	100/100	100/100	100/100	100/100	100/99	VIII	Human, US
37	CCN178	FBHU01000000	4	100/100	100/100	100/100	100/100	100/100	100/100	100/100	100/100	100/100	99/100	100/100	100/100	100/100	100/100	100/100	VIII	Poultry Farm environment, UK
38	H131800148	FAYF01000000	4	100/100	100/100	100/100	100/100	100/100	100/100	100/100	100/100	100/100	99/100	100/100	100/100	100/100	100/100	94/100	VIII	Human, UK
39	CCN397	FBGV01000000	15	100/100	100/100	100/100	100/100	100/100	100/100	100/100	100/100	100/100	100/100	100/100	100/100	100/100	100/100	100/100	VIII	Poultry Farm environment, UK
40	H054900335	FBQT01000000	6	100/100	100/100	100/100	100/100	100/100	100/100	100/100	100/100	100/100	100/100	100/100	100/100	100/100	100/100	100/100	VIII	Human, UK
41	P495D	FBLV01000000	8	100/100	100/100	100/100	100/100	100/100	100/100	100/100	100/100	100/100	100/100	100/100	100/100	100/100	100/100	100/100	VIII	Soil, UK
42	H095340114b	FBKC01000000	12	100/100	100/100	100/100	100/100	100/100	99/100	100/100	100/100	99/100	100/100	100/100	100/100	100/100	100/100	99/100	VIII	Human, UK
43	H093960099	FBPM01000000	6	100/100	100/100	100/100	100/100	100/100	100/100	100/100	100/100	99/100	100/100	100/100	100/100	100/100	100/100	99/100	VIII	Human, UK
44	P515A	FBAM01000000	17	100/100	100/100	100/100	100/100	100/100	100/100	100/100	100/100	100/100	100/100	100/100	100/100	100/100	100/100	100/100	VIII	Soil, UK
45	CCN289	FBMU01000000	1	100/100	100/100	100/100	100/100	100/100	100/100	100/100	100/100	100/100	100/100	100/100	100/100	100/100	100/100	100/100	VIII	Poultry farm water, UK
46	H051080182	FBOQ01000000	16	100/100	100/100	100/100	100/100	100/100	100/100	100/100	100/100	100/100	100/100	100/100	100/100	100/100	100/100	100/100	VIII	Human, UK
47	H093580323	FBON01000000	4	100/100	100/100	100/100	100/100	100/100	100/100	100/100	100/100	99/100	100/100	100/100	100/100	100/100	100/100	99/100	VIII	Environmental Water, UK
48	CCN257	FBHS01000000	5	100/100	100/100	100/100	100/100	100/100	100/100	100/100	100/100	100/100	100/100	100/100	100/100	100/100	100/100	94/100	VIII	Poultry farm, faeces, UK
49	H042120298	FBPV01000000	30	100/100	100/100	100/100	100/100	100/100	100/100	100/100	100/100	100/100	100/100	100/100	100/100	100/100	100/100	94/100	VIII	Human, UK
50	H043940500	FBQK01000000	5	99/100	100/100	100/100	100/100	100/100	100/100	100/100	100/100	100/100	100/100	100/100	100/100	100/100	100/100	97/100	VIII	Human, UK
51	H112820480	FBPF01000000	8	100/100	100/100	100/100	100/100	100/100	100/100	100/100	100/100	100/100	100/100	100/100	100/100	100/100	99/100	99/100	VIII	Human, UK
52	CCN19	FBJI01000000	20	100/100	100/100	100/100	100/100	100/100	100/100	100/100	100/100	100/100	100/100	100/100	100/100	100/100	100/100	100/100	VIII	Farm Environment, UK
53	SWAN269	FBLP01000000	18	88/100	98/100	93/99	93/100	95/100	95/100	92/100	94/100	95/100	95/100	95/100	96/100	91/100	91/100		VIII	Duck, UK
54	CCN355	FBIR01000000	15	100/100	100/100	100/100	100/100	100/100	100/100	100/100	100/100	100/100	100/100	100/100	100/100	100/100	99/100	94/100	VIII	Poultry farm, faeces, UK
55	H123080386	FBKI01000000	10, 12	100/100	100/100	100/100	100/100	100/100	100/100	100/100	100/100	100/100	100/100	100/100	100/100	100/100	100/100	100/100	VIII	Human, UK
56	P515B	FBKE01000000	5	100/100	100/100	100/100	100/100	100/100	100/100	100/100	100/100	100/100	100/100	100/100	100/100	100/100	100/100	100/100	VIII	Soil, UK
57	CCN177	FBHT01000000	6	100/100	100/100	100/100	100/100	100/100	100/100	100/100	100/100	100/100	99/100	100/100	100/100	100/100	100/100	100/100	VIII	Farm Environment, UK
58	H133020651a	FAYK01000000	12	100/100	100/100	100/100	100/100	100/100	100/100	100/100	100/100	100/100	100/100	100/100	100/100	100/100	100/100	100/100	VIII	Human, UK
59	H140420240	FBMS01000000	8	100/100	100/100	100/100	100/100	100/100	100/100	100/100	100/100	100/100	100/100	100/100	100/100	100/100	100/100	100/100	VIII	Human, UK
60	P474B	FBKX01000000	6	100/100	100/100	100/100	100/100	100/100	100/100	100/100	100/100	99/100	100/100	100/100	100/100	100/100	100/100	100/100	VIII	Soil, UK
61	H095340114a	FAZE01000000	15	100/100	100/100	100/100	100/100	100/100	99/100	100/100	100/100	99/100	100/100	100/100	100/100	100/100	100/100	99/100	VIII	Human, UK
62	H083960630	FBNN01000000	6	100/100	100/100	100/100	100/100	100/100	100/100	100/100	100/100	100/100	99/100	100/100	100/100	100/100	100/100	99/100	VIII	Human, UK
63	CCN205	FBHO01000000	19	100/100	100/100	100/100	100/100	100/100	100/100	100/100	100/100	100/100	100/100	100/100	100/100	100/100	100/100	100/100	VIII	Poultry Farm Water, UK
64	P546C	FBLN01000000	5	100/100	100/100	100/100	100/100	100/100	100/100	100/100	100/100	100/100	100/100	100/100	100/100	100/100	100/100	94/100	VIII	Soil, UK
65	SWAN350	FBLU01000000	12	100/100	100/100	100/100	100/100	100/100	100/100	100/100	100/100	100/100	100/100	100/100	100/100	100/100	100/100	100/100	VIII	Duck, UK
66	CCN357	FBJL01000000	14	100/100	100/100	100/100	100/100	100/100	100/100	100/100	100/100	100/100	100/100	100/100	100/100	100/100	99/100	100/100	VIII	Poultry Farm faeces, UK
67	H132480140	FBQA01000000	15	100/100	100/100	100/100	99/100	100/100	100/100	100/100	100/100	100/100	100/100	100/100	100/100	100/100	100/100	100/100	VIII	Environmental Water, UK
68	SS_2322	FBFP01000000	6	99/100	100/100	100/100	100/100	100/100	100/100	100/100	100/100	100/100	100/100	100/100	100/100	100/100	99/100	91/100	VIII	Chicken, UK
69	H133020651b	FBLB01000000	13	100/100	100/100	100/100	100/100	100/100	100/100	100/100	100/100	100/100	100/100	100/100	100/100	100/100	100/100	100/100	VIII	Human, UK
70	P495B	FAXU01000000	11	100/100	100/100	100/100	100/100	100/100	100/100	100/100	100/100	100/100	100/100	100/100	100/100	100/100	100/100	100/100	VIII	Soil, UK
71	H140420134	FBII01000000	18	99/100	100/100	100/100	100/100	100/100	100/100	100/100	100/100	100/100	100/100	100/100	100/100	99/100	100/100	94/100	VIII	Human, UK
72	H092260570	FBQR01000000	18	100/100	100/100	100/100	100/100	100/100	100/100	100/100	100/100	100/100	100/100	100/100	100/100	100/100	100/100	100/100	VIII	Environmental Water, UK
73	P515D	FAZH01000000	15	100/100	100/100	100/100	100/100	100/100	100/100	100/100	100/100	100/100	100/100	100/100	100/100	100/100	100/100	100/100	VIII	Soil, UK
74	P588D	FBLA01000000	4	100/100	100/100	100/100	100/100	100/100	100/100	100/100	100/100	100/100	100/100	100/100	100/100	100/100	100/100	100/100	VIII	Soil, UK

75	CCN245	FBGW01000000	6	100/100	100/100	100/100	100/100	100/100	100/100	100/100	100/100	100/100	99/100	100/100	100/100	100/100	100/100	100/100	VIII	Poultry Farm faeces, UK
76	H134460277	FBPG01000000	11	99/100	100/100	100/100	100/100	100/100	100/100	100/100	100/100	100/100	100/100	100/100	100/100	100/100	99/100	91/100	VIII	Human, UK
77	SWAN195-3	FBLI01000000	4	100/100	100/100	100/100	100/100	100/100	99/100	100/100	100/100	99/100	100/100	100/100	100/100	100/100	100/100	99/100	VIII	Duck, UK
78	P494D	FBMB01000000	4	99/100	100/100	100/100	99/100	100/100	100/100	100/100	100/100	100/100	100/100	100/100	100/100	100/100	100/100	100/100	VIII	Soil, UK
79	H090520713	FBOZ01000000	9	99/100	100/100	100/100	100/100	100/100	100/100	100/100	100/100	100/100	100/100	100/100	100/100	100/100	99/100	84/99	VIII	Environmental Water, UK
80	CCN246	FBHD01000000	14	100/100	100/100	100/100	100/100	100/100	100/100	100/100	100/100	100/100	99/100	100/100	100/100	100/100	100/100	100/100	VIII	Poultry Farm faeces, UK
81	NCTC12568	FBNI01000000	24	99/100	100/100	100/100	100/100	100/100	100/100	100/100	100/100	100/100	99/100	100/100	100/100	99/100	100/100	100/100	VIII	Pig, UK
82	H054000444	FBOO01000000	28	99/100	100/100	100/100	100/100	100/100	100/100	100/100	100/100	100/100	100/100	100/100	100/100	100/100	99/100	90/100	VIII	Environmental Water, UK
83	P494B	FBAK01000000	15	100/100	100/100	100/100	100/100	100/100	100/100	100/100	100/100	100/100	100/100	100/100	100/100	100/100	100/100	100/100	VIII	Soil, UK
84	H102240161	FAYC01000000	13	100/100	100/100	100/100	100/100	100/100	99/100	100/100	100/100	99/100	100/100	100/100	100/100	100/100	100/100	99/100	VIII	Environmental Water, UK
85	H090660740	FBPI01000000	22	100/100		100/100	100/100	100/100	100/100	100/100	100/100	100/100	100/100	100/100	100/100	99/100	99/100	100/100	VIII	Environmental Water, UK
86	H054000445	FBPB01000000	15	100/100	100/100	100/100	99/100	100/100	100/100	100/100	100/100	100/100	100/100	100/100	100/100	100/100	100/100	100/100	VIII	Environmental Water, UK
87	NCTC12567	FBGX01000000	18	100/100	100/100	100/100	100/100	100/100	100/100	100/100	100/100	100/100	100/100	100/100	100/100	100/100	100/100	100/100	VIII	Human, UK
88	SWAN249	FBGQ01000000	23	88/100	98/100	93/99	93/100	95/100	95/100	92/100	94/100	95/100	95/100	95/100	96/100	91/100	91/100		VIII	Duck, UK
89	H070680142	FBNW01000000	9	100/100	100/100	100/100	100/100	100/100	100/100	100/100	100/100	100/100	100/100	100/100	100/100	100/100	100/85	100/100	VIII	Human, UK
90	SS_2329	FBFS01000000	10	99/100	100/100	100/100	100/100	100/100	100/100	100/100	100/100	100/100	100/100	100/100	100/100	100/100	99/100	91/100	VIII	Chicken, UK
91	P495c	FBQW01000000	13	100/100	100/100	100/100	100/100	100/100	100/100	100/100	100/100	99/100	100/100	100/100	100/100	100/100	99/100	99/100	VIII	Soil, UK
92	CCN20	FBJP01000000	6	100/100	100/100	100/100	100/100	100/100	100/100	100/100	100/100	100/100	99/100	100/100	100/100	100/100	100/100	100/100	VIII	Farm Environment, UK
93	UNCIC2	FBIW01000000	11	100/100	100/100	100/100	100/100	100/100	100/100	100/100	100/100	99/100	100/100	100/100	100/100	100/100	100/100	100/100	VIII	Chicken, UK
94	VA6	MPIQ01000000	2	88/100	98/100	93/99	93/100	96/100	95/100	92/`100	94/100	95/100	95/100	90/97	89/98	91/100	91/100		VIII	Raw water, Sweden
95	OXC6296	CUJE01000000	1	99/100	99/100	100/100	100/100	100/100	100/100	100/100	100/100	100/100	100/100	100/100	100/100	100/100	99/100	91/100	VIII	Faeces, UK
96	OXC6597	CUUK01000000	1	99/100	100/100	100/100	100/100	100/100	100/100	100/100	100/100	100/100	100/100	100/100	100/100	100/100	99/100	91/100	VIII	Faeces, UK
97	RC105	CYQJ01000000	15, 18	100/100	100/100		99/100	100/100	100/100	100/100	100/100	100/100	100/100	100/100	100/100	100/100	100/100	100/100	VIII	Supermarket, UK
98	RC148	CYQO01000000	4	100/100	100/100	100/100	99/100	100/100	100/100	100/100	100/100	100/100	100/100	100/100	100/100	100/100	99/100	100/100	VIII	Supermarket, UK
