# Supplementary material for: Molecular and in silico typing of the lipooligosaccharide biosynthesis gene cluster in Campylobacter jejuni and Campylobacter coli
Source: PLoS One. 2022 Mar 31;17(3):e0265585. doi: 10.1371/journal.pone.0265585 (PMC8970381; doi:10.1371/journal.pone.0265585)
Supplement: S4 Table — (PDF) [file pone.0265585.s004.pdf]

S4 Table. Summary of *C. coli* reference strains used to define LOS classes (Richards *et al*., 2013) in this study

The original study (Richards *et al*., 2013) used letters (A to H) to designate the 8 *C. coli* LOS classes, but Roman numerals (I to VIII) are now used to be consistent with the more recent literature (Skarp-de Haan *et al*., 2014; Culebro *et al*., 2018).

***C. coli* reference Strain**	**Accession Number (Contig No for LOS sequence)**	**LOS Class**	**Identified novel LOS gene position**
C. coli LMG2336	AINM01000000 (Contig 29)	I	AINM01000029 45451-46905
C. coli 202/04	AINH01000000 (Contig 4)	II	AINH01000004 24584-25144
C. coli LMG23341	AINN01000000 (Contig 20)	III	AINN01000020 42860- 43621
C. coli 1948	AINE00000000 (Contig 24)	IV	-
C. coli 1957	AINF01000000 (Contig 1)	V	AINF01000001 35579-36334
C. coli 1148	AIMX00000000 (Contig 1)	VI	-
C. coli LMG9853	AINR00000000 (Contig 1)	VII	-
