# Supplementary material for: Molecular and in silico typing of the lipooligosaccharide biosynthesis gene cluster in Campylobacter jejuni and Campylobacter coli
Source: PLoS One. 2022 Mar 31;17(3):e0265585. doi: 10.1371/journal.pone.0265585 (PMC8970381; doi:10.1371/journal.pone.0265585)
Supplement: S5 Table — (PDF) [file pone.0265585.s005.pdf]

S5 Table. Summary of LOS locus typing *C. jejuni* clinical isolates (collected from the Northampton General Hospital in the current study) and PCR products' sequencing results

No.	Clinical *C. jejuni* strain	PCR with optimised primers										PCR with primers specific to other classes	Sequencing of PCR products		Identified LOS Class
		A1	A2	B1	B2	C	26EO	F	G	26HP	28EP		PCR product seq. identity (%)	Seq. compatible reference strain/ LOS genes	
1	CJ10	+	-	-	-	-	-	-	-	-	-	-	99	RM1048 *cgtA* & *cgtB*	A1
2	CJ4	-	-	-	+	-	-	-	-	-	-	-	98	81-176 *cgtA* & *cgtB*	B2
3	CJ12	-	-	-	-	-	-	-	-	-	-	-			Non-typeable
4	CJ13	-	+	-	-	-	-	-	-	-	-	-	98	RM1556 *cgtA* & *cgtB*	A2
5	CJ18	-	+	-	-	-	-	-	-	-	-	-	97	RM1556 *cgtA* & *cgtB*	A2
6	CJ23	-	-	-	-	+	-	-	-	-	-	-	96	11168 *neuA1* & *neuC1*	C
7	S1	-	-	-	-	-	-	+	-	-	-	-	95	RM1221 *cgtD* & *waaV*	F
8	S2	-	-	-	-	-	-	-	-	+	±	-			H/P
9	101	-	-	+	-	-	-	-	-	-	-	-			B1
10	102	-	-	-	-	-	-	-	-	+	+	-			P
11	103	-	-	-	-	-	+	-	-	-	-	-			O
12	104	-	-	-	-	+	-	-	-	-	-	-	98	11168 *neuA1* & *neuC1*	C
13	105	-	-	-	-	-	-	-	-	+	-	-			H
14	106	-	-	-	-	-	-	-	-	+	+	-			P
15	Moulton	-	-	-	-	-	-	-	-	-	-	-			Non-typeable
16	1336	-	-	-	-	-	-	-	-	-	-	-			Novel
17	92740	-	-	-	-	-	-	-	-	+	+	-	99	4031 Orf27 & Orf28	P
18	92691	-	+	+	-	-	-	-	-	-	-	-			A2/B1
19	92540	-	-	-	-	-	-	-	-	+	-	-			H

20	92717	-	-	-	-	**+**	-	-	-	-	-	-	98	11168<br>*neuA1* & *neuC1*	C
21	93133Y	-	-	-	-	-	-	-	-	**+**	**±**	-			H/P
22	CJ20	-	-	-	-	**+**	-	-	-	-	-	-	97	11168<br>*neuA1* & *neuC1*	C
23	93084N	-	-	-	**+**	-	-	-	-	-	-	-	99		B2
24	92661	-	-	-	-	-	-	-	-	**+**	**+**	-	98		P
25	112990	-	-	-	-	-	-	-	-	-	-	-			Non-typeable
26	118715	-	-	-	-	-	-	-	-	**+**	**±**	-	98	4031<br>Orf27 & Orf28	H/P
27	118973	-	**+**	-	**+**	-	-	-	-	-	-	-	94<br>99	RM1556<br>*cgtA* & *cgtB*<br>81-176<br>*cgtA* & *cgtB*	A2/B2
28	512	-	-	-	-	-	-	-	-	-	-	-			Non-typeable
29	121097	-	-	-	**+**	-	-	-	-	-	-	-	99	81-176<br>*cgtA* & *cgtB*	B2
30	118718	-	-	-	-	**+**	-	-	-	-	-	-	98	11168<br>*neuA1* & *neuC1*	C
31	93941P	-	-	-	-	-	-	-	-	-	-	-			Non-typeable
32	11168Δ32-52	-	-	-	-	-	-	-	-	-	-	-			-ive control
33	ME113262	-	-	-	-	**+**	-	-	-	-	-	-	99	11168<br>*neuA1* & *neuC1*	C
34	ME112938	-	-	-	-	-	-	-	-	-	-	-			Non-typeable
35	ME112946	-	-	-	**+**	-	-	-	-	-	-	-			B2
36	ME112990	-	-	-	-	-	-	-	-	-	-	-			Non-typeable
37	ME113179	-	-	-	**+**	-	-	-	-	-	-	-	95		B2
38	ME113090	-	-	-	-	-	-	-	-	-	-	-			Non-typeable
39	751	-	-	-	-	-	-	-	-	-	-	-			Non-typeable
40	92649	-	-	-	-	-	-	-	-	**+**	**+**	-			P
41	36670	-	-	-	-	-	-	**+**	-	-	-	-	96		F
42	CJ3111	-	**+**	-	-	-	-	-	-	-	-	-			A2
43	37531	-	**+**	-	-	-	-	-	-	-	-	-			A2
44	34218	-	-	-	-	**+**	-	-	-	-	-	-	98	11168<br>*neuA1* & *neuC1*	C

45	34806	-	-	-	+	-	-	-	-	-	-	-	98	81-176<br>*cgtA* & *cgtB*	B2
46	38625	-	-	-	-	-	-	-	-	+	+	-	99	4031<br>Orf27 & Orf28	P
47	34565	-	-	-	-	+	-	-	-	-	-	-	99	11168<br>*neuA1* & *neuC1*	C
48	38608	-	-	-	-	-	+	-	-	-	-	-			E
49	44406	-	-	-	+	-	-	-	-	-	-	-			B2
50	45283	-	-	-	-	+	-	-	-	-	-	-	99	11168<br>*neuA1* & *neuC1*	C
51	41999	-	-	-	+	-	-	-	-	-	-	-	99	81-176<br>*cgtA* & *cgtB*	B2
52	40973	+	-	-	-	-	-	-	-	-	-	-	98	RM1048<br>*cgtA* & *cgtB*	A1
53	47185	-	-	-	-	-	-	-	-	+	+	-			P
54	39864	-	-	-	-	-	-	-	-	+	+	-			P
55	60319	-	-	-	-	+	-	-	-	-	-	-			C
56	60238	-	-	-	-	-	-	-	-	-	-	-			Non-typeable
57	54386	-	-	-	+	+	-	-	-	-	-	-	98<br>98	81-176<br>*cgtA* & *cgtB*	B2/C
58	50702	-	-	-	-	-	-	+	-	-	-	-	92	RM1221<br>*cgtD* & *waaV*	F
59	59653	+	-	-	-	-	-	-	-	-	-	-	98	RM1048<br>*cgtA* & *cgtB*	A1
60	51585	-	-	-	-	-	-	-	-	+	-	-			H
61	92838	-	-	+	-	-	-	-	-	-	-	-			B1
62	92871	-	-	+	-	-	-	-	-	-	-	-			B1
